# Integrated Tree-Ring-Radiocarbon High-Resolution Timeframe to Resolve Earlier Second Millennium BCE Mesopotamian Chronology

**DOI:** 10.1371/journal.pone.0157144

**Published:** 2016-07-13

**Authors:** Sturt W. Manning, Carol B. Griggs, Brita Lorentzen, Gojko Barjamovic, Christopher Bronk Ramsey, Bernd Kromer, Eva Maria Wild

**Affiliations:** 1 Cornell Tree-Ring Laboratory, Department of Classics, B48 Goldwin Smith Hall, Cornell University, Ithaca, New York 14853–3201, United States of America; 2 Department of Near Eastern Languages & Civilizations, Harvard University, 6 Divinity Avenue, Cambridge, Massachusetts 02138, United States of America; 3 Oxford Radiocarbon Accelerator Unit, Research Laboratory for Archaeology & The History of Art, University of Oxford, Oxford OX1 3QY, United Kingdom; 4 Institute of Environmental Physics, University of Heidelberg, D-69120 Heidelberg, Germany; 5 University of Vienna, VERA Laboratory, Faculty of Physics, Isotope Research and Nuclear Physics, Währinger Straße 17, A-1090 Vienna, Austria; Chinese Academy of Sciences, CHINA

## Abstract

500 years of ancient Near Eastern history from the earlier second millennium BCE, including such pivotal figures as Hammurabi of Babylon, Šamši-Adad I (who conquered Aššur) and Zimrilim of Mari, has long floated in calendar time subject to rival chronological schemes up to 150+ years apart. Texts preserved on clay tablets provide much information, including some astronomical references, but despite 100+ years of scholarly effort, chronological resolution has proved impossible. Documents linked with specific Assyrian officials and rulers have been found and associated with archaeological wood samples at Kültepe and Acemhöyük in Turkey, and offer the potential to resolve this long-running problem. Here we show that previous work using tree-ring dating to place these timbers in absolute time has fundamental problems with key dendrochronological crossdates due to small sample numbers in overlapping years and insufficient critical assessment. To address, we have integrated secure dendrochronological sequences directly with radiocarbon (^14^C) measurements to achieve tightly resolved absolute (calendar) chronological associations and identify the secure links of this tree-ring chronology with the archaeological-historical evidence. The revised tree-ring-sequenced ^14^C time-series for Kültepe and Acemhöyük is compatible only with the so-called Middle Chronology and not with the rival High, Low or New Chronologies. This finding provides a robust resolution to a century of uncertainty in Mesopotamian chronology and scholarship, and a secure basis for construction of a coherent timeframe and history across the Near East and East Mediterranean in the earlier second millennium BCE. Our re-dating also affects an unusual tree-ring growth anomaly in wood from Porsuk, Turkey, previously tentatively associated with the Minoan eruption of the Santorini volcano. This tree-ring growth anomaly is now directly dated ~1681–1673 BCE (68.2% highest posterior density range), ~20 years earlier than previous assessments, indicating that it likely has no association with the subsequent Santorini volcanic eruption.

## Introduction

### Mesopotamian Chronology and History

**Fig 1:**
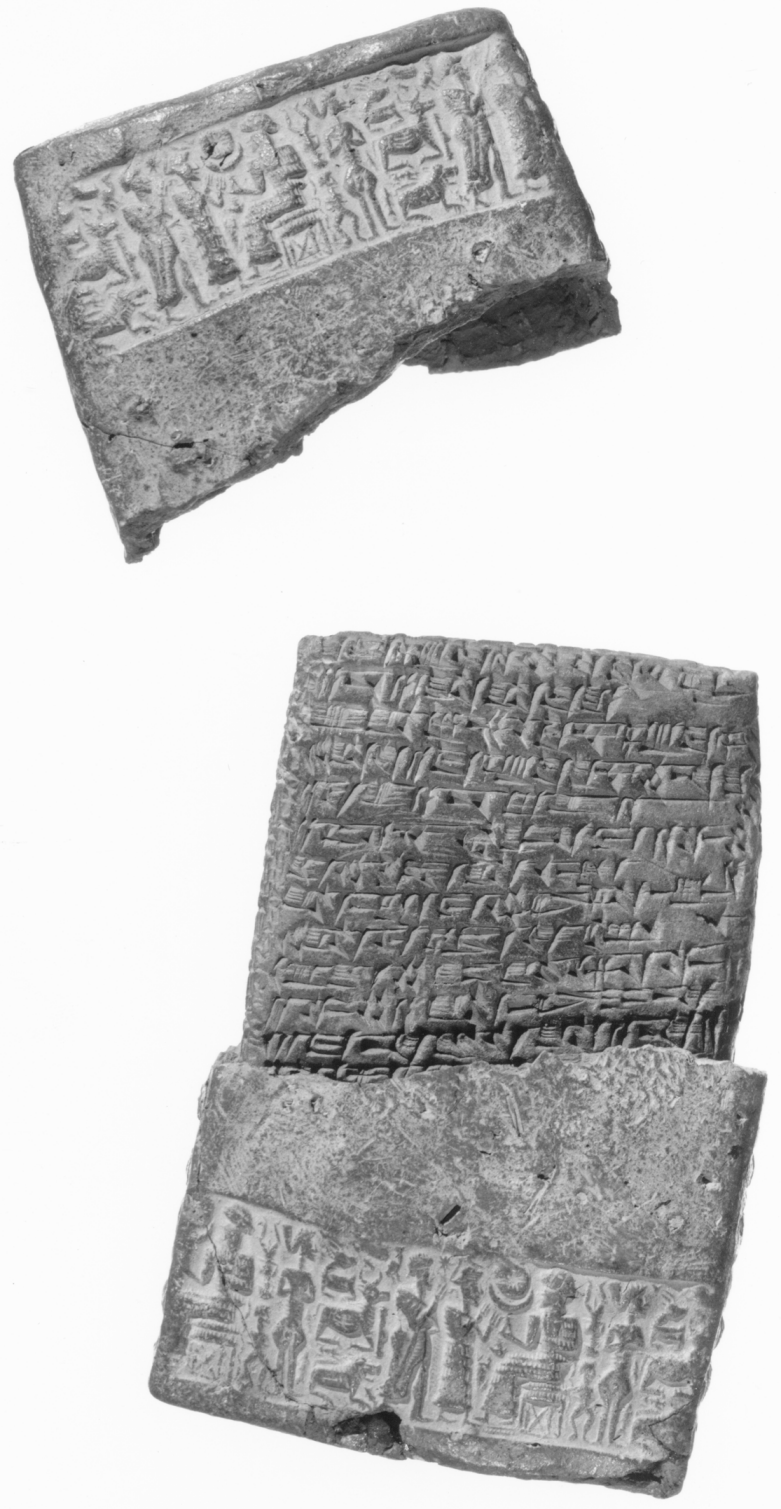
An example of an Old Assyrian clay tablet text: Yale Babylonian Collection, NBC 1905, showing a sealed Old Assyrian legal record.

A dense textual record preserved on clay tablets from the Ur III through Old Babylonian periods (~2070–1750 BCE) provides an extraordinary wealth of information on rulers, their families and connections, officials, wider society, warfare, trade, literature, religion, science and many other aspects of the history of this world which stretched from Mesopotamia into central Anatolia (e.g. [1–5]) (Fig 1). Basic biographies of key figures such as Hammurabi of Babylon and Šamši-Adad I can be extracted [6]. This record provides a detailed relative chronology linking named individuals and places, in particular through texts dated by annually appointed officials, or ‘eponyms’, in the city of Aššur [7], such that an annual timescale available from lists of these officials and the partly overlapping Mari Eponym Chronicles can be combined into a Revised Eponym List (REL) [5]. (Note: a short glossary of some terms and places is provided in Table 1.) There are of course complications. These include the fact that different manuscripts of the eponym list disagree slightly, and are imperfectly preserved, and that there are breaks in communication during winter between Aššur and Anatolia, all of which allow for varying reconstructions of the sequence and introduce a 0–5 year error potential [5, 8–11]. Nonetheless, the reconstructed REL record now available offers an approximately annual relative timeframe for the greater Mesopotamian world for the earlier second millennium BCE [5]. Alongside our knowledge of the Babylonian dynastic succession and the well-established synchronism of Šamši-Adad I’s death in Hammurabi’s 18th regnal year, this allows us to establish a relative chronological sequence of some 380 years between the ascent of the Assyrian ruler Erišum I and the destruction of Babylon during the Hittite invasion of Muršili I. However, this richly documented historical period floats unanchored in calendar time (e.g. [12, 13]). This alone is problematic, and prevents secure synchronization with contemporary civilizations in surrounding areas (such as Egypt).

**Table 1:** A Short Glossary of some terms and places used in this paper.

**Acemhöyük**	Archaeological site (38°24′ N, 33°50′E) comprising a city with no known ancient name with the remains of two large public structures belonging to the second half of the Assyrian Colony Period.
**Aššur**	City on the Tigris river north of Baghdad in modern-day Iraq; during the Middle Bronze Age (MBA) it was a small city state specialized in long-distance trade.
**Assyrian Colony Period**	Chronological term used to describe the early stage of the Middle Bronze Age (MBA) in Turkey (MBA1: ~MC 2000–1800 BCE) which produced the bulk of the written evidence for the Assyrian caravan trade at the site of Kültepe.
**Central Anatolia**	A region of Turkey (and its corresponding material culture) essentially defined by the Pontic Mountains in the north and the Taurus in the east, south and west.
**Eponym**	Term used to designate the main public office in Aššur. The official was appointed for one year at a time and gave his name to the year. An overlapping sequence (REL) of written ‘eponym lists’ that keep track of the sequence of officials served as dating tools for the Assyrians and form the backbone of our current internal chronology of the Assyrian Colony Period.
**Kültepe**	Archaeological site (38°51′N, 35°38′E) of the ancient city of Kaneš (or Kanesh). Location of the main settlement of Assyrian traders in Central Anatolia; the site has produced extensive remains of the merchant houses and their archival records in the form of ~23,000 inscribed clay tablets.
**Middle Bronze Age (MBA)**	Archaeological designation for the period ~2000–1600 BCE in Western Asia. In Turkey the period is subdivided into an early stage (MBA1 ~MC 2000–1800 BCE) and a later stage (MBA2: ~MC 1800–1600 BCE). The former corresponds to the Assyrian Colony Period.
**Middle Chronology (MC)**	The standard Middle Chronology (MC) that dates the Fall of Babylon to 1595 BC based on a solution of (less than perfect) astronomical evidence. Often used as the conventional scale to date events in ancient Western Asia. An alternative ‘Low’-MC (L-MC) is 8 years later. Other schemes using this same astronomical and other evidence (High Chronology, Low Chronology, New Chronology) offer alternatives from approximately +56/64 years older to –64/88 years later than the MC or Low-MC. This ~152 year date range, versus agreed closer definition, has been a major source of scholarly debate and controversy.
**Old Assyrian**	Term used about the chronological period ~MC 2000 to 1600 BCE in Iraq north of Baghdad (and sometimes also Central Turkey and/or Northern Syria) as well as the associated stage of the Assyrian dialect of the Akkadian language. Corresponds to the term "Middle Bronze Age" in archaeological parlance.
**Old Babylonian**	Term used about the chronological period ~MC 2000 to 1600 BCE in Iraq south of Baghdad (and sometimes also Northern Syria), as well as the associated stage of the Babylonian dialect of the Akkadian language. Corresponds to the term “Middle Bronze Age” in archaeological parlance.
**REL (Revised Eponym List)**	A reconstructed overlapping sequence of written eponyms covering a period of ~250 calendar years that forms the internal chronology of the Assyrian Colony Period.

Records with astronomical observations establish an absolute chronology for Mesopotamia back to the early 1^st^ millennium BCE, and provide an approximate (±10 years) chronology reaching to the late 15^th^ century BCE. However, there is no accepted timescale for the earlier second millennium BCE despite much work across several disciplines and over 100 years of scholarly controversy (e.g. [12–15]). Since the recognition of tablets containing observations of the planet Venus from the reign of Ammisaduqa of Babylon, there have been numerous attempts, either using these observations and/or other evidence (solar eclipses, textual data, archaeological materials or scientific dating), to try to provide absolute dates for the Old Babylonian and Old Assyrian periods (e.g. [5, 9–19]). Various chronologies, such as the so-called Ultra-High Chronology, High Chronology, Middle Chronology (with a standard ‘high’ Middle Chronology and an 8-year lower alternative Low-Middle Chronology), Low Chronology (or 8-year lower alternative), New Chronology and an Ultra-Low Chronology, as well as others forming variations in between or around those time frames, have been proposed and debated over many decades of work–with the range of dates varying for the end of the 1^st^ Babylonian Dynasty by up to ~234 years at extremes in scholarship since 1940, and 150+ years in more recent work [12]. Considerable chronological uncertainty has been the only outcome, limiting wider historical analysis of this key period.

### The Relevance of Dendrochronology to the MBA: potential and complications

**Fig 2:**
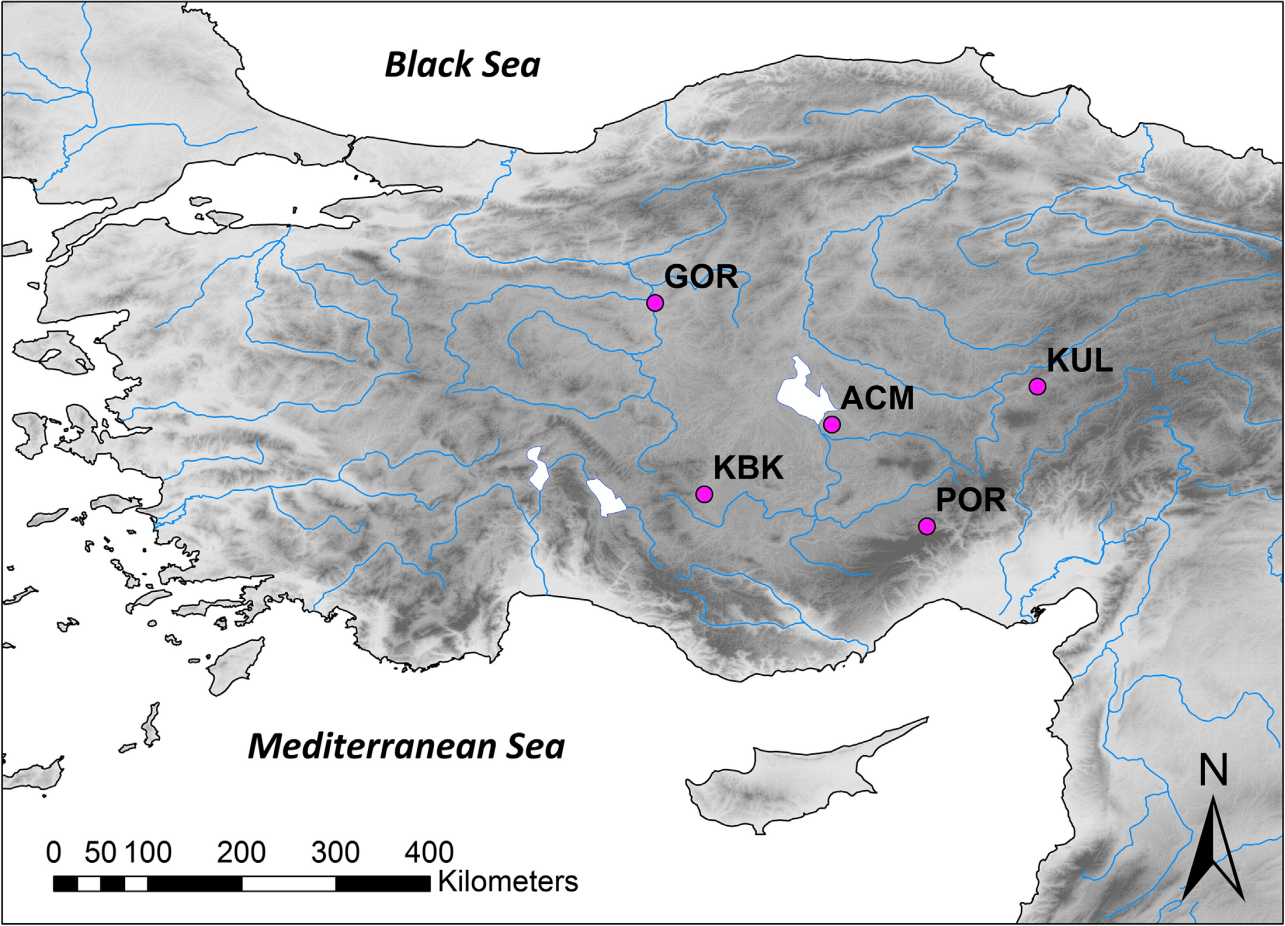
Locations of Anatolian sites with tree-ring chronologies analyzed and discussed in this paper.

Due to the amount and context of preserved wood at the archaeological sites of Kültepe and Acemhöyük in Anatolia (Fig 2), dendrochronological evidence is relevant to this discussion. There has been notable success in building tree-ring chronologies at these and other Anatolian sites [20], and these data sets would be–as has been argued [21–23]–key to resolving the Mesopotamian chronology dilemma *if* the requisites for crossdating between sites were met in all cases. The existing dendrochronological work has been employed as part of the basis of several recent dating proposals by ancient historians (e.g. [5, 9–11, 15]). But, just as recent discoveries and advances with regard to the available textual evidence have transformed understanding as encapsulated in the REL [5], so reappraisal and new work reveals that some of the dendrochronological evidence published until now must also be reconsidered. This critically affects all work in the field published to date which has employed the dendrochronological crossdating among Anatolian sites using the Porsuk chronology (e.g. [5, 9–11, 15, 21–23]).

It has previously been shown that dendrochronological evidence for two MBA sites, Kültepe and Acemhöyük, is relevant and robust [20, 23]. Documents recovered in houses belonging to Assyrian merchants settled at Kültepe (ancient Kaneš) [4] place the transition from Kültepe Lower Town (referred to as the “kārum” in earlier literature) Level II to Ib between REL 138 (latest attested year eponym in Level II) and REL 142 (first attested year eponym in the subsequent Level Ib)–thus REL 138–141 [5]. (It is worth noting that we have only very few texts with dates for REL 142 onwards, and usually only one for each year, thus the REL 138–141 ‘gap’ may change or close with new finds.) At *approximately* the same time a new palace–the so-called Waršama Palace of Mound Level 7 –was constructed on the citadel at the site of its burnt-down predecessor, the so-called Old Palace of Mound Level 8, and is contemporary with Kültepe Lower Town Level Ib [4, 5, 24]. There is wood (*Juniperus* spp.) from the construction of the Waršama Palace with bark preserved, which allows dating of the exact year that the tree was felled, thereby offering a potential date for the palace’s construction within a year or so [23], and a potential tie point with the REL [5].

However, there is a critical gap in the evidence: the documentary record comes from the lower town area, which is entirely discrete from and with no stratigraphic or decisive documentary relationship to the Waršama Palace [4, 5, 24]. A simultaneous destruction and transition from (i) Lower Town Level II to Ib, and (ii) the destruction and transition from the Old Palace to the Waršama Palace has hitherto been maintained as the most likely scenario (e.g. [4]) and employed as a premise in [5]. But this assumption cannot itself form a fundamental link in the evidence chain and needs to be tested.

A second site offers an independent starting point for doing so. A large number of bullae (sealed clay lumps) bearing e.g. the sealings of Šamši-Adad I, king of Upper Mesopotamia, and ruler of Aššur between REL 165 and his death in REL 197 (a reign lasting 33 or 34 years [25]), were found at the Sarıkaya Palace at Acemhöyük in Anatolia (e.g. [5, 26–29]). Numerous *Juniperus* spp. timbers were recovered, some with bark, or with only bark removed (the waney edge), from half a dozen different locations around the building complex. Timbers of a similar age were also recovered from another construction at the site, the Hatıpler Tepesi [20, 23]. For this site, there is a robust chronology of 12 samples (see Figure D in S1 File, S2 Dataset, Table D in S1 File). Most of the relevant bullae with their seal impressions were found stored in Room 6 of the Sarıkaya Palace [27], where two of the 12 tree-ring samples (ACM-48, ACM-50) were collected (see Figure D in S1 File). The final extant tree-ring of ACM-48 is close to bark and dates to the same year as other samples from the building with waney edges or bark (Figure D in S1 File). Thus these timbers should reasonably provide a *terminus post quem* for the deposition of the impressed bullae. Of course, the actual date that the bullae and their sealings were produced is earlier than deposition, but, given their large number (900 bullae and 43 tags from Room 6 alone) [26–29], it is reasonable to assume that such a larger group of heirloom sealings are only ‘old’ by less than a decade. Therefore, it is plausible that the majority of the sealed bullae postdate the construction of the room in which they were stored, and that at least some of these must include the 29 bullae carrying impressions of seals of Šamši-Adad I. The finds of a dozen similarly aged and securely crossdated timbers (some preserving bark or waney edge)–with high inter-sample correlation, implying the timbers were collected from a similar source–along with other tree-ring sequences that crossdate robustly with this site’s chronology (Figure D in S1 File, S2 Dataset, Table D in S1 File) [20, 23] from *different* loci in the Sarıkaya Palace and another nearby complex, make it very unlikely that these are instances of re-used wood–rather these timbers were newly cut wood obtained and used in major building projects. While documentary and archaeological sources from ancient Mesopotamia indicate the rarity, and thus re-use, of timber sometimes brought long distances [30], it must be noted that juniper timbers would have been available from the mountains close to Acemhöyük and Kültepe–consistent with the view they were felled and obtained for these major building projects.

The numerous, rather than occasional, finds of several types of items dating from the reign of Šamši-Adad I and a number of other contemporary historical figures, suggest that these items are not heirlooms, and therefore indicate that the construction and initial use of the Sarıkaya Palace must not post-date the death of Šamši-Adad I and REL 197 [5]–and indeed must pre-date his death by some years given the range of finds. These include no fewer than 29 bullae impressed with five different seals associated with his reign (13 x Ac. CS 1; 13 x Ac. CS 2; 1 x Ac. CS 3 and 4, 2 x Ac. CS 5) [26–29]. The mention of Aššur in the seal legends of Ac. CS 1, 3–5 means that the seals must postdate the conquest of that city around REL 165. Among the bullae from Sarıkaya were also 15 impressions of two seals (9 x Ac. CS 7; 6 x Ac. CS 8) belonging to king Aplahanda of Carchemish (died ca. REL 208); two impressions made by Līter-šarrussu (Ac. CS 6)–a servant of Yahdun-Lim and Šamši-Adad I whose seal was later re-cut and used by another official on a tablet found at Tell Leilan in Syria and dated to REL 202 (contra [27]); and five impressions of the seal of Nagihan/tum (Ac. CS 10), “daughter of Yahdun-Lim, king of Mari and the Sim’alites”, whose father was defeated by Šamši-Adad I around REL 180, but who may have continued to use her seals for years thereafter. While documentary connections can be made between the Sarıkaya Palace and Lower Town Level Ib at Kültepe [29] (corresponding to the period from REL 142 onwards), there is no evidence for any (earlier) Kültepe Lower Town Level II connection, nor is there any sound evidence for documents dating prior to the conquest of Aššur by Šamši-Adad I (REL 165) at the Sarıkaya Palace ([29], contra [27]). The bullae found in the Sarıkaya palace provide a relative date of the structure between REL 165 and the death of Aplahanda (after REL 208), with a cluster of dates for the bullae in the REL 190s.

### Anatolian MBA Dendrochronology and Problems with Previous Work and Dates

**Fig 3:**
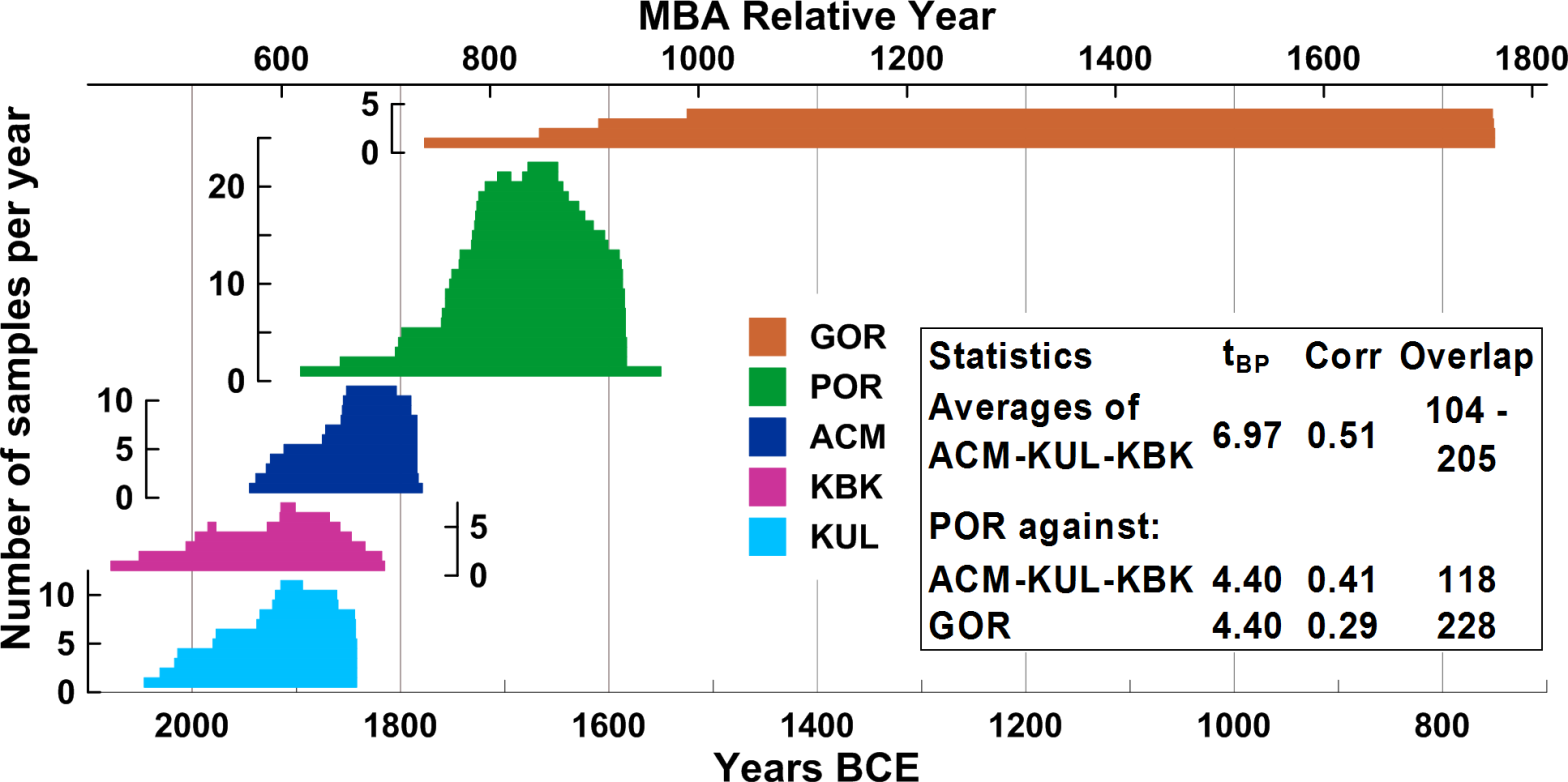
Site tree-ring chronology composition, showing sample numbers/depth and their relative positions according to the previously proposed dendrochronological placements [23] and a summary of the crossdating statistics (t_BP_) from the TSAP software [37] (other crossdating statistics are available in S1 File).

Robust *Juniperus* spp. tree-ring chronologies from three Middle Bronze Age (MBA) sites in Anatolia (Fig 2)–Kültepe (KUL) and Acemhöyük (ACM), plus one other site, Karahöyük Konya (KBK) [20, 23, 31] (and see Section A in S1 File)–do securely crossdate following standard dendrochronological methods and evaluation [32–39], both within and between sites with no doubt as to veracity (see inset in Fig 3 and Figures A, B and Tables C, E in S1 File), and combine into a robust floating 300-year MBA juniper chronology [20, 23, 31]. The Gordion (GOR) *Juniperus* spp. chronology is likewise well-established and near-absolutely placed in time [22, 23, 31, 40, 41]. But the link between the MBA chronology and the Gordion chronology is problematic at best, resulting from an attempt to connect the two via a dendrochronological ‘bridge’, a *Juniperus* spp. chronology from Porsuk (POR) (southeast Anatolia) [21–23, 31, 40, 42]. There are two issues here. First, the part of the chronology linking Gordion and Porsuk contains very low sample depth–with just 1 or 2 trees in the Gordion chronology (Fig 3, Figures A, B in S1 File)–too few for any secure crossdating following standard dendrochronological methods [32–35], which makes the best available match, whether statistical, visual, or both, highly tentative at best (nor is the best available match adequate: inset in Fig 3 [35]). Second, the supposed linkage between the Porsuk chronology and the MBA chronology is also, in dendrochronological terms, no more than tentative due to the overlapping segment of POR containing only 1 or 2 samples, again too low for secure crossdating (Fig 3, see Section A in S1 File, Figures A, B, Tables C, E in S1 File).

Based on this information, there are no valid crossdates between the Porsuk and MBA chronologies, nor between the Porsuk and Gordion chronologies. Thus any previously stated calendar date estimates for the Porsuk, Acemhöyük, Karahöyük or Kültepe timber samples extrapolated from the Gordion chronology are not secure in dendrochronological terms. This calls into question existing work on Mesopotamian chronology which has used such dates as part of assessments or arguments (e.g. [5, 9–12, 15]). Secure absolute dating of these chronologies instead requires additional analysis using independent dating techniques.

### Reassessment of Dendrochronological Dating

In view of these issues, and to resolve the chronological uncertainty, we report work, using tree-ring-^14^C-wiggle-matching [43–45] to (i) independently establish a near-absolute date for the MBA tree-ring chronology from Kültepe, Acemhöyük and Karahöyük; (ii) independently establish the near-absolute date of the Porsuk tree-ring chronology; (iii) re-assess previous dendrochronological crossdates; and (iv) re-assess the relationship of the dendrochronological dates with Mesopotamian history. Our direct date for the Porsuk tree-ring chronology revises the date for this chronology and confirms that Porsuk lacks secure dendrochronological crossdates with both the MBA chronology and the Gordion chronology at the previously proposed placement. We demonstrate that our revised dating scheme for Porsuk and the MBA chronology is compatible only with the so-called Middle or Low-Middle Mesopotamian Chronologies. Further, our work moves the date of a previously reported unusual tree-ring growth anomaly in the Porsuk chronology–tentatively associated with the effects of the Santorini/Thera volcanic eruption [21, 22, 46]–to an earlier period, meaning that this growth-ring anomaly is not associated with the Santorini event.

## Materials and Methods

### Tree-ring samples

The tree-ring samples analyzed and ^14^C-dated in this paper are juniper (*Juniperus* spp.), either *Juniperus excelsa* M.Bieb. or *Juniperus foetidissima* Willd. [47, 48], recovered from the archaeological sites of Acemhöyük, Karahöyük Konya, Kültepe and Porsuk in Turkey (Anatolia) (Fig 2) [20, 23, 31, 42], along with previously published ^14^C data [22, 40] on *Juniperus* spp. material from the archaeological site of Gordion in Turkey [41]. At Acemhöyük and Kültepe the samples associated with the construction derive from the primary building phases. At each site the samples come from more than one locus, and in the case of Acemhöyük from two separate, contemporary, buildings, the Sarıkaya Palace and the Hatıpler Tepesi (including at the former Room 6 where many of the bullae with their seal impressions were found [26, 27]). There are also some samples that indicate later additions/repairs: 22–24 years (based on samples with bark) and at least 61 years (from a sample without bark) later at the Waršama Palace, and at least 8 years (from a sample without bark) later at the Sarıkaya Palace [23]. These suggest minimum lifetimes for the structures as >61 years and >8 years respectively. The crossdated tree-ring series for the samples from each site had been previously assembled into robust site chronologies employing standard dendrochronological methods (e.g. [32–35], see Fig 3, Section A in S1 File, Figures A-G in S1 File, S1 Dataset, S2 Dataset, S3 Dataset, Tables A-E in S1 File) (*note*: Fig 3 shows the chronologies in the original–*now withdrawn*–positions). We confirmed the robustness of each chronology by building core chronologies of each site, removing outliers that are satisfactorily crossdated but muddy the chronology’s “common growth signal”.

There are robust crossdates among the three MBA site chronologies, KUL-KBK-ACM, and they form the basis of the combined MBA juniper chronology. As noted above, the best available linkages of any of the MBA set elements with POR are less than secure, as is GOR with POR (Section A, Tables C, E in S1 File) due to the small sample count in one of the two data sets in each overlap and insignificant or poor statistical correlation (Fig 3, Tables C, E in S1 File).

### ^14^C and Dendrochronology

We carried out a program of tree-ring-^14^C-wiggle-matching on elements of the MBA and Porsuk chronologies, similar to previous work on the Gordion chronology [22, 40], in order to: (i) date directly and precisely the MBA relative chronology, and thus (ii) obtain calendar date estimates specifically on a relative dendrochronological sequence associated with the historical Mesopotamian world via documents linked especially to the reign of Šamši-Adad I and the REL [5]; and (iii) independently test and quantify the correct relationships between the Gordion and Porsuk chronologies, and the Porsuk and MBA chronologies.

**Fig 4:**
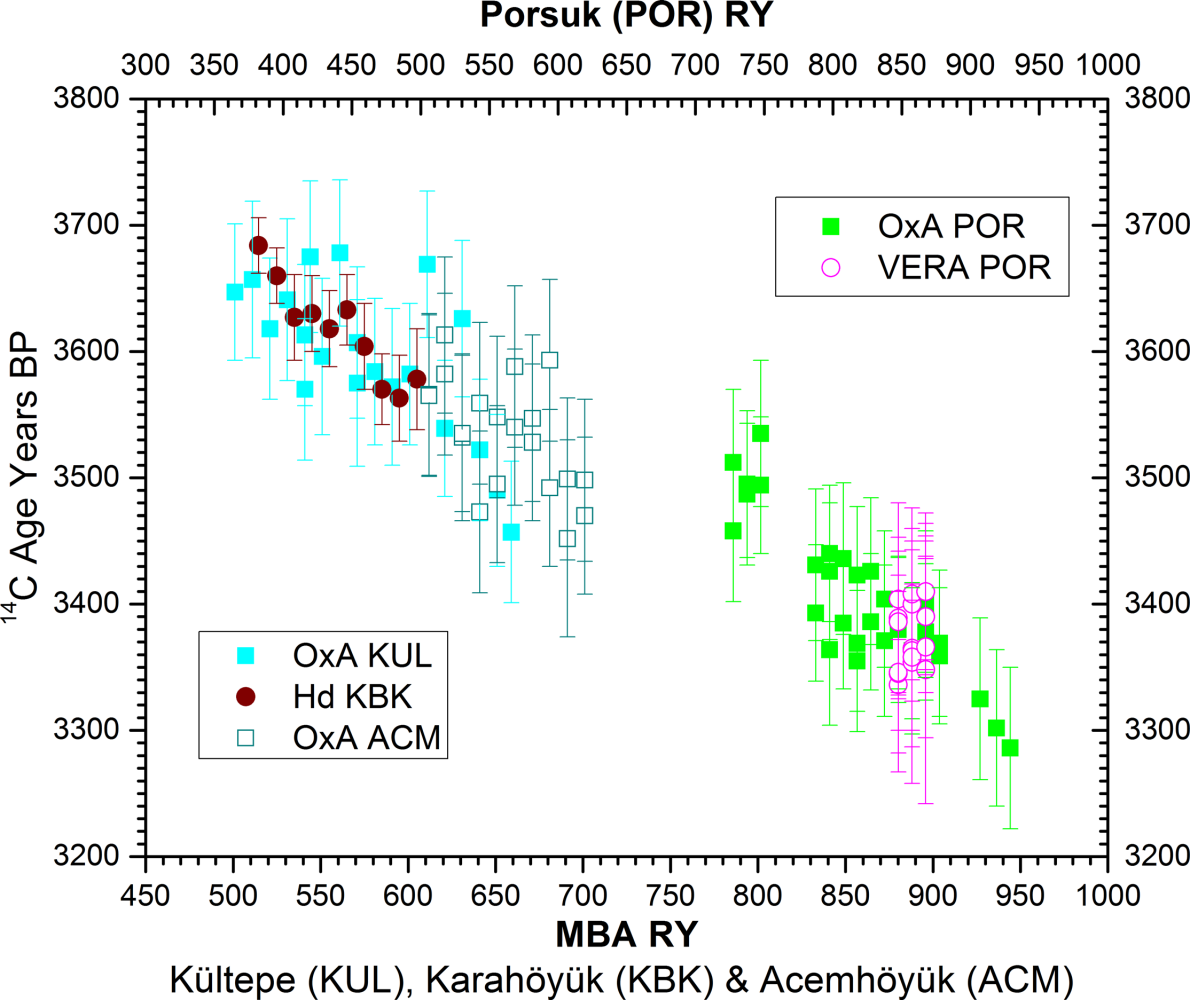
Comparison of the ^14^C data on each set of *Juniperus* spp. tree-rings from each site. Errors bars show 95.4% probability ranges. In all but one case, OxA-30897 & OxA-31521 from MBA relative rings (RY) 677–685, the data on the identical tree-rings are compatible to be combined as weighted averages to give the best available estimates of the appropriate ^14^C age [60]. Dates on the same or similar (site or time interval) tree-rings measured at different laboratories also show good agreement.

Specific sets of juniper tree-ring segments (each typically comprising 9 or 10 tree-rings, or less in a few cases) were dissected with a steel blade under a binocular microscope from the POR, KUL, KBK and ACM tree-ring series with known tree-ring counts for each sample and for the intervals between each sample, in order to form defined time series. These samples were then ^14^C dated (Fig 4, see Section A in S1 File–all new ^14^C data from this project are listed in Table F in S1 File). ^14^C ages are expressed as conventional radiocarbon ages BP (Before Present–AD/CE 1950) using the (Libby) half-life of 5568 years [49]. We summarize sample pretreatment methods employed at each laboratory:

#### (i) Oxford (OxA)

Acid-Base-Acid (ABA) sample pretreatment (lab code: UW), target preparation and Accelerator Mass Spectrometry (AMS) ^14^C dating were performed on the juniper samples at the Oxford Radiocarbon Accelerator Unit following methods described previously [50–53]. Isotopic fractionation has been corrected for employing the δ^13^C values measured on the AMS–the quoted δ^13^C values (Table F in S1 File) were measured independently on a stable isotope mass spectrometer (±0.3‰ relative to VPDB).

#### (ii) Vienna (VERA)

Sample pretreatment, target preparation and AMS ^14^C dating were performed on the juniper samples at the Vienna Environmental Research Accelerator mainly following procedures described previously [50, 54, 55]. Three subsamples of each sample were treated with the standard ABA method used at VERA. In addition, from one of the ABA samples, the humic acids–extracted in the alkaline step–were precipitated and measured to check whether their ^14^C age deviates from that of the fully treated sample (which was obviously not the case) [56]. Further, for comparison, two other subsamples were subjected to Soxhlet extraction with cyclohexane-ethanol 2:1 mixture, ethanol and bi-distilled water in sequence followed either by ABA treatment or by acid–base treatment plus a final bleaching with NaClO_2_. The VERA δ^13^C values in Table F in S1 File were measured with the AMS system on the graphitized sample.

#### (iii) Heidelberg (Hd)

Sample pretreatment and ^14^C dating were performed on the juniper samples at the Heidelberg Radiocarbon Laboratory following methods described previously [22, 40, 57]. The KBK samples were analyzed in 2002 (this gas-counting facility is no longer in operation). The δ^13^C values quoted were measured independently on a stable isotope MS (typical approximate precision 0.4‰). The samples were milled and then pretreated using versions of an AAA sequence [58] with a slightly modified de Vries method (NaOH overnight; HCl, NaOH, and HCl for 1 hour each; all at 80°C). The samples were combusted and the CO_2_ was purified. The samples were measured for (variously) 7 to 12 days at the Heidelberg Radiocarbon Laboratory low-level gas counters [59]. The errors reported in Table F in S1 File comprise the Poisson counting statistics and regression analyzes of background versus coincident count rate (an indicator of barometric pressure changes) and standard versus gas purity.

All but one set of ^14^C measurements on identical tree-rings were compatible with the assumption of representing the same ^14^C age within 95% probability limits, and were considered as weighted average values [60]. This includes three parallel sets of samples on the same tree-rings run at different laboratories (Oxford, OxA and Vienna, VERA), and data on tree-ring samples from different trees of similar relative date run at different laboratories (Heidelberg, Hd and Oxford). (For additional information on known age tests for Oxford, to establish accuracy and precision of data reported, and on inter-laboratory comparisons, see Section B in S1 File, Figure I in S1 File.)

The tree-ring-sequenced ^14^C datasets were then each independently wiggle-matched to the IntCal13 ^14^C calibration dataset [61] with curve resolution set at 1 year using the OxCal 4.2 software system and employing the D_Sequence function [43, 62]. Different models were run, starting with all data, and then excluding any outliers (see [63] and Section C in S1 File). The RScaled outlier model in OxCal was employed as the errors involved in this study are of the ^14^C timescale (and not the tree-ring/calendar timescale), but may reasonably be regarded as potentially somewhat understated in real terms, as evident, for example, in previous high-precision inter-laboratory comparisons [64]–thus the RScaled option seems more appropriate/realistic in a case like ours than the SSimple model which takes the quoted errors on the ^14^C measurements literally [63]. The mid Relative-Year (RY) point of each dated sample was treated as the RY age being dated and fitted to the ^14^C calibration curve (e.g., where there are 1–9 rings in a sample, the RY date of ring 5 is used, or where there are 10 rings in the sample, we use ring 5.5 as the approximation). Tables G-J in S1 File provide the full OxCal model information for Models 3, 6b, 8a and 8b (Table 2)–noting Outliers from Models 1 and 7a and 7b and any comments about the individual dates in the models. Section C in S1 File provides further details. Figures J-N in S1 File show the calculated placements, or details, from various models. We note that the OxCal software implements a comprehensive Bayesian chronological modelling approach [62]; among other things, this permits definition of probability regions and robust outlier detection [62, 63]. Classical least-squares curve fitting approaches achieve very similar best fit loci [22, 43]. To test and confirm this in the present case we considered the Model 7b data via a least squares analysis versus IntCal13 [61] achieving a best fit only 6 years different (older). After removing the 3 data more than 3σ from the mean IntCal13 values, a re-run was only 4 years different (older) than Model 8a (see Section C in S1 File, Figures O, P in S1 File).

**Table 2:** Dendro-^14^C-wiggle-match placements for the Anatolian tree-ring series.

Dating Model	Elements /dates	ring 776 Date BCE		68.2% hpd range	95.4% hpd range	A_model_	A_overall_	Outliers
		*Gordion Scheme Relative Years (RY)*						
		μ±σ	M^+^	Date BCE	Date BCE			
**1. GOR RY776.5–1764**								
	128/128	1738±1	1738	1740–1737	1741–1736	0.8	0.8	25
**2. = 1 minus outliers**								
	103/103	1738±1	1738	1739–1736	1741–1735	123.4	118.4	1
**3. = 2 minus outlier**								
	102/102	1738±1	1738	1740–1737	1741–1735	141.4	133.9	0
***3a*. *= 3 with ΔR 0*, *10 (μ±σ = 6*.*9±2*.*3)***								
		1738±2	1738	1739–1736	1741–1734	247.7	233.2	0
**4. GOR RY776.5–1145.5**								
	50/50	1732±3	1732	1736–1730	1739–1729	10.9	12.4	7
**5. = 4 minus outliers**								
	43/43	1734±2	1734	1737–1732	1737–1726	97.7	98.8	0
**6. POR**								
	18/51	1753±7^#^	1754	1759–1751	1762–1740 (*91*.*4%*), 1736–1730 (*4%*)	92.6	103.1	0
***6a*. *= 6 with ΔR 0*, *10 (μ±σ = -5*.*6±6*.*4)***								
		1754±8	1756	1760–1753	1764–1746 (*89*.*6%*), 1735–1725 (*5*.*8%*)	118.1	125	0
**6b. POR (OxA & VERA separate)**								
		1753±6	1755	1759–1752	1762–1740 (*93*.*2%*), 1736–1732 (*2*.*2%*)	100	107.3	0
**7a. KUL-KBK-ACM = MBA (KUL and ACM samples separate)**								
	39/103	1746±8	1744	1749–1738	1771–1734	4.3	13.1	6
7b. 7a but with KUL+ACM combined, minus 1 sample*								
	34/102	1746±10	1743	1749–1737	1773–1764 (*9*.*8%*), 1757–1773 (*85*.*6%*)	5.5	18.8	5
**8a. = 7a minus outliers**								
	33/90	1744±4	1744	1748–1739	1752–1736	137.2	142.1	0
**8b. = 7b minus outliers**								
	29/90	1745±4	1745	1749–1741	1753–1738	121.3	128.1	0
***8c*. *= 8a with ΔR 0*, *10 (μ±σ = 3*.*4±5*.*7)***								
		1742±5	1741	1746–1736	1752–1732	170.7	167.9	0

Calendar date (BCE) placements for the dendro-^14^C-wiggle-matches of the Gordion, Porsuk and MBA tree-ring chronologies (Fig 3) employing IntCal13 and OxCal [43, 61, 62] expressed in terms of RY776 following the previous chronology based on the Gordion RY sequence and dates published [21–23, 31, 40, 41]. The models consider first all data in each series, and then excluding outliers if present (RScaled outlier model in OxCal [63]) and considering possible ^14^C offsets (ΔR [62]). Typical outputs are shown (see also Figures J-N in S1 File). The OxCal Amodel and Aoverall values should be about ≥60 to indicate satisfactory agreement within the model. Elements are the separate dendro-sequenced analytical units in the models–these may comprise one or more dates; where plural these dates on the identical tree-rings are employed as a weighted average [60]. Note: RY776 is simply an arbitrary choice (but used since it was the reference point for previous Gordion-based dates) to allow comparison of the different chronologies. *If* the previously published crossdates and positions were correct (e.g. [21–23, 31, 40, 41]), then all the chronologies should yield approximately the same calendar age ranges for RY776. *They do not*. The tree-ring series do not all include an RY776 –this year is extrapolated in such cases to enable the comparison in terms of a single year.

+ M = median value

# The probability distribution for the Porsuk series is *not* approximately symmetrical–contrast all other cases–the mode of the distribution is 2 years older than the mean, and 1 year older than the median–at 1755 BCE for Model 6. We use 1755 BCE as the best fit point.

* OxA-30907 for RY607-615 had divergent δ^13^C values recorded by the AMS versus those independently measured on an MS, and so is considered inherently less than entirely reliable and so was excluded in run 7b (but included in run 7a).

**Fig 5:**
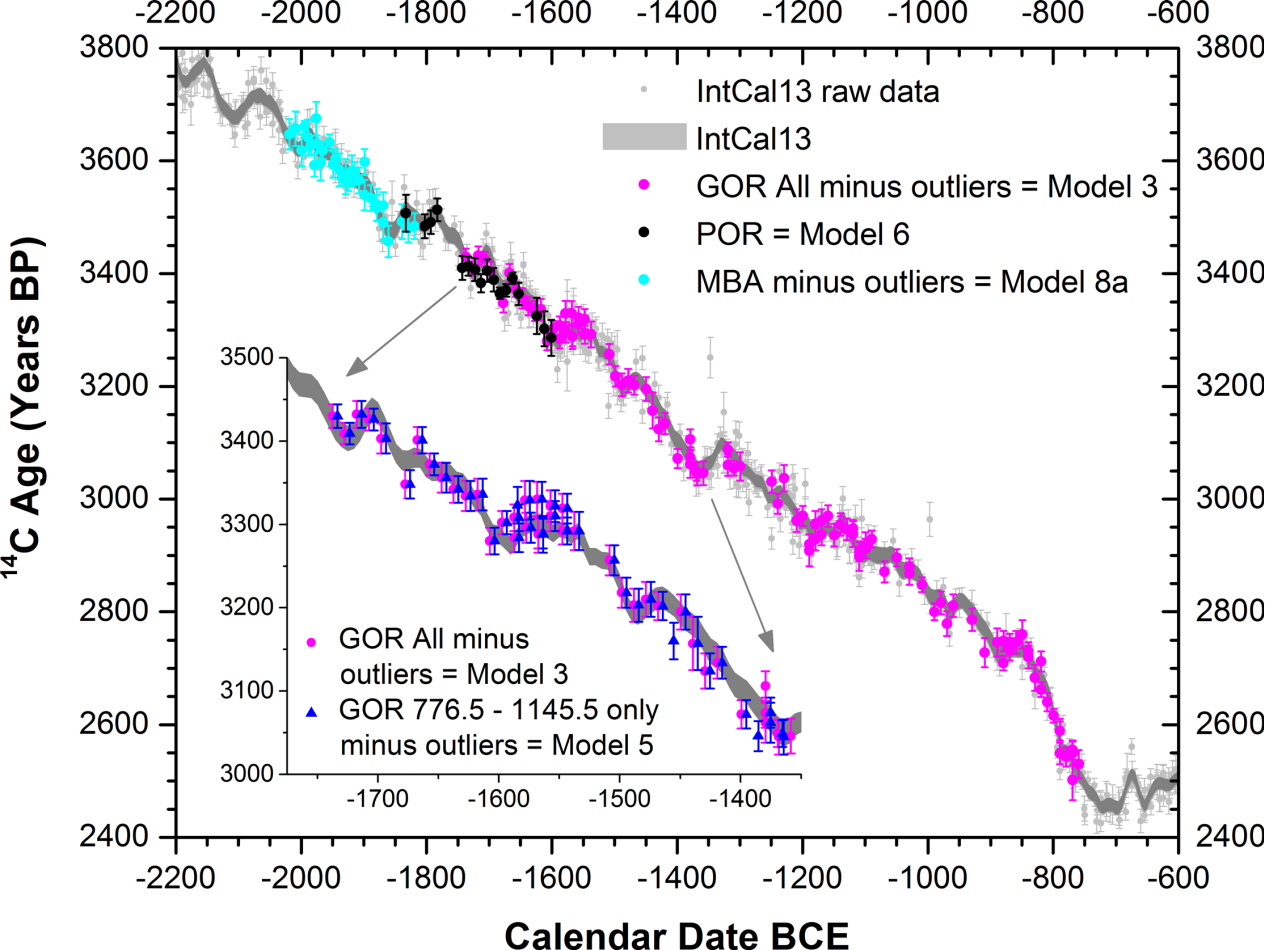
^14^C measurements from the tree-ring time-series excluding outliers as best placed against the IntCal13 ^14^C calibration dataset (modelled and raw data) [61–63]. Plot shows data and placements from Models 3, 6, 8a in Table 2 (see also Figures J-N in S1 File). Inset shows the alternative placement for the Gordion series if only Gordion RY 776.5 to 1145.5 are employed [40], i.e., Model 5 versus Model 3 placement (see Table 2) (see Section C in S1 File).

**Fig 6:**
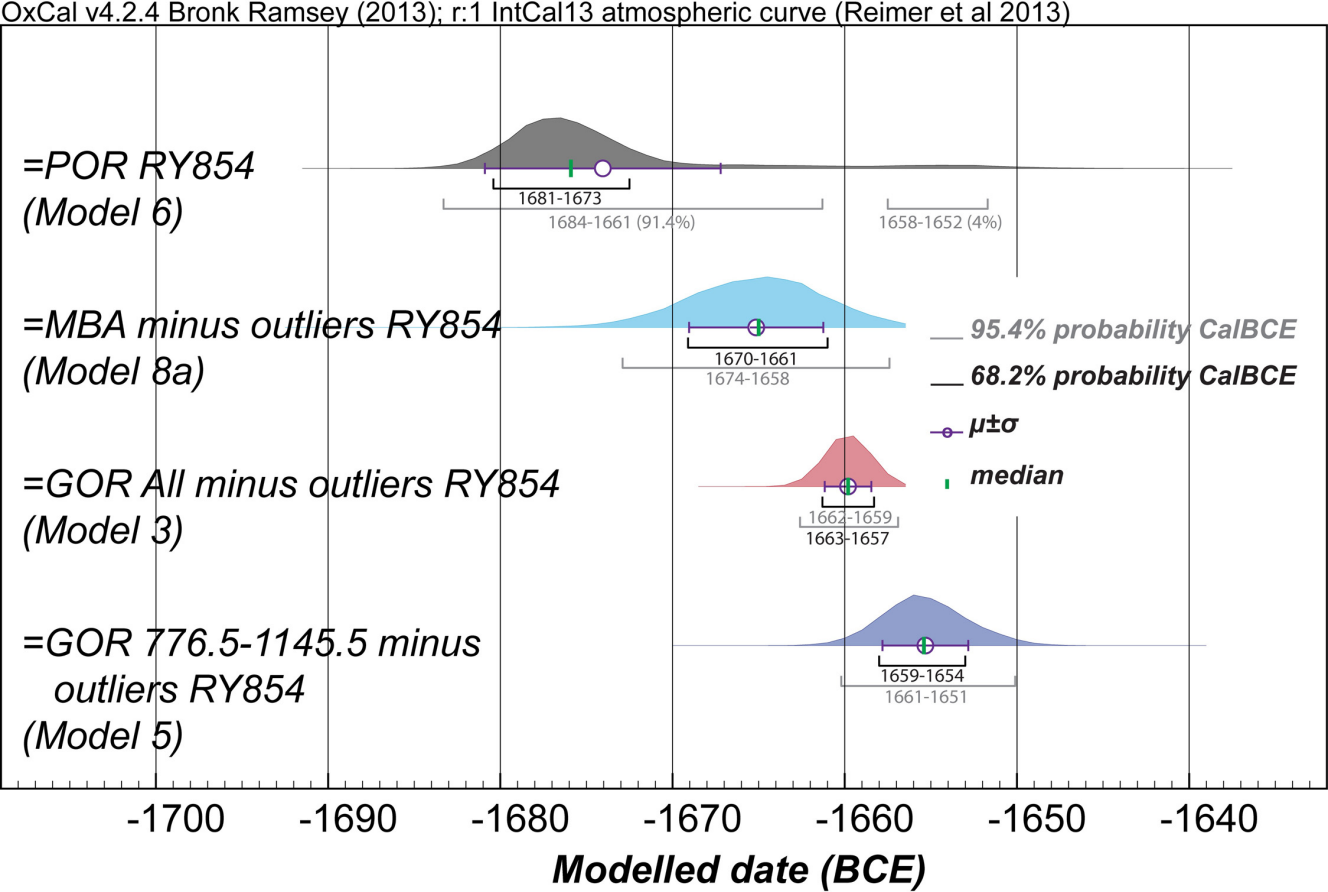
We test the previously published Anatolian dendrochronological placements [21–23, 31, 40] by independently dating the MBA, POR and GOR chronologies and calculating the calendar age range for what should–if the previous placements were correct–be exactly the same date. We choose to test the previously published placements versus the major RY 854 tree-ring growth anomaly observed in the Porsuk chronology (this is an arbitrary choice but of relevance to the supposed Thera/Santorini association [46]). Differences between the dendro-^14^C-wiggle-match placements of the various time-series (see Fig 5) are shown in terms of the overall calculated probability distributions (marginal posterior densities) for the calendar age placement for RY 854 (note: the tree-ring series do not all include an RY854 –this year is extrapolated in such cases to enable the comparison in terms of a single year). The 68.2% and 95.4% highest posterior density (hpd) ranges, along with the μ±σ and the median, are also indicated.

We also considered the relevance of possible regional ^14^C offsets–Anatolia versus the sources of the IntCal13 dataset (southern Germany and Ireland for this time period)–in order to achieve the best and robust calendar placements (Fig 5, Fig 6, Table 2, see also Section C in S1 File). No substantive offset was identified for these timbers across the relevant time period consistent with findings for the second millennium BCE previously reported for central Anatolia from Gordion [40]. We also reanalyzed the previous Gordion series [40] using the newer IntCal13 calibration dataset [61] (replacing IntCal09 as employed in the previous study [40]) (Fig 5) because additional data included in IntCal13 reduce somewhat small discrepancies noted previously with respect to the Gordion dataset [40, 65]. Overall, it is important to highlight that the analyses we report, whether with or without outliers removed, or considering possible small regional ^14^C offsets, all yield very similar calendar date placements for each of the chronologies (GOR, POR and MBA), with mean or median values varying only by 0–3 years: Table 2.

## Results

### Porsuk

**Fig 7:**
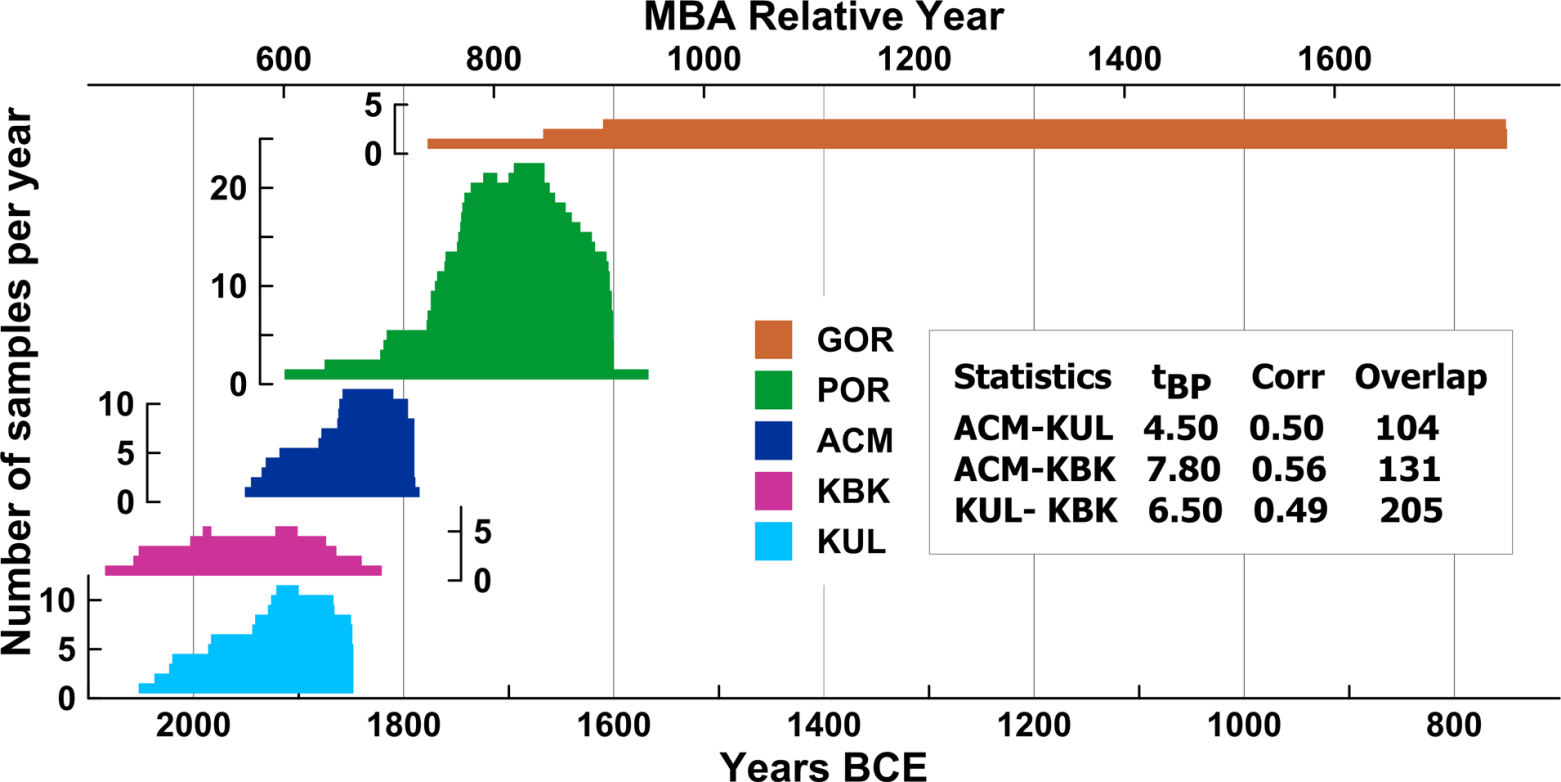
The relative and approximate absolute calendar placements of the MBA, Porsuk and Gordion tree-ring series in Fig 3 revised according to the tree-ring-^14^C dates reported in Table 2 and with no dendrochronological crossdate connection between the Porsuk chronology and either the Gordion or MBA chronologies. Inset: the t_BP_ crossdate values calculated from TSAP [37] for the constituents of the MBA chronology. There is no tree-ring-based link with either Porsuk or Gordion.

The dendro-^14^C-wiggle-match placement calculated independently for the Porsuk chronology can be compared with the result which would be expected if the previously proposed dendrochronological crossdate against the Gordion chronology were correct (Fig 5, Fig 6, Table 2). We find that the Porsuk chronology is placed ~15–24 years older than suggested by the best, but uncertain, dendrochronological crossdate with Gordion–when comparing median or mean values for Models 1–5 versus Models 6, 6a and 6b in Table 2. Therefore, given that the existing Porsuk relative tree-ring date against the Gordion chronology is both a tentative dendrochronological crossdate, and offers only a very marginal position in the respective 95.4% probability ranges, it should be regarded as problematic, and no longer employed. The approximate revised placement of the Porsuk time series in absolute time is shown in Fig 7.

The re-dating of the Porsuk tree-ring chronology has a further consequence. An unusual short-term tree-ring growth anomaly has been noted in the Porsuk chronology, with the hypothesis suggested that this might result from the impact of the large Santorini (Thera) volcanic eruption known from the mid second millennium BCE [21, 22, 46]. Previously, when the date stated for the growth anomaly was ~1650 +4/7 BCE, this seemed at least possible, especially given ice-core based date estimates in the mid-1640s BCE published in the later 1980s-1990s [21, 22, 46]. Subsequent work indicated slightly older tree-ring dates making this possible association more problematic [40, 66]. However, the recognition now that there is no reliable dendrochronological linkage between the near-absolutely placed Gordion chronology and the Porsuk chronology (see above) renders these previous dates for the Porsuk tree-ring growth anomaly invalid. The independent dating of the Porsuk chronology reported here (Figs 5 and 6 and Figure L in S1 File, Table 2) indicates that the Porsuk chronology in fact dates likely some ~15–24 years earlier than the date (now recognized as incorrectly) derived via the Gordion chronology. The independent Porsuk dendro-^14^C wiggle-match places the RY 854 growth anomaly in the Porsuk wood most likely ~1681-1674/1673 BCE (68.2% hpd ranges from Models 6 or 6b –for the 95.4% hpd ranges, see Fig 6) These dates render the Porsuk tree-ring growth anomaly no longer compatible with the plausible date range for the Santorini eruption, which is dated either in the later 17^th^ century BCE from scientific evidence and some archaeological assessments, or in the 16^th^ century BCE according to some other archaeological viewpoints (e.g. [66–70], and see below). Another cause must be given for the Porsuk tree-ring growth anomaly–previous hypotheses (especially [46]) are now unlikely.

### Middle Bronze Age (MBA) Dendrochronology and ^14^C ages

The wiggle-match of the ^14^C ages and the MBA tree-ring chronology (Figs 4–6, Table 2) indicates a precise placement for the MBA chronology likely ~13–16 years earlier than previously suggested [22, 23, 40] on the basis of the withdrawn and non-secure dendrochronological crossdates with the Porsuk chronology (Fig 7). The felling dates for primary construction of the Sarıkaya Palace at Acemhöyük are placed at RY730-731 on the MBA chronology, with some later construction from repairs or alterations. The two years may reflect either the building process of the palace over about 2 years, or stockpiling wood from two years for use. In the pre-modern world there is little evidence that timber was not used more or less immediately (with ‘seasoning’, where mentioned, usually meaning literally after a season, so within 0–1 year of felling) in such roughhewn situations [71, 72] and especially when no lengthy transport is involved–in contrast to some ancient Mesopotamian accounts of timbers shipped long-distances down the Euphrates [30]. Thus the latest year (from samples preserving bark), RY731, should either date construction or end of construction (in the later part of that year) or set a close *terminus post quem* (TPQ) for the end of construction (e.g., year after) and so use of the building, as well as the earliest date for the presence of objects and items such as the clay sealings. We might therefore reasonably regard RY732 as the likely earliest year of actual use of the building. RY732 equates with 1793–1784 BCE (68.2% hpd; the 95.4% hpd is 1797–1781 BCE) following Model 8a in Table 2, see Table 3. The date (bark or waney edge) of the trees used in the primary construction of the Waršama Palace at Kültepe ranges over three years, from RY670-672. Again this may reflect a process (it is an enormous 1 hectare monumental structure [4, 24]) over 3 years, or stockpiling before building. We may again regard either the later part of RY672 as the likely construction date, or end construction date, or the following year, and we can regard the likely earliest use of the palace as not before RY673. RY673 is placed 1851–1842 BCE (68.2% hpd; the 95.4% hpd is 1855–1839 BCE): Table 3.

**Table 3:** Calendar date BCE estimates for the primary construction timbers with bark or waney edge (giving the exact cutting date), and likely earliest building use dates, for the Waršama Palace at Kültepe and the Sarıkaya Palace at Acemhöyük from Model 8a in Table 2.

Model 8a (from Table 2) Relative Years (MBA Dendro)	Primary Construction (PC), *Likely Earliest Use (EU)*	μ±σ	median	68.2% hpd	95.4% hpd
		Calendar Dates BCE			
RY670	Waršama PC	1850±4	1850	1854–1845	1858–1842
RY671	Waršama PC	1849±4	1849	1853–1844	1857–1841
RY672	Waršama PC	1848±4	1848	1852–1843	1856–1840
***RY673***	***Waršama EU***	***1847±4***	***1847***	***1851–1842***	***1855–1839***
RY730	Sarıkaya PC	1791±4	1791	1795–1786	1799–1783
RY731	Sarıkaya PC	1790±4	1790	1794–1785	1798–1782
***RY732***	***Sarıkaya EU***	***1789±4***	***1789***	***1793–1784***	***1797–1781***

### Dendrochronology and Mesopotamian History

#### (i) Acemhöyük

**Fig 8:**
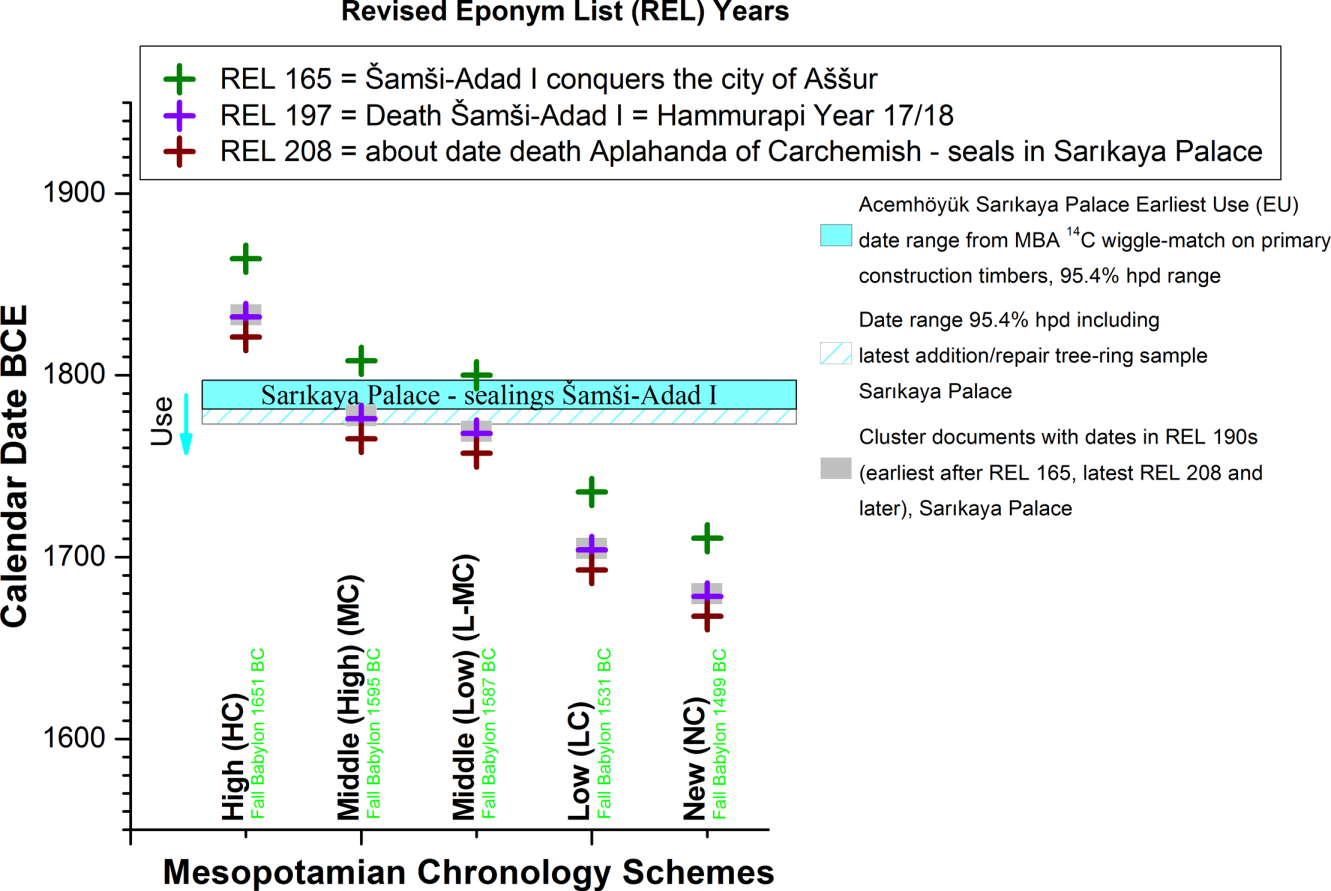
Comparison of the proposed calendar dates BCE from some of the main Mesopotamian Historical Chronologies [5, 9–19, 73] for four key REL dates [5] versus the dendro-^14^C-wigglematch date ranges (95.4% probability range) for the likely Earliest Use (EU) of the Sarıkaya Palace at Acemhöyük [23] as +1 year from latest primary construction timber with bark/waney edge (data from Table 2, Model 8a, Table 3), and including the subsequent dendrochronologically known repairs/additions to the building [23]. The known lifetime of the Sarıkaya Palace runs from the time of Šamši-Adad I (with nothing earlier) through sealings of Aplahanda of Carchemish who died around REL 208. The dates indicated are: (i) REL 165 (Šamši-Adad I conquers Aššur); (ii) REL 197 (death of Šamši-Adad I) [5]; (iii) REL 190s which represent a cluster of dates on documents at the Sarıkaya Palace [29]; and (iv) REL 208. The respective calendar dates BCE for the Fall of Babylon (sacked by the Hittite king Muršili I) according to the five main Mesopotamian Historical Chronologies [5, 9–12, 15–19, 73] are given in green text also (note: the chronology of Mebert [19], 8-years later than the Low Chronology, is not shown).

We may compare the placement of the MBA tree-ring series against the date ranges previously estimated for Mesopotamian chronology based on textual, astronomical and archaeological information as they intersect together in the construction date and assemblage of the Sarıkaya Palace at Acemhöyük (Fig 8). It is evident that only some variation of the Middle Chronology is compatible with the tightly constrained data. Under the High Chronology Šamši-Adad I would be dead four decades before the Sarıkaya Palace was even constructed, which is incompatible with his numerous documentary links with the building (similarly Aplahanda of Carchemish, whose seals are also present and among the later dated material at Sarıkaya, is dead three decades before the building is built). Thus the High Chronology (e.g. [17, 73]) may be ruled out.

The ~13–16 years older shift from our results critically resolves a problem with the (now withdrawn) previous dendrochronological dating. Although this previous date favored the Middle Chronology, it was problematic as it left the construction of the Sarıkaya Palace at Acemhöyük (then given as 1774 +4/-7 BCE [22, 23]) occurring more or less when Šamši-Adad I died (REL197 = 1776 BCE on the Middle Chronology–and not long before Šamši-Adad I’s death on the Low Middle Chronology). And yet there are numerous sealings of Šamši-Adad I in the Sarıkaya Palace [26–29] suggesting, first, that they are unlikely all heirlooms (or a secondary deposit), and, second, that the palace must have existed for at least several years if not a decade or few decades before his death [5, 29]. The new latest primary construction date (RY731) of 1794–1785 BCE (68.2% hpd: Table 3), and removal of the previous Gordion-Porsuk-Acemhöyük dendrochronological linkage, resolves this issue.

#### (ii) Kültepe

**Fig 9:**
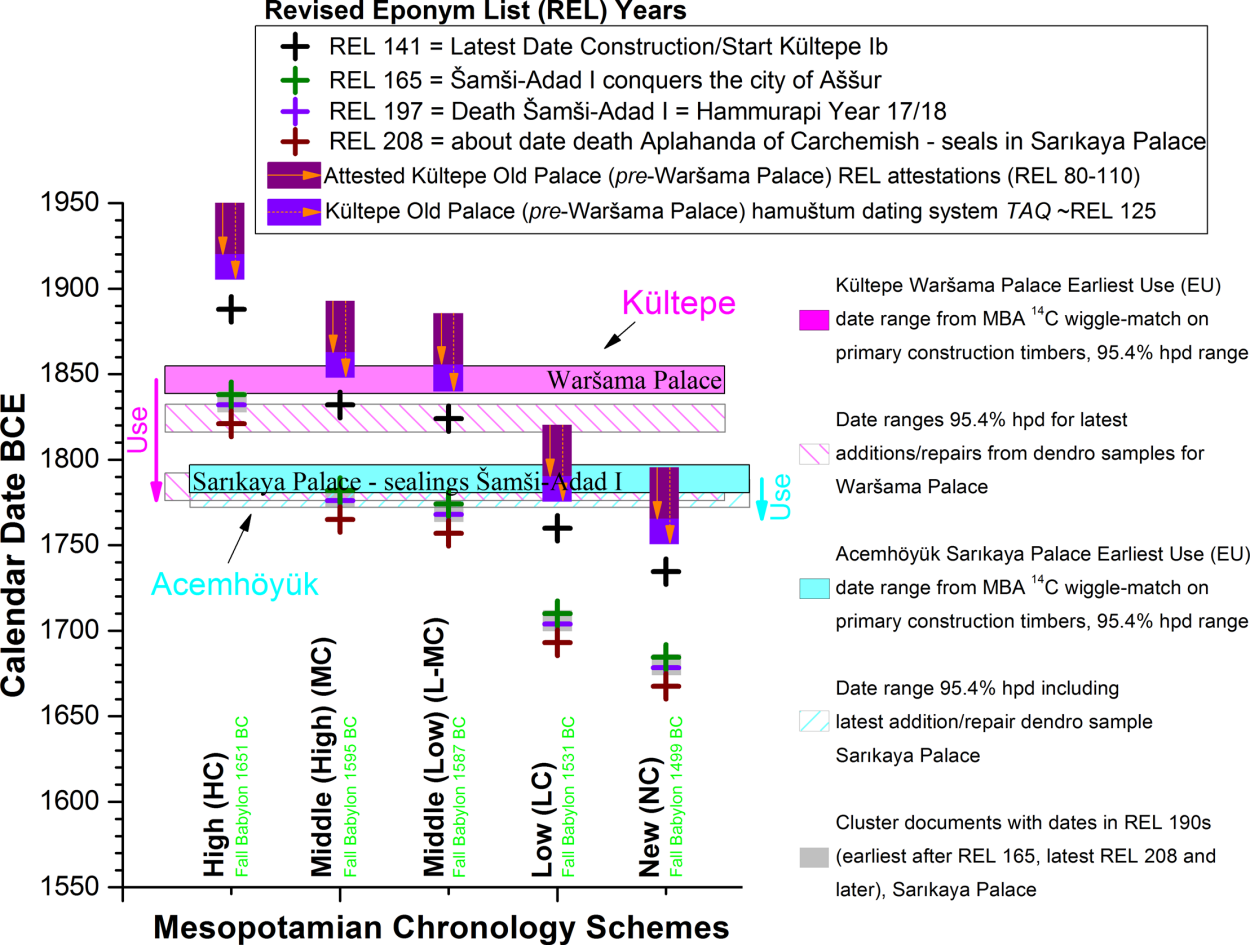
Fig 8 modified by adding the Earliest Use (EU) date range (95.4% probability) and repairs/additions for the Waršama Palace at Kültepe, and adding the calendar date (BCE) for REL 141 (last year before Kültepe Lower Town Level Ib is attested in use) [5] on the basis of some of the main Mesopotamian Historical Chronologies [5, 9–12, 15–19] (Note: the chronology of Mebert [19], 8-years later than the Low Chronology, is not shown). The attested REL dates (80–110) or likely approximate *terminus ante quem* (TAQ) REL date (~REL 125) from Old Palace contexts prior to the construction of the Waršama Palace ([5] at p.31) are also indicated.

The construction of the Waršama Palace at Kültepe (latest primary construction date RY672 in terms of the MBA tree-ring chronology) is placed at 1852–1843 BCE (68.2% hpd: Table 3). Fig 9 modifies Fig 8 by adding the Kültepe evidence and some associated historical dating criteria. It is evident that the construction of the Waršama Palace (or at least the building of the northwest area of this 1 hectare monumental complex [4, 24]–the location of the sampled wood) occurred at least a few years before the beginning of the Lower Town Level Ib. This seems to be a minimum of 8 years earlier and likely ~16 years earlier if we compare the tree-ring ^14^C wiggle-match placement of the MBA tree-ring series, Tables 2 and 3, versus the Middle Chronology (considering the 95.4% probability range and its latest date and then mid-point), or alternatively at least 16 and likely ~24 years earlier if the comparison is made versus the Low-Middle Chronology on the same basis. This importantly questions the long-held but unsubstantiated assumption that the destruction/transition between Lower Town Levels II and Ib equates with the destruction of the Old Palace and building of the new Waršama Palace (as accepted or used by e.g. [4, 5, 24, 74]).

Nonetheless, the assumption was almost approximately valid and our new information is consistent with the evidence to hand. In particular: some documentary materials of Lower Town Level II, which date within the range REL 80–110, or perhaps even as late as a *terminus ante quem* (TAQ) of about REL 125 in one case, were found stratified beneath or in prior fill/debris underneath the subsequent Waršama Palace ([5] at p.31). The placement of the Waršama Palace primary construction (RY672) according to the dendro-^14^C evidence would correspond to about REL 125 (±8 years at 95.4% probability)–this seems a remarkable coincidence since the most recent documentary evidence (a loan document: Kt z/t 17) from an Old Palace context prior to the construction of the Waršama Palace employs the Assyrian week-eponym (hamuštum) dating system which appears to have gone out of use around REL 125 ([5] at p.31). Sometime around or just after REL 125 may therefore offer a good date for the construction of the Waršama Palace. The conflagration that destroyed the Lower Town Level II settlement then occurred slightly later (e.g. ~13–16 or so years later). This offers a scenario compatible with the documentary evidence presently available from the subsequent Lower Town Level Ib [4–5, 7], which has texts containing dates from REL 141 onwards, including the reign of Šamši-Adad I placed REL 165–197.

If we consider Figs 8 and 9, the notable coincidence of consonant scenarios based on the integrated dendrochronological and ^14^C analysis of multiple timbers from monumental constructions at two sites (over 200km apart) demonstrates that the chronology identified reflects the correct historical timeframe and that our findings are not some accident caused by one or two re-used timbers or some other unusual situation affecting one context or even one site. The Middle Chronology offers the best fit between the Old Palace/Lower Town Level II evidence and the construction of the subsequent Waršama Palace, whereas the Low-Middle Chronology only just fits (Fig 9). The Middle Chronology also minimizes the gap between the start of Lower Town Level Ib and the Earliest Use (EU) of the Waršama Palace to likely as little as ~8–24 years (whole 95.4% probability range), whereas it is likely ~16–33 years with the Low-Middle Chronology (whole 95.4% probability range). This is not decisive, but the Middle Chronology allows the best compromise of all the pre-existing archaeological-textual assumptions with the new dendro-^14^C dating framework.

We can also determine that the Low or New Chronology scenarios offer poor or even contradictory fits with the data when we examine Figs 8 and 9 (this observation therefore includes the Mebert chronology [19], 8 years later than the Low Chronology). In particular, the Low and New Chronologies would place REL associations (REL 80–110) attested in Old Palace contexts at Kültepe as dating respectively some 2 to 6, or 4 to 9, decades *after* the Waršama Palace had been built. This is impossible. Thus we may rule out the Low and New Chronologies as implausible. This leaves as plausible either the Middle Chronology or the slightly later Low-Middle Chronology. Our findings directly and independently support some recent analyses of the textual and astronomical records arguing for the Middle Chronology or Low-Middle Chronology as the only coherent solution(s) for Old Babylonian chronology and history [5, 9–11, 15].

As alluded to, there are several possible ways of linking the Kültepe relative (historical) REL sequence to the absolute chronology provided by the new tree-ring-^14^C dating framework. There are also some issues with past assumptions. Despite a long-lived assumption, the archaeology from the site of Kültepe itself has produced no evidence that proves the simultaneity of the conflagrations that destroyed the Lower Town Level II (REL 138–141) and the Old Palace in Mound Level 8 (before the last primary construction date of the subsequent Waršama Palace at 1852–1843 BCE at 68% probability: Table 3). The revised dendrochronological dates for the felling of the timber used in the construction of the Waršama Palace proposed here would require either that the two fire events were separate, contrary to what has generally been assumed, or else it would push back the traditional Middle Chronology by about a decade and a half. For various reasons, the first solution appears more plausible. The already mentioned fact that the sealed envelope Kt z/t 17 (found *in situ* in an Old Palace context sealed by the later Waršama Palace [24] at p.103 and pl.87) presumably has to date to the period prior to REL 125, since it makes use of the hamuštum-system of dating that was abandoned during the latest phase of the Lower Town Level II period [5], is circumstantial evidence in favor of doing so. If the contemporaneity of the fires is retained the envelope would have been ~15 years old by the time it was deposited on the floor of the Old Palace. This is by no means impossible, but it is preferable that the text was drawn up relatively close in time to the fire that destroyed the building in which it was found. With the shift in dendrochronological dates that we report, this evidence now fits comfortably under the Middle Chronology or (a little less) perfectly under the Low-Middle Chronology (Fig 9)–but does not fit under the High, Low or New Chronology scenarios.

The marked decrease in the number of Assyrian dated texts [5] around REL 125 at Kültepe would also follow the new date for the conflagration better, and can be argued to reflect a reduction in commercial activity following the destruction and then rebuilding of the central economic institution in the city. The decrease is particularly evident when taking into consideration the fact that the late period of the Lower Town Level II shows signs of a decreased degree of trade in general–itself probably caused by a wider systemic collapse after REL 110 [5]. Yet, the handful of archives from Kültepe that live past this collapse and continue straight on to the end of the Lower Town Level II prove that the lower town survived until REL 138. More importantly, by separating the two fires, we retain the tie between the REL sequence and the astronomical data (eclipses, Venus tablets), intercalations [5, 8–11, 15] and even potentially the suggested link between a major volcanic dust veil and several northern hemisphere tree-ring growth anomalies 1628–1627 BCE and poor atmospheric observation conditions as evident in Mesopotamian records [10, 75]. Finally, by dissociating the two conflagrations, we gain the necessary time for the deposit of the numerous Šamši-Adad I bullae at Sarıkaya (previously something of a problem), but not enough time to render any of the later chronologies (Low Chronology, New Chronology) plausible (Fig 9).

## Discussion and Conclusions

Our work provides, independent of other assumptions, absolute dating evidence for Mesopotamian chronology in the earlier second millennium BCE from the wiggle-matching of time-series of tree-ring ^14^C dates from individual MBA site chronologies. In particular, we provide direct high-precision date estimates for the primary-construction timbers at Kültepe and Acemhöyük which can be related closely to Mesopotamian chronology and history. These direct age estimates replace previously published dates and are not revisions, since the previous dates were established using what has been shown to be an unsubstantiated dendrochronological bridge between the near-absolutely placed Gordion chronology and the MBA chronology (including Kültepe and Acemhöyük) via the Porsuk chronology. The (for now) separate Porsuk chronology has itself been approximately re-dated and shifted to a ~15–24 years older date placement on the basis of its own independent tree-ring-^14^C-wiggle-match.

Conveniently, the sound new dates we report for the MBA chronology are only ~16 years different (older) than those previously suggested. Hence, although previous arguments using the now replaced tree-ring-based dates (e.g. [5, 9–11, 15]) are inherently invalid in this strict respect, it turns out that the new, robust, evidence nonetheless finds the same Middle or Low-Middle Mesopotamian Chronology solutions are most likely but on a more rigorous basis. Thus, in line with recent text discoveries and analysis and astronomical study [5, 15], we find that only the Middle Chronology or the Low-Middle Chronology (or a chronology very close to these) fits with the new dendro-^14^C dated constraints from the site of Acemhöyük, and also simultaneously creates a plausible historical linkage for the approximately associated dendrochronological-^14^C and text evidence from Kültepe. Contrary to claims that it should be dismissed (e.g. [18, 19, 76]), the Middle or Low-Middle Chronology can henceforth be regarded in approximate terms–with a robust dendro-^14^C anchor–as the accurate timeframe for Mesopotamian history. To express this new resolution in calendar years, the death of Šamši-Adad I (REL 197) may be placed ~1776 BCE or ~1768 BCE, removing previous uncertainty levels of +56/64 calendar years (to the High Chronology) and -64/88 calendar years (to the Low or New Chronologies).

A decision between the Middle and Low-Middle Chronology largely hinges on the astronomical evidence, especially the record in the Mari Eponym Chronicle of what is interpreted as a solar eclipse placed about REL 127, the year after the birth of Šamši-Adad I [5, 9, 10, 15]–though there is some room for debate as the relevant text is not complete [5, 9]. The new dendro-^14^C dates require rethinking of recent analyses, which made assumptions based on the now incorrect previous dendrochronological dates (e.g. [5, 9, 10, 15]). However, in sum, the situation remains similar–assuming we retain the approximate (within about 0–1 year) link between the birth of Šamši-Adad I in REL 126 and an eclipse in REL 127. There is a partial eclipse in 1845 BCE at sunset (hence likely visible) [10], which is within 1 year of the Middle Chronology date for REL 127, and a slightly more conspicuous partial eclipse in 1838 BCE which matches exactly with the Low-Middle chronology date for REL 127 [10]–whereas the total eclipse of 1833 BCE [77] appears too late unless there are substantial unknown errors in the REL sequence. Earlier eclipses, such as in 1859 BCE [9], are too early, unless substantial reconsideration of the standard textual interpretation is considered. Thus both the Middle and Low-Middle Chronology have suitable eclipse candidates within the approximate precision of the available textual evidence [5, 9], but the 1838 BCE eclipse offers a slightly better (more conspicuous) case [10].

If the poor atmospheric observation conditions inferred from the Venus tablet data imply a volcanic dust veil episode in Ammisaduqa years 12–13 [75], then this would offer a convenient match under the Low-Middle chronology with the years 1627–1626 BCE, a period when (from 1628–1626 BCE) a range of tree-ring evidence around the northern hemisphere indicates unusual growth anomalies plausibly associated with the cooling effects of a major volcanic eruption (e.g. [78–81]). The identity of the volcano is not key to the present discussion. Nonetheless, we may note that the enormous Minoan eruption of the Santorini/Thera volcano is often linked with this evidence. Recent work highlights two important developments in this regard. First, the Santorini/Thera eruption was even larger than previously thought [82], and so is even more likely responsible for a major signal in proxy records and a notable atmospheric dust veil. Second, recent work comparing tree-ring and ice-core records over the past 2500 years has indicated age inflation in the GICC05 time scale–as relevant to the Dye-3, GRIP and NGRIP ice-cores–by up to ~11 years [83]. This implies the likely need for a recalibration of the chronology of ice-core evidence for second millennium BCE volcanism from the Dye-3/GRIP/NGRIP records, and so also the date of the major volcanic signal previously dated in the mid-1640s BCE (and associated by some with Santorini/Thera [84–86]). Given the scale of age inflation identified over the most recent 2500 years [83], it would seem likely to assume inflation of ~15–20 years by the mid second millennium BCE, and so the major volcanic eruption signal previously placed in the 1640s BCE might move instead to the ~1620s BCE. This is potentially in line with other observations suggesting the need for a ~20 year reduction in ages for these records around the 17^th^ century BCE, based on comparison of two solar activity proxy records (which should therefore be similar): (i) the ^10^Be record derived from the Greenland ice-cores versus (ii) the Δ^14^C record from known-age trees [87], and similar observations indicating inflated ages for the GISP2 ice-core record which could also make it potentially consonant [88]. Since we may now remove the Porsuk tree-ring growth anomaly from this discussion (see above), all the scientific dating evidence could therefore indicate a likely date for the Santorini/Thera eruption in the later 17^th^ century BCE potentially compatible with the 1628–1626 BCE range (e.g. [66, 68, 69, 78–81, 89–92]). If so, this would provide a relatively proximate, very large explosive volcanic eruption whose dust veil would have travelled (eastwards) directly over Mesopotamia and which could well best explain the unusual atmospheric visual extinction conditions observed in Ammisaduqa years 12–13 [10, 75].

Therefore, although our evidence does not allow a firm decision between the Middle and Low-Middle Chronologies–which are only 8 calendar years apart–we observe that the Low-Middle Chronology offers perhaps the best overall accommodation and compromise among all the evidence from both the dendro-^14^C side and the text-archaeology-astronomy side. The scale of plausible error is now very small and (even allowing for the competing Middle Chronology) less than 10 years in total. Our precise dendro-^14^C-based timescale enables a robust historical synthesis of Ancient Near Eastern civilization in the earlier second millennium BCE; in particular, it facilitates comparison with the coherent historical and ^14^C-based timescale recently available from Egypt [50].

As one example of the wider significance of our findings, we may observe now that chronological and historical scenarios for the Ancient Near East or East Mediterranean regions built around arguments claiming support from, or the need for, the Mesopotamian Low Chronology from other debatable interpretative archaeological and historical connections–such as the ‘low’ chronology applied to the Middle Bronze Age site of Hazor in the southern Levant, or to the Second Intermediate Period archaeological strata at the site of Tell el-Dab‘a in the Nile Delta (e.g. [93, 94])–are invalid and must be revised (as also indicated by critical examination of both the relevant archaeological contexts and ^14^C evidence [69, 95, 96]). Indeed, in the case of the controversy over the dating of the East Mediterranean super-site of Tell el-Dab‘a (e.g. [97] at pp. 383–386), it is noticeable that application of the Middle or Low-Middle Chronology dating could yield dates for the site compatible both with its detailed radiocarbon chronology [98]–which has been rejected by the excavator of the site for no good reason [94]–and other evidence indicating the need for an earlier timeframe for this period and site [66, 68, 69, 95, 96]. In turn, a different history for the east Mediterranean region is implied (e.g. [66, 96, 99]). The direct, near-absolute, dendro-^14^C timeframe for the sites of Kültepe and Acemhöyük reported here thus provides a much needed robust basis for the synchronization of the civilizations of the Ancient Near East and East Mediterranean in the earlier second millennium BCE. It replaces both ambiguity and debate, and thus scope for divergent views, and arguments built on a previous but flawed tree-ring assessment.

## Supporting Information

S1 Dataset Crossdating grid showing crossdating statistics for the samples from Porsuk (POR) employed in this study. *t*_BP_ = Baillie-Pilcher *t*-value (calculated in TSAP [37]); *t*_BP_*-*values >3.5 (to 6) indicate possible matches [35], but experience shows that robust crossdates often require values >6 [35] and that all possible placements must be visually examined in order accurately to crossdate two data sets, because such indicative statistical tests do not necessarily indicate a valid crossdate. GLK = Gleichläufigkeit (trend coefficient) values [33, Reference B in S1 File] and their associated *p*-values, also calculated in TSAP [37]. For the GLK p-values: *** = p<0.001, ** = p <0.01 and * = p <0.05 [37]. The methodological corrections proposed recently [Reference C in S1 File] have been applied here. *n =* years overlap between pairs of sequences.
(XLSX)

S2 Dataset Crossdating grid for the samples from Acemhöyük (ACM) employed in this study. See caption to S1 Dataset for other details.
(XLSX)

S3 Dataset Crossdating grid for the samples from Kültepe (KUL) employed in this study. See caption to S1 Dataset for other details.
(XLSX)

S1 File Supporting Information. Section A: Tree-ring samples, crossdating and evaluation (further information) including Figures A-H, Tables A-F; Section B: ^14^C known age checks (Oxford) and laboratory inter-comparisons, including Figure I; Section C: Chronological modelling and analysis–further details, including Figures J-P, Tables G-J; Supporting Information References (not in main text: References A-G).
(PDF)
