# Supplementary material for: Integrated Tree-Ring-Radiocarbon High-Resolution Timeframe to Resolve Earlier Second Millennium BCE Mesopotamian Chronology
Source: PLoS One. 2016 Jul 13;11(7):e0157144. doi: 10.1371/journal.pone.0157144 (PMC4943651; doi:10.1371/journal.pone.0157144)
Supplement: S1 File — Section A: Tree-ring samples, crossdating and evaluation (further information) including Figures A-H, Tables A-F; Section B: 14C known age checks (Oxford) and laboratory inter-comparisons, including Figure I; Section C: Chronological modelling and analysis–further details, including Figures J-P, Tables G-J; Supporting Information References (not in main text: References A-G). (PDF) [file pone.0157144.s004.pdf]

Supporting Information (File S1)

Integrated Tree-Ring-Radiocarbon High-Resolution Timeframe to Resolve Earlier Second Millennium BCE Mesopotamian Chronology

Sturt W. Manning, Carol B. Griggs, Brita Lorentzen, Gojko Barjamovic,
Christopher Bronk Ramsey, Bernd Kromer & Eva Maria Wild

Section A: Tree-ring samples, crossdating and evaluation (further information) 2
Figs A-H, Tables A-F (see also S1 Dataset, S2 Dataset, S3 Dataset)

Section B: ^{14}C known age checks (Oxford) and laboratory inter-comparisons 19
Fig I

Section C: Chronological modelling and analysis – further details 21
Figs J-P, Tables G-I

Supporting Information References A-G (not in main text) 42

A. Tree-ring samples, crossdating and evaluation (further information)

The tree-ring samples dendrochronologically analyzed and ^{14}C-dated in this paper (see main text for methods) are *Juniperus* spp. (either *Juniperus excelsa* M.Bieb. or *Juniperus foetidissima* Willd.). Figure A in S1 File shows the overall MBA and Porsuk tree-ring chronologies according to the *previous* published positions, which assume a crossdate between the Porsuk and Gordion and Porsuk and MBA chronologies [23] (as in Fig 3, but also indicating some additional crossdated samples not employed in our chronology building analyses based on the most robust series). Figure B in S1 File shows the same information *but* with no assumption of a crossdate between either the Porsuk and Gordion chronologies or between the Porsuk and MBA chronologies as indicated by the re-examination in this paper, and with the approximate calendar placements from the dendro-^{14}C results reported in Table 2. Figures C-G in S1 File show the individual site chronologies, samples, and some additional details. The specific juniper species (*J. excelsa* or *J. foetidissima*) is only known in some cases. The wood anatomy of these two species is very similar [47, 48, Reference A in S1 File], so distinguishing these taxa is not always possible, especially when (as is the case for the vast majority of the samples here), the samples are carbonized and the wood's cellular structure subjected to deterioration. However, the two juniper species frequently co-occur or grow in similar ecological zones in Anatolia, and both modern juniper samples of these two species and historical/archaeological materials (including samples from Gordion) in which the exact species could be determined show strong dendrochronological heteroconnections (that is: crossdating across species) [41]. Thus we do not attempt to distinguish juniper taxa for this study.

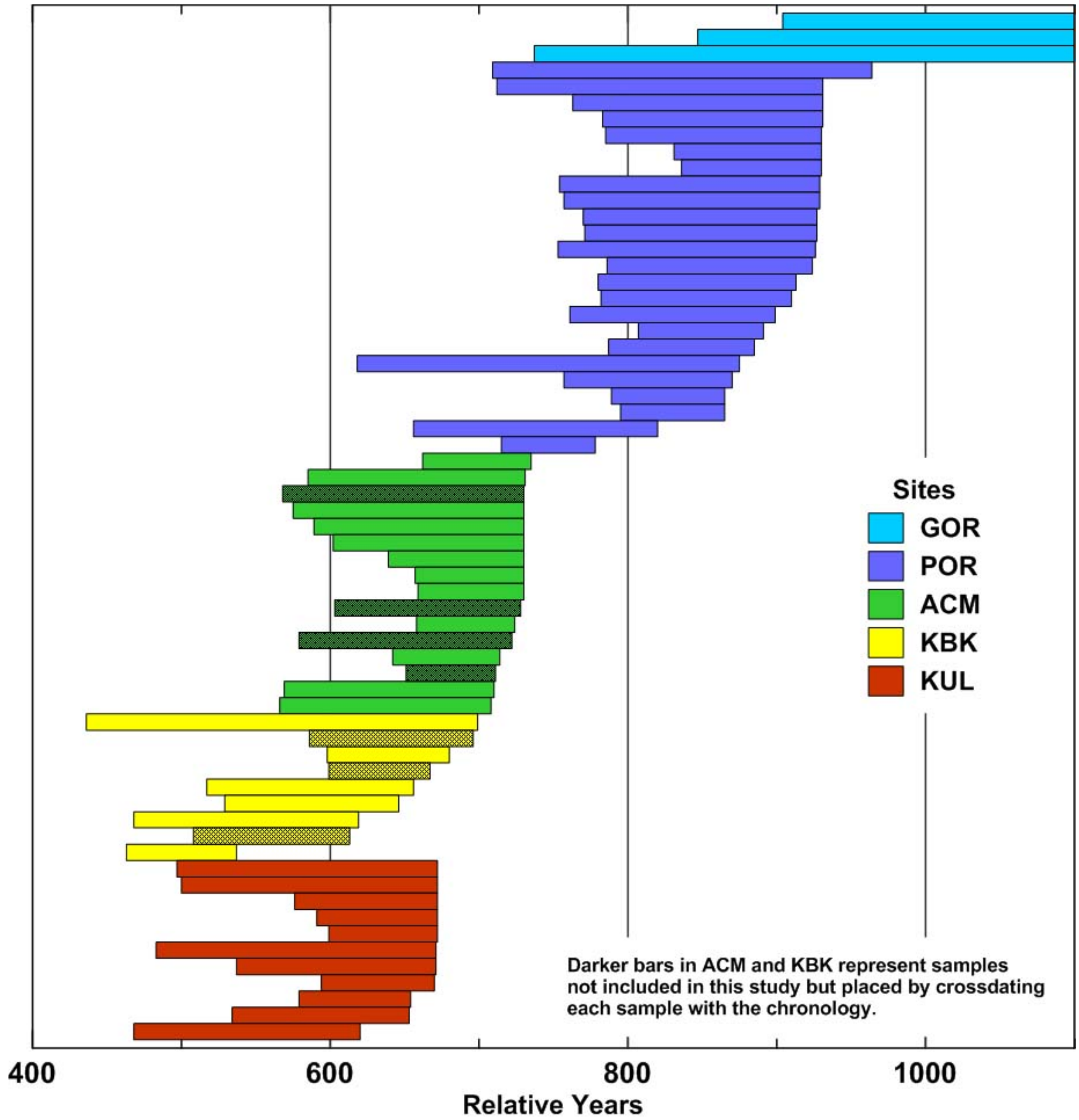

Fig A. The separate elements which comprise the selected MBA (KUL-KBK-ACM), and Porsuk (POR) tree-ring chronologies, and the three Gordion (GOR) elements that overlap in approximate temporal terms with Porsuk (as Fig 3). These tree-ring samples (elements) are shown here placed *as previously reported* [23] – the work reported in this paper *changes* the placements for samples from POR and for the MBA chronology (see Fig 7 and Figure B in S1 File). Some additional samples in the ACM and KBK chronologies are also shown (dark shading) which were not employed in the crossdating analyses. For the individual site chronologies, and for identification of the individual samples in each chronology, see Figures C-G in S1 File, S1 Dataset, S2 Dataset, S3 Dataset, Tables A-C in S1 File.

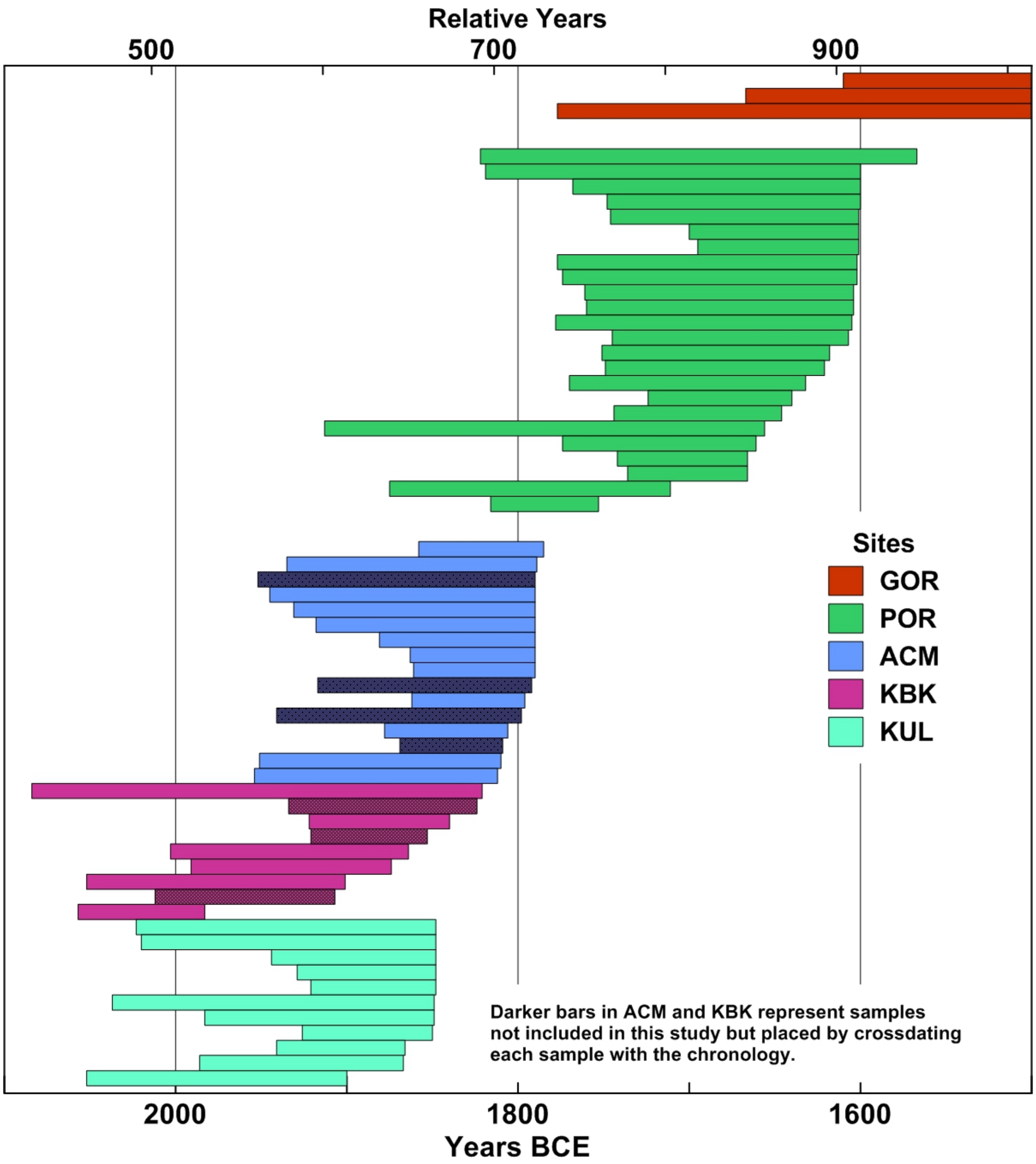

Fig B. Data in Figure A in S1 File *revised* according to the dendro-^{14}C placements reported in Table 2 (compare with Fig 7), and after removing the assumption of a crossdate between the Porsuk and Gordion chronologies and between the Porsuk and MBA chronologies.

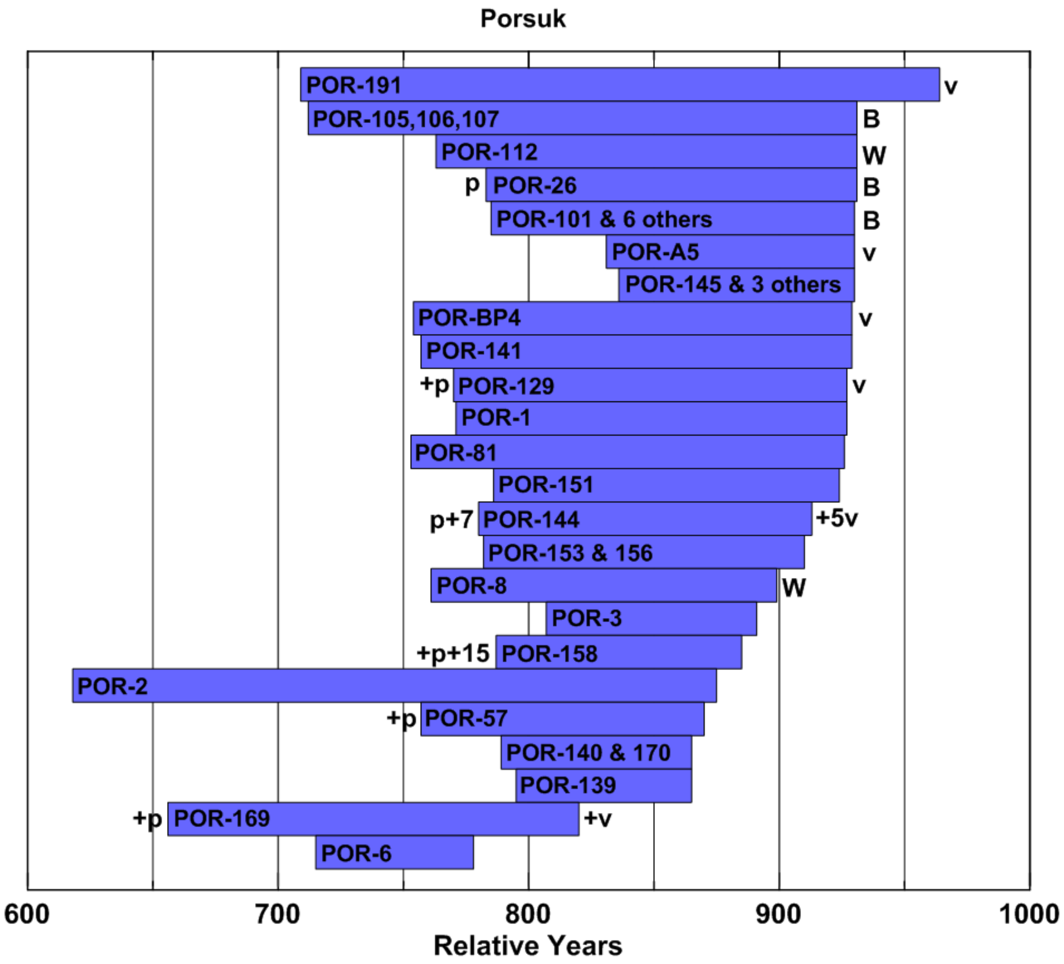

Fig C. The Porsuk (POR) chronology by individual sample (or group of samples from the same tree) shown in terms of the Porsuk Relative Years (RY). Annotations at the ends of the bars are: p = pith, B = bark, W = waney edge (the vascular cambium, i.e., last ring before bark), +N = additional ring(s) incomplete and/or not measured, and v = outer ring close to bark (whose determination is somewhat subjective). Unmarked ends indicate samples with an unknown number of absent rings, which were removed in shaping timbers for construction, from deterioration, or other unknown causes. For crossdating information, see S1 Dataset, Table C in S1 File.

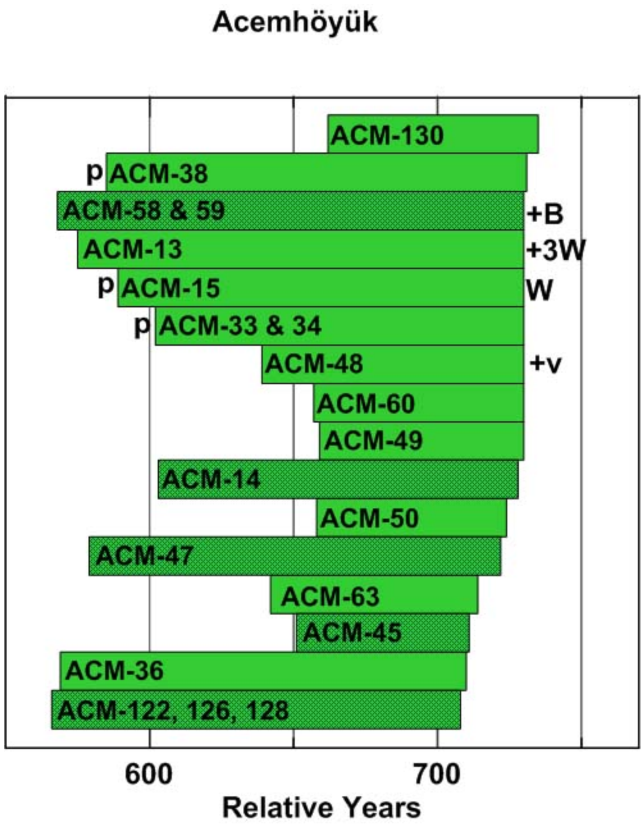

Fig D. The Acemhöyük (ACM) chronology by individual sample (or group of samples from the same tree) shown in terms of the MBA Relative Years (RY). Samples available but not employed in this study are dark-shaded. For annotations, see caption to Figure C in S1 File. For crossdating information, see S2 Dataset, Table C in S1 File.

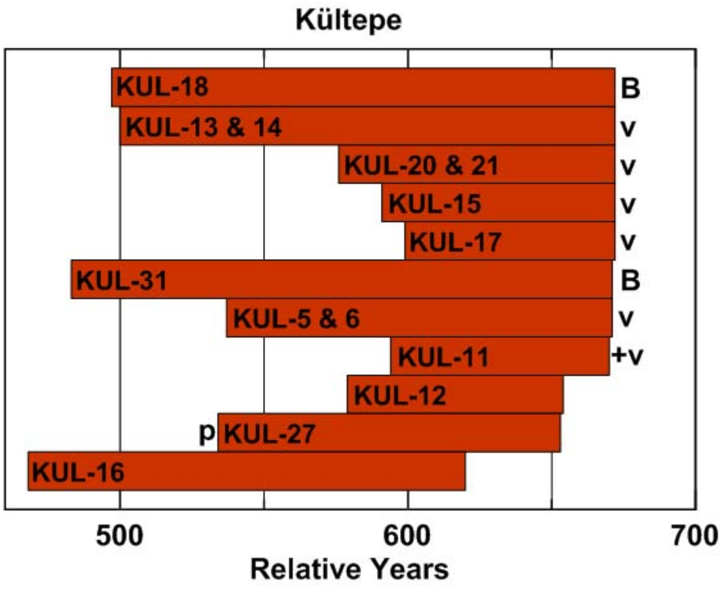

Fig E. The Kültepe (KUL) chronology by individual sample (or group of samples from the same tree) shown in terms of the MBA Relative Years (RY). For annotations, see caption to Figure C in S1 File. For crossdating information, see S3 Dataset, Table C in S1 File.

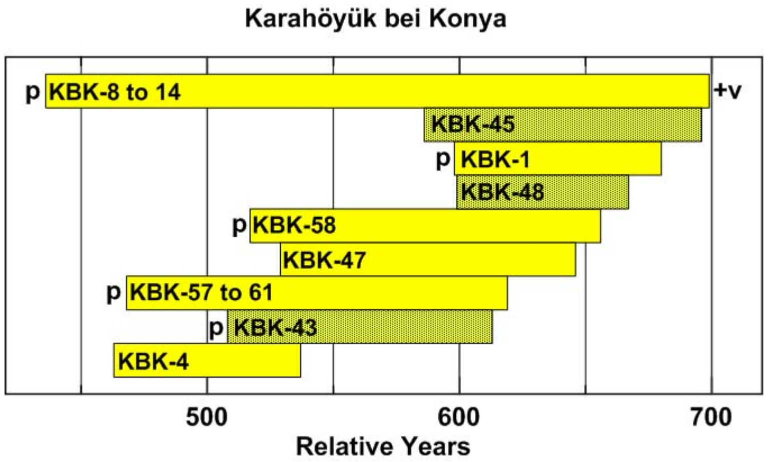

Fig F. The Karahöyük bei Konya (KBK) chronology by individual sample (or group of samples from the same tree) shown in terms of the MBA Relative Years (RY). Samples available but not employed in this study are dark-shaded. For annotations, see caption to Figure C in S1 File. For crossdating information, see Tables A, C in S1 File.

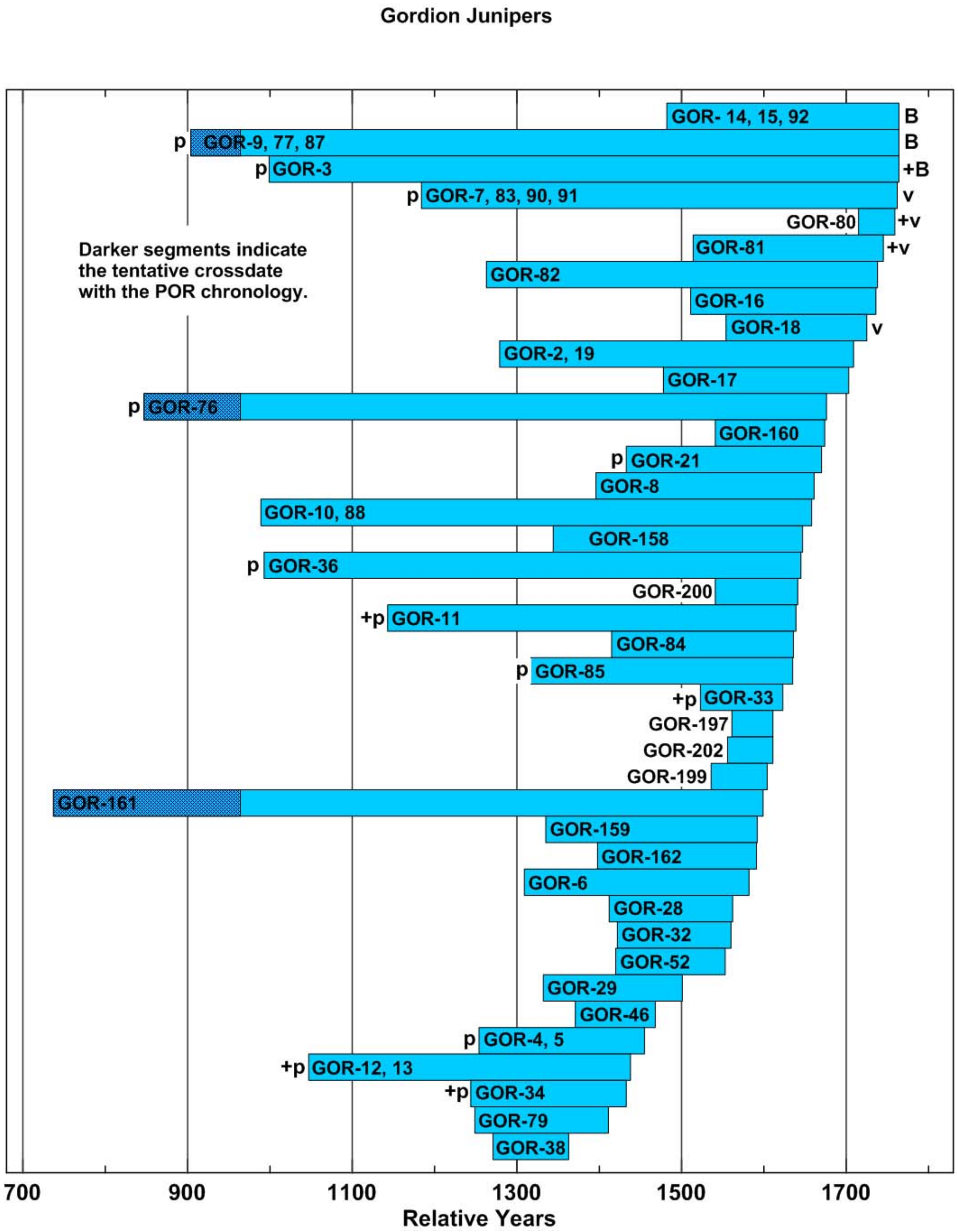

Fig G. The Gordion (GOR) juniper chronology by individual sample (or group of samples from the same tree) shown in terms of the Gordion Relative Years (RY) [21, 31]. The sequences of the only 3 GOR available samples (GOR-9 & 77 & 87; GOR-76; GOR-161), which offer a potential overlap with the Porsuk chronology, and which are employed in this study, are dark-shaded. For annotations, see caption to Figure C in S1 File. For crossdating information for the 3 samples employed in this study, see Table B in S1 File.

S1 Dataset, S2 Dataset, S3 Dataset and Tables A-D in S1 File summarize the separate site chronologies comprised of selected robust elements for ACM, KBK, KUL, POR and GOR (note: we show in Table B in S1 File only those 3 GOR samples which potentially overlap in temporal terms with the POR chronology). Fig 3 shows the selected robust sample numbers for the MBA chronological grouping of KUL-KBK-ACM and a summary of the crossdating statistics for this MBA chronology (together) versus the Porsuk (POR) chronology and for POR against GOR – the data cited in Fig 3 come from Table C in S1 File which shows the crossdating grid for the five individual site chronologies discussed and used in this paper (terminology as S1 Dataset, S2 Dataset, S3 Dataset, Tables A, B in S1 File). We see robust crossdates for KUL v. KBK and ACM v. KBK, especially; these combined site chronologies (KUL-KBK-ACM) form the basis of the MBA chronology. We note that KUL and ACM best crossdate in the same position also, but without the shared robust linkages with the KBK chronology, this crossdate would be less than clear-cut by itself. Whereas, altogether, the three sites form a secure chronology. The best available linkages of any of the MBA set elements with POR are less than secure, as is GOR with POR. There are only 1 or 2, or at most for a short period, 3 samples in the GOR chronology in these putative overlaps, which is too few for a secure crossdate, especially when there is not a strong statistical link nor good visual match (Fig 3, Tables C-E in S1 File).

See S1 Dataset, S2 Dataset, S3 Dataset, Tables A, B in S1 File.

Table A. Crossdating grid for the samples from Karahöyük bei Konya (KBK) employed in this study. See caption to S1 Dataset for other details.

	KBK1A	KBK4	KBK8,14	KBK47	KBK57,61
KBK4	*n*=0				
KBK8,14	t_{BP}= 4.2 GLK= 66** n= 83	t_{BP}= 6.8 GLK= 65** n= 75			
KBK47	t_{BP}= 0.1 GLK= 60 n= 49	t_{BP}= 0.6 GLK= 38 n= 9	t_{BP}= 6.3 GLK= 66*** n= 118		
KBK57,61	t_{BP}= 2.6 GLK= 67 n= 22	t_{BP}= 2.6 GLK= 51 n= 70	t_{BP}= 5.3 GLK= 67*** n= 152	t_{BP}= 4.0 GLK= 64** n= 91	
KBK58	t_{BP}= 4.6 GLK= 66** n= 59	t_{BP}= 0.8 GLK= 40 n= 11	t_{BP}= 7.9 GLK= 73*** n= 140	t_{BP}= 5.1 GLK= 68*** n= 118	t_{BP}= 3.9 GLK=65** n= 103

Table B. Crossdating grid for the 3 samples from the Gordion area chronology employed in this study and which temporally overlap with the Porsuk chronology. See caption to S1 Dataset for other details.

	GOR161	GOR76B
**GOR76B**	t_{BP}= 12.1<br>GLK= 64***<br>n= 753	
**GOR9,77,87**	t_{BP}= 10.3<br>GLK= 65***<br>n= 696	t_{BP}= 27.3<br>GLK= 75***<br>n= 773

Table C. Crossdating grid for the individual site chronologies in Fig 3, Figure B in S1 File. See caption to S1 Dataset for other details.

	ACM	GOR	KBK	KUL
**GOR**	*n= 0*			
**KBK**	**t_{BP}= 7.80<br>GLK= 76***<br>n= 131**	*n=0*		
**KUL**	t_{BP}= 4.50<br>GLK= 66***<br>n= 104	*n=0*	**t_{BP}= 6.50<br>GLK= 68***<br>n= 205**	
**POR**	t_{BP}= 3.40<br>GLK= 62**<br>n= 118	t_{BP}= 4.40<br>GLK= 67***<br>n= 228	t_{BP}= 2.10<br>GLK= 64**<br>n= 82	t_{BP}= 2.60<br>GLK= 67**<br>n= 55

The values in bold for ACM versus KBK, and KUL versus KBK, indicate strong, robust crossdates over long time intervals (131 years and 205 years). KUL versus ACM by itself indicates only a potential crossdate – but given that robust crossdates comparing KUL versus KBK and ACM versus KBK give the same dating placement, we can regard the 3-site MBA chronology as secure. POR does not have any robust or even moderate statistical or visual correlation with ACM, KBK or KUL. The best (and previously proposed) GOR-POR crossdate has only moderate statistical and visual correlation (Fig 3) – and, critically, despite the long overlap, it cannot be considered as robust since: (i) the GOR chronology comprises only 1 or 2 (or for a short period) 3 samples across the relevant period (Fig 3, Figures A-B in S1 File); and (ii) because even this best available crossdate does not appear robust on the basis of a COFECHA analysis (Table E in S1 File).

Table D (parts 1-4). Summary of COFECHA [38, 39] version 6.02P analyses of the separate POR, ACM, KUL and KBK chronologies as shown in Figures C-F in S1 File, S1 Dataset, S2 Dataset, S3 Dataset, Tables A-C in S1 File.

1. Porsuk (POR)

```
*C* Number of dated series          24    *C*
*O* Master series   618   964   347 yrs *O*
*F* Total rings in all series   3447    *F*
*E* Total dated rings checked   3376    *E*
*C* Series intercorrelation    0.674    *C*
*H* Average mean sensitivity   0.282    *H*
*A* Segments, possible problems    0    *A*
*** Mean length of series      143.6    ***
```

POR SAMPLES Figure C in S1 File - COFECHA ANALYSIS PART 5: CORRELATION OF SERIES BY SEGMENTS: POR 24 TREES

Correlations of 50-year dated segments, lagged 25 years
Flags: A = correlation under 0.3281 but highest as dated; * = correlation higher at other than dated position

Seq	Series	Time_span		650 699	675 724	700 749	725 774	750 799	775 824	800 849	825 874	850 899	875 924	900 949
1	POR191	709	964			.48	.47	.58	.57	.69	.68	.47	.54	.63
2	POR105etc	712	931			.69	.72	.64	.66	.73	.84	.77	.78	.79
3	POR112	763	931					.72	.67	.71	.80	.71	.53	.54
4	POR26	783	931						.41	.49	.57	.62	.65	.69
5	POR101etc	785	930						.70	.64	.71	.77	.65	.67
6	PORA5	831	930								.78	.66	.53	.53
7	POR145etc	836	930								.67	.72	.72	.78
8	PORBP4	754	929					.62	.71	.77	.71	.60	.45	.51
9	POR141	757	929					.69	.77	.86	.82	.76	.71	.75
10	POR129	770	927					.65	.64	.61	.69	.80	.67	.70
11	POR1	771	927					.69	.70	.78	.81	.79	.76	.79
12	POR81	753	926					.47	.69	.90	.89	.84	.79	.81
13	POR151	786	924						.42	.38	.60	.63	.55	
14	POR144	780	913						.65	.58	.68	.78	.68	
15	POR153+156	782	910						.60	.63	.72	.70	.59	
16	POR8	761	899					.67	.81	.86	.86	.81		
17	POR3	807	891							.72	.79	.65		
18	POR158	787	885						.77	.76	.75	.64		
19	POR2	618	875	.53	.57	.69	.77	.74	.86	.93	.81	.73		
20	POR57	757	870					.68	.61	.67	.71			
21	POR140+170	789	865						.79	.82	.85			
22	POR139	795	865						.52	.58	.57			
23	POR169	656	820	.53	.59	.71	.65	.49	.65					
24	POR6	715	778			.84	.84	.81						

2. Acemhöyük (ACM)

C	Number of dated series	11 *C*
O	Master series 569 735	167 yrs *O*
F	Total rings in all series	1168 *F*
E	Total dated rings checked	1158 *E*
C	Series intercorrelation	0.496 *C*
	NB: 0.532 excluding ACM15	
H	Average mean sensitivity	0.258 *H*
A	Segments, possible problems	5 *A*
***	Mean length of series	106.2 ***

ACM SAMPLES Figure D in S1 File - COFECHA ANALYSIS PART 5: CORRELATION OF SERIES BY SEGMENTS: ACM 11 TREES

Correlations of 50-year dated segments, lagged 25 years
Flags: A = correlation under 0.3281 but highest as dated; * = correlation higher at other than dated position

Seq	Series	Time_span		575 624	600 649	625 674	650 699	675 724	700 749
1	ACM130	662	735				.60	.56	.49
2	ACM38	585	731	.54	.60	.71	.57	.65	.64
3	ACM13	575	730	.25A	.46	.49	.48	.53	.54
4	ACM15	589	730	.25*	.07*	.13*	.39	.39	.37
5	ACM33+34	602	730		.37	.57	.61	.66	.66
6	ACM48	639	730			.44	.51	.67	.67
7	ACM60	657	730				.55	.50	.45
8	ACM49	659	730				.45	.59	.53
9	ACM50	658	724				.61	.62	
10	ACM63	642	714			.72	.74	.74	
11	ACM36	569	710	.21*	.49	.62	.57	.59	

3. Kültepe (KUL)

C	Number of dated series	11	*C*
O	Master series 468 672	205 yrs	*O*
F	Total rings in all series	1352	*F*
E	Total dated rings checked	1337	*E*
C	Series intercorrelation	0.677	*C*
H	Average mean sensitivity	0.308	*H*
A	Segments, possible problems	0	*A*
***	Mean length of series	122.9	***

KUL SAMPLES Figure E in S1 File - COFECHA ANALYSIS PART 5: CORRELATION OF SERIES BY SEGMENTS: KUL 11 trees

Correlations of 50-year dated segments, lagged 25 years
Flags: A = correlation under 0.3281 but highest as dated; * = correlation higher at other than dated position

Seq	Series	Time_span		475<br>524	500<br>549	525<br>574	550<br>599	575<br>624	600<br>649	625<br>674
1	KUL18	497	672	.68	.68	.70	.77	.72	.58	.62
2	KUL13+14	500	672		.69	.67	.75	.76	.64	.62
3	KUL20+21	576	672					.77	.70	.64
4	KUL15	591	672					.78	.72	.75
5	KUL17	599	672					.78	.80	.81
6	KUL31	483	671	.61	.51	.52	.66	.59	.45	.42
7	KUL5+6	537	671			.73	.73	.60	.56	.70
8	KUL11	594	670					.71	.71	.65
9	KUL12	579	654					.60	.70	.76
10	KUL27	534	653			.55	.60	.54	.47	.48
11	KUL16	468	620	.81	.78	.77	.80	.69		

4. Karahöyük bei Konya (KBK)

C	Number of dated series	6		*C*
O	Master series 436 699	264 yrs		*O*
F	Total rings in all series	832		*F*
E	Total dated rings checked	786		*E*
C	Series intercorrelation	0.485		*C*
H	Average mean sensitivity	0.246		*H*
A	Segments, possible problems	2		*A*
***	Mean length of series	138.7		***

KBK SAMPLES Figure F in S1 File - COFECHA ANALYSIS PART 5: CORRELATION OF SERIES BY SEGMENTS: KBK 6 trees

Correlations of 50-year dated segments, lagged 25 years
Flags: A = correlation under 0.3281 but highest as dated; * = correlation higher at other than dated position

Seq	Series	Time_span		450-499	475-524	500-549	525-574	550-599	575-624	600-649	625-674	650-699
1	KBK8-14	436	699	.55	.50	.35	.56	.52	.51	.47	.45	.53
2	KBK1	598	680						.48	.45	.53	.58
3	KBK58	517	656			.48	.44	.58	.53	.62	.61	
4	KBK47	529	646				.52	.47	.49	.32A		
5	KBK57-61	468	619	.40	.35	.32A	.36	.44	.52			
6	KBK4	463	537	.52	.50	.36						

Table E. Summary of COFECHA [38, 39] version 6.02P Part 5 analyses of the correlations of series (ACM, KBK, KUL, POR, GOR) by segments (50 years).

CYAN = POR versus KUL, KBK, ACM overlap period. Values 0.31 to 0.61
YELLOW = GOR versus POR overlap period. Values 0.16 to 0.4
Unsatisfactory values are underlined (less than 0.3281 = 99% confidence level for 50 year segments [39]). Often from experience r values ≥0.40 are regarded as indicating likely good robust crossdates in dendrochronology.

Correlations of 50-year dated segments, lagged 25 years
Flags: A = correlation under 0.3281 but highest as dated; * = correlation higher at other than dated position

Seq	Series	Time_span		450-499	475-524	500-549	525-574	550-599	575-624	600-649	625-674	650-699	675-724	700-749	725-774	750-799	775-824	800-849	825-874	850-899	875-924	900-949	925-974
1	KUL	468	672	.37	.34	.30*	.50	.48	.50	.42	.40												
2	KBK	436	699	.37	.34	.30*	.50	.56	.65	.66	.68	.50											
3	ACM	569	731					.55	.56	.51	.59	.55	.45	.48									
4	POR	618	964							.34	.31A	.32A	.47	.61	.54	.38	.37	.37*	.30A	.24A	.21A	.16*	.18*
5	GOR	737	1647												.40	.38	.37	.37	.30*	.24A	.21A	.16*	.18*

There are other samples from the five sites (some examples are indicated, but not employed in this study, in Figures A-G in S1 File), and plausible site chronologies for this MBA set comprising some of these additional samples. However, in Fig 3, we show the selected most robust and clean (in terms of statistical and visual correlation) sets and samples from which we took ^{14}C dates for the tree-ring-^{14}C-wiggle-match analyses.

The individual tree-ring measurements for each of the samples in Fig 3 and S1 Dataset, S2 Dataset, S3 Dataset, and Tables A-C in S1 File have been submitted to the International Tree Ring Databank (ITRDB) (http://www.ncdc.noaa.gov/data-access/paleoclimatology-data/datasets/tree-ring) with ITRDB codes TURK044-TURK047, except for the select Gordion data which are already available (http://www1.ncdc.noaa.gov/pub/data/paleo/treering/measurements/europe/turk029.rwl). The Gordion juniper chronology (Figure G in S1 File) as a whole comprises many more samples over a long timespan reaching from the period shown in Fig 3 and Figures A-B in S1 File through to the earlier 1st millennium BCE (Figure G in S1 File) [40, 41]. However, for the time period relevant to this paper and for investigating the validity of the claimed dendrochronological crossdate with the Porsuk chronology, the total Gordion sample numbers in this time period are as shown in Fig 3 and Figures B and G in S1 File. The potential overlap of Gordion to Porsuk involves just 1 or 2 (or for only a short time) 3 samples.

Specific tree-ring sections (according to Relative Years, RY) were dissected by steel blade under a binocular microscope from selected samples for ^{14}C dating. As detailed for each sample in Table F in S1 File, these typically comprised either 10 or 9 tree-rings, but in a few cases a smaller stated number of tree-rings. Table F in S1 File lists all the specific tree-ring samples and the ^{14}C measurements which were then employed in the dating models (as summarized in Table 2). Figure H in S1 File indicates, as an example, the dissected and dated sections from the Porsuk tree-ring record.

Table F. Samples of *Juniperus* spp. radiocarbon dated as part of this project (the Gordion juniper ^{14}C data, have been published previously [40]).

Lab ID	Site	Sample	Rings RY	δ^{13}C‰*	^{14}C BP	SD	Notes
		(all *Juniperus* spp.)					
Hd-22955	KBK	43	509-520	-22.8	3684	11	
Hd-22956	KBK	43	520-530	-22.6	3660	11	
Hd-22957	KBK	43	530-540	-23.1	3627	17	
Hd-22986	KBK	43	540-550	-23.2	3630	15	
Hd-23043	KBK	43	550-560	-23.0	3618	15	
Hd-22987	KBK	43	560-570	-22.9	3633	14	
Hd-23045	KBK	43	570-580	-23.2	3604	17	
Hd-23026	KBK	43	580-590	-22.4	3570	14	
Hd-23027	KBK	43	590-600	-22.6	3563	17	
Hd-22958	KBK	43	600-610	-22.2	3578	20	

OxA-29954	KUL	18C	497-505	-22.0	3647	27	
OxA-30900	KUL	18C	507-515	-22.3	3657	31	
OxA-29955	KUL	18C	517-525	-22.2	3618	28	
OxA-30901	KUL	18C	527-535	-22.4	3641	32	
OxA-29956	KUL	18C	537-545	-21.9	3570	28	
OxA-29957	KUL	18C	537-545	-22.0	3613	28	
OxA-30902	KUL	18C	543-545	-22.0	3675	30	
OxA-30903	KUL	18C	547-555	-21.7	3596	31	
OxA-29958	KUL	18C	557-565	-22.1	3678	29	
OxA-30904	KUL	18C	567-575	-22.4	3607	30	
OxA-30905	KUL	18C	567-575	-22.2	3575	33	
OxA-29959	KUL	18C	577-585	-22.2	3584	29	
OxA-30906	KUL	18C	587-595	-22.0	3572	31	
OxA-29960	KUL	18C	597-605	-22.3	3582	28	
OxA-30907	KUL	18C	607-615	-22.1	3669	29	#
OxA-29961	KUL	18C	617-625	-22.1	3539	27	
OxA-30908	KUL	18C	627-635	-22.4	3626	31	
OxA-29961	KUL	18C	637-645	-21.9	3522	28	
OxA-30909	KUL	18C	647-655	-22.3	3490	30	
OxA-29963	KUL	18C	656-672	-21.4	3457	28	
OxA-30890	ACM	34B	609-615	-22.1	3566	32	
OxA-31514	ACM	34B	609-615	-22.1	3564	32	
OxA-30891	ACM	34B	617-625	-22.6	3582	32	
OxA-31515	ACM	34B	617-625	-22.2	3613	31	
OxA-30892	ACM	34B	627-635	-22.1	3532	33	
OxA-31516	ACM	34B	627-635	-21.8	3535	31	
OxA-30893	ACM	34B	637-645	-21.9	3559	32	
OxA-31517	ACM	34B	637-645	-21.7	3473	32	
OxA-30894	ACM	34B	647-655	-22.1	3548	32	
OxA-31518	ACM	34B	647-655	-22.1	3495	31	
OxA-30895	ACM	34B	657-665	-22.0	3588	32	
OxA-31519	ACM	34B	657-665	-22.0	3540	31	
OxA-30896	ACM	34B	667-675	-22.2	3547	33	
OxA-31520	ACM	34B	667-675	-22.4	3528	31	
OxA-30897	ACM	34B	677-685	-21.9	3593	32	**
OxA-31521	ACM	34B	677-685	-22.2	3492	31	**
OxA-30898	ACM	34B	687-695	-21.8	3499	32	
OxA-31522	ACM	34B	687-695	-21.7	3452	39	
OxA-30899	ACM	34B	697-705	-21.3	3498	32	
OxA-31523	ACM	34B	697-705	-21.3	3470	31	
OxA-30935	POR	2F	694-702	-20.4	3507	33	

OxA-27203	POR	2A	723-732	-19.5	3512	29	
OxA-28694	POR	2A	723-732	-19.5	3458	28	
OxA-27204	POR	2A	733-742	-19.2	3495	29	
OxA-28695	POR	2A	733-742	-19.2	3487	28	
OxA-27202	POR	2A	743-752	-19.2	3535	29	
OxA-28693	POR	2A	743-752	-19.3	3494	27	
OxA-24627	POR	26A	783-792	-20.7	3431	30	
OxA-26240	POR	26A	783-792	-20.7	3393	27	
OxA-24628	POR	26A	793-802	-22.7	3364	30	
OxA-26241	POR	26A	793-802	-20.8	3426	27	
OxA-24585	POR	26A	803-812	-21.1	3436	30	
OxA-26243	POR	26A	803-812	-20.6	3385	26	
OxA-24724	POR	26A	813-822	-21.8	3355	28	
OxA-26244	POR	26A	813-822	-21.0	3423	27	
OxA-26245	POR	26A	813-822	-20.9	3369	27	
OxA-24629	POR	26A	823-832	-22.2	3426	29	
OxA-26246	POR	26A	823-832	-21.1	3386	27	
OxA-24586	POR	26A	833-842	-21.8	3371	30	
OxA-26247	POR	26A	833-842	-20.6	3404	27	
OxA-24587	POR	26A	843-852	-21.6	3380	29	
OxA-26248	POR	26A	843-852	-20.9	3385	26	
VERA-5750A_1	POR	26A	843-852	-21.2±4.4	3404	38	$
VERA-5750A_2	POR	26A	843-852	-22.9±3.1	3336	18	$
VERA-5750A_3	POR	26A	843-852	-24.2±0.7	3389	32	$
VERA-5750HS	POR	26A	843-852	-26.8±1.1	3345	39	+
VERA-5750B	POR	26A	843-852	-22.0±0.8	3386	28	@
VERA-5750C	POR	26A	843-852	-26.3±1.1	3346	32	&
OxA-24588	POR	26A	853-862	-22.6	3355	29	
OxA-26249	POR	26A	853-862	-20.3	3363	27	
VERA-5751A_1	POR	26A	853-862	-20.1±4.6	3365	39	$
VERA-5751A_2	POR	26A	853-862	-26.1±1.8	3363	20	$
VERA-5751A_3	POR	26A	853-862	-22.9±0.9	3400	30	$
VERA5751HS	POR	26A	853-862	-25.6±1.7	3354	48	+
VERA5751B	POR	26A	853-862	-24.0±0.8	3408	34	@
VERA5751C	POR	26A	853-862	-25.4±0.8	3358	29	&
OxA-24589	POR	26A	863-872	-21.7	3402	28	
OxA-26250	POR	26A	863-872	-21.1	3396	27	
OxA-26251	POR	26A	863-872	-21.2	3378	27	
VERA-5752A_1	POR	26A	863-872	-25.4±1.4	3366	36	$
VERA-5752A_2	POR	26A	863-872	-22.3±0.7	3390	23	$
VERA-5752A_3	POR	26A	863-872	-26.6±1.0	3410	31	$
VERA-5752HS	POR	26A	863-872	-22.0±2.4	3348	53	+
VERA-5752B	POR	26A	863-872	-23.8±0.8	3410	27	@

VERA-5752C	POR	26A	863-872	-27.0±0.9	3390	30	&
OxA-24630	POR	26A	873-882	-23.5	3369	29	
OxA-26252	POR	26A	873-882	-20.7	3359	27	
OxA-30863	POR	26B	903-911	-22.0	3325	32	
OxA-30864	POR	26B	915-923	-22.3	3302	31	
OxA-30865	POR	26B	928-931	-21.9	3286	32	

Notes:

***** The OxA $\delta^{13}C$ data are stated as ±0.3‰ and from a separate stable isotope MS analysis. The VERA $\delta^{13}C$ data are measurements from the AMS with the errors as stated in the table. The Heidelberg $\delta^{13}C$ data are separate stable isotope MS measurements – the error is ~ 0.4‰.

OxA-30907 had a high offset between the $\delta^{13}C$ measured on the AMS versus the stable isotope MS (suggesting fractionation at the level of 1.1%). Sometimes this indicates an issue with a sample and an age offset and this sample is also identified as an outlier in the model runs.

****** The two ^{14}C measurements for ACM RY677-685 are not compatible with being the same ^{14}C age at the 5% level (T = 5.14 > 3.8) [60]. These two dates are not combined as a weighted average in the models (see Tables G-J in S1 File).

$ Sample with VERA standard ABA treatment

+ Sample dated comprises the humic acid extract from the same numbered A_2 sample [56].

@ Sample received soxhlet, AB and bleaching pretreatment

& Sample received soxhlet and ABA pretreatment

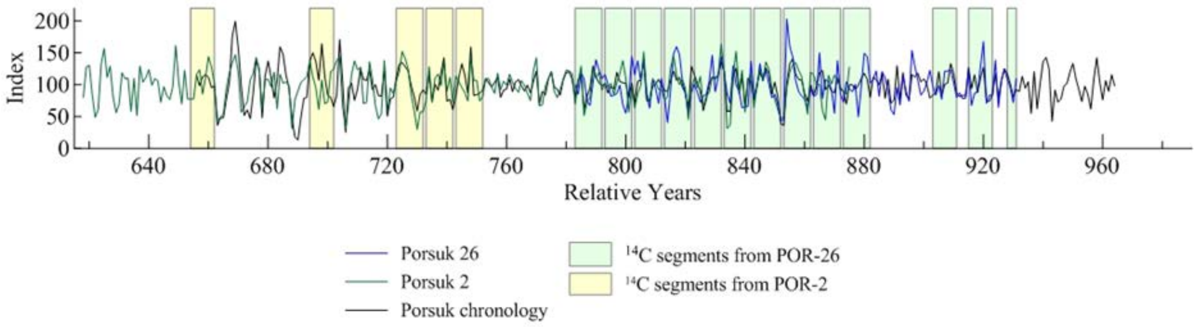

Figure H. The tree-ring growth record for samples POR-26 and POR-2 and the locations (in terms of the relative chronology) from where samples for ^{14}C dating were dissected.

B. ^{14}C known age checks (Oxford) and laboratory inter-comparisons

A routine known-age sample testing program is run at the Oxford Laboratory to assess on-going dating accuracy (for examples, see [50] at SOM Section 2.2 and Fig.S1, [Reference D in S1 File]). During the period when the samples dated in this project were run at Oxford (03/06/2011 to 02/04/2015), a total of 433 measurements were made on samples of known age wood and these indicate that 97.7% of dates were within 2σ with a systematic bias of 1.6±1.3 yr (older).

As shown in Fig 4, three samples were dated – and several times and employing different approaches – both at Oxford and Vienna and all measurements produced compatible ^{14}C ages within 95% confidence limits [60]. Figure I in S1 File provides further details on the comparison by lab and sample pretreatment regime. Previous comparisons or known age tests for the Oxford, Vienna and Heidelberg radiocarbon laboratories have yielded good and comparable data [40, 50, 57, 65, 91]. Small contributions from correlated uncertainties, which may arise when data obtained by the same laboratory are combined, were not considered in the uncertainty of the combined final, weighted mean values employed [60]. To indicate the scale of this minor issue, we note with regard to the VERA dates, where we have most data, that the differences between the weighted average values used (i), and the values trying to allow for correlated uncertainty (ii), are: VERA-5750 (i) 3359±12 versus (ii) 3358±13; VERA-5751 (i) 3374±13 versus (ii) 3373±14 and VERA-5752 (i) 3392±13 versus (ii) 3390±14. Such very small differences are not significant to the analysis. The Oxford KUL and ACM dates and Heidelberg KBK dates were run on juniper wood from different sites, but in those instances where the data are similarly placed in calendar terms (similar RY ranges within <5 years) all the ^{14}C ages are similar and overlap within their 95% ranges.

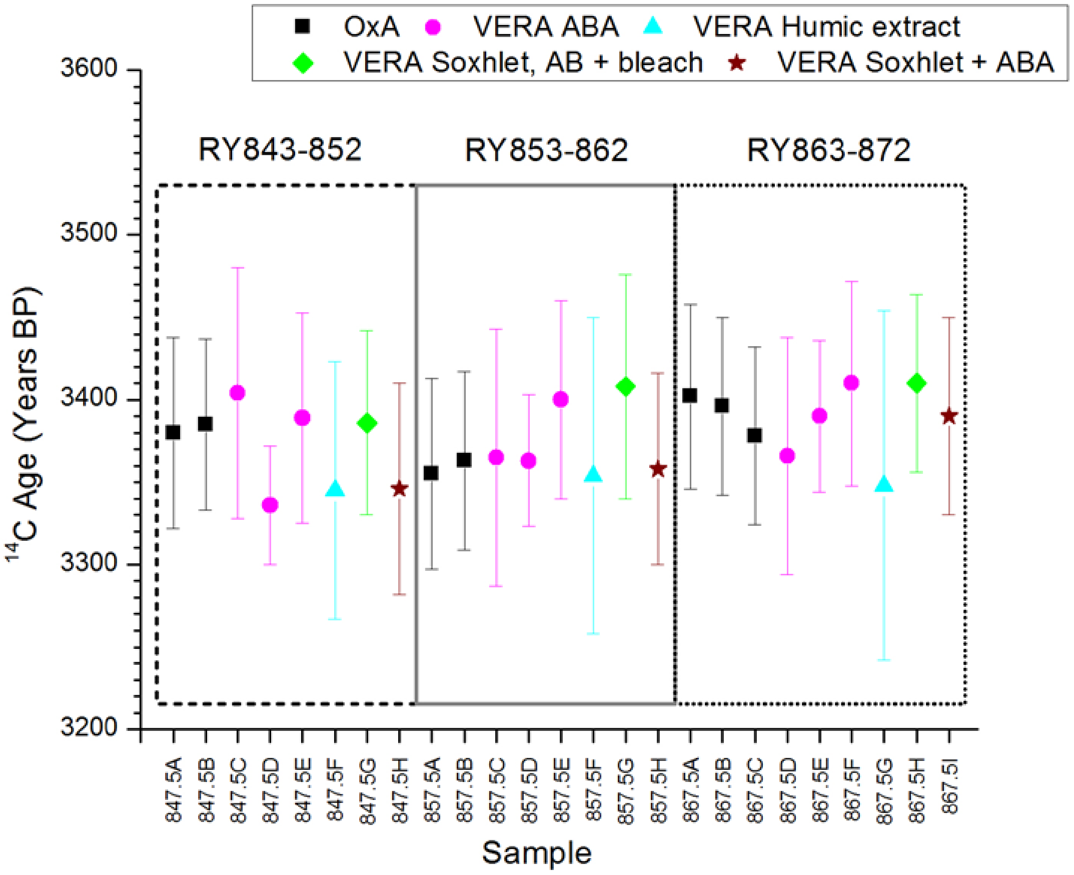

Figure I. Comparison of the ^{14}C measurements on the same Porsuk tree-ring samples between the Oxford (OxA) and Vienna (VERA) AMS laboratories. The samples compared include those pretreated in similar fashion (VERA, ABA method) but also VERA data for measurements on extracted humic acids [56], and on samples receiving Soxhlet extraction and then AB plus bleaching and Soxhlet extraction plus ABA treatment. Data from Table F in S1 File with 2σ (95.4%) errors shown – all measurements for each set of the identical tree-rings are compatible with being estimates of the same ^{14}C age [60].

C. Chronological modelling and analysis – further details

Four examples of the Bayesian dendro-^{14}C-wiggle-match code used, for Models 3, 6b, 8a and 8b, are listed in Tables G-J in S1 File and examples of outputs are shown in Table 2, Figs 5, 6, and Figures J-M in S1 File. For details on the modelling, see the methods discussion in the main text. For some further discussion, see below in this section.

Table G. OxCal runfile code for Model 3 (Table 2)

```
Options()
 {
  Resolution=1;
 };
 Plot( )
 {
  Outlier_Model("RScaled",T(5),U(0,4),"r");
  D_Sequence("Model 3 = Gordion n128 minus outliers")
  {
   First( );
   Date ("776");
   Gap (0.5);
   R_Date("776.5", 3430, 14)
   {
    Outlier (0.05);
   };
   Gap( 10);
   R_Date("786.5", 3409, 13)
   {
    Outlier (0.05);
   };
   Gap( 10);
   R_Date("796.5", 3432, 16)
   {
    Outlier (0.05);
   };
   Gap( 10);
   R_Date("806.5", 3426, 13)
   {
    Outlier (0.05);
   };
   Gap( 10);
   R_Date("816.5", 3403, 18)
   {
    Outlier (0.05);
   };
   Gap( 20);
   //R_Date("826.5", 3335, 16)
   //{
   // Outlier (0.05);
   //};
   //Gap( 10);
   R_Date("836.5", 3348, 17)
   {
    Outlier (0.05);
   };
   Gap( 10);
   R_Date("846.5", 3401, 16)
   {
    Outlier (0.05);
   };
   Gap(7.5);
   Date ("GOR Placement RY854");
   Gap (2.5);
   R_Date("856.5", 3372, 13)
   {
    Outlier (0.05);
   };
   Gap( 10);
   R_Date("866.5", 3356, 18)
   {
    Outlier (0.05);
   };
   Gap( 10);
   R_Date("876.5", 3342, 16)
   {
    Outlier (0.05);
   };
   Gap( 10);
   R_Date("886.5", 3334, 18)
   {
    Outlier (0.05);
   };
   Gap( 10);
   R_Date("896.5", 3336, 19)
   {
    Outlier (0.05);
   };
   Gap( 10);
   R_Date("906.5a", 3280, 16)
   {
    Outlier (0.05);
   };
   Gap( 10);
   //R_Date("906.5b", 3235, 17)
   //{
   // Outlier (0.05);
   //};
```

```
//Gap( 10);
//R_Date("916.5a", 3329, 14)
//{
// Outlier (0.05);
//};
//Gap( 0);
R_Date("916.5b", 3302, 14)
{
 Outlier (0.05);
};
Gap( 10);
//R_Date("925.5", 3323, 22)
//{
// Outlier (0.05);
//};
//Gap( 1);
R_Date("926.5a", 3308, 16)
{
 Outlier (0.05);
};
Gap( 0);
R_Date("926.5b", 3284, 17)
{
 Outlier (0.05);
};
Gap( 9);
R_Date("935.5", 3329, 23)
{
 Outlier (0.05);
};
Gap( 1);
//R_Date("936.5a", 3338, 18)
//{
// Outlier (0.05);
//};
//Gap( 0);
R_Date("936.5b", 3296, 18)
{
 Outlier (0.05);
};
Gap( 9);
R_Date("945.5", 3330, 21)
{
 Outlier (0.05);
};
Gap( 1);
R_Date("946.5a", 3289, 18)
{
 Outlier (0.05);
};
Gap( 0);
R_Date("946.5b", 3288, 22)
{
 Outlier (0.05);
};
Gap( 10);
R_Date("956.5a", 3322, 19)
{
 Outlier (0.05);
};
Gap( 0);
R_Date("956.5b", 3310, 18)
{
 Outlier (0.05);
};
Gap( 10);
R_Date("966.5a", 3319, 18)
{
 Outlier (0.05);
};
Gap( 0);
R_Date("966.5b", 3292, 16)
{
 Outlier (0.05);
};
Gap( 10);
R_Date("976.5", 3292, 23)
{
 Outlier (0.05);
};
Gap( 29);
//R_Date("986.5", 3223, 19)
//{
// Outlier (0.05);
//};
//Gap( 19);
R_Date("1005.5", 3257, 18)
{
 Outlier (0.05);
};
Gap( 10);
R_Date("1015.5", 3218, 18)
{
 Outlier (0.05);
};
Gap( 10);
R_Date("1025.5", 3203, 20)
{
 Outlier (0.05);
};
Gap( 10);
R_Date("1035.5", 3210, 21)
{
 Outlier (0.05);
};
Gap( 10);
R_Date("1045.5", 3202, 17)
{
 Outlier (0.05);
};
Gap( 19);
//R_Date("1054.5", 3160, 22)
```

```
//{
// Outlier (0.05);
//};
//Gap( 10);
R_Date("1064.5", 3195, 21)
{
 Outlier (0.05);
};
Gap( 10);
R_Date("1074.5", 3157, 32)
{
 Outlier (0.05);
};
Gap( 10);
R_Date("1084.5", 3124, 21)
{
 Outlier (0.05);
};
Gap( 10);
R_Date("1094.5", 3134, 19)
{
 Outlier (0.05);
};
Gap( 20);
//R_Date("1104.5", 3050, 20)
//{
// Outlier (0.05);
//};
//Gap( 10);
R_Date("1114.5", 3072, 17)
{
 Outlier (0.05);
};
Gap( 20);
//R_Date("1124.5", 3046, 18)
//{
// Outlier (0.05);
//};
//Gap( 10);
R_Date("1134.5", 3074, 18)
{
 Outlier (0.05);
};
Gap( 0.5);
R_Date("1135a", 3106, 18)
{
 Outlier (0.05);
};
Gap( 0);
R_Date("1135b", 3062, 24)
{
 Outlier (0.05);
};
Gap( 9.5);
R_Date("1144.5", 3049, 16)
{
 Outlier (0.05);
};
Gap( 1);
R_Date("1145.5", 3045, 21)
{
 Outlier (0.05);
};
Gap( 10);
//R_Date("1155", 3140, 38)
//{
// Outlier (0.05);
//};
//Gap( 0.5);
R_Date("1155.5", 3046, 21)
{
 Outlier (0.05);
};
Gap( 39);
//R_Date("1175", 3122, 18)#
//{
// Outlier (0.05);
//};
//Gap( 19.5);
//R_Date("1185", 3144, 18)
//{
// Outlier (0.05);
//};
//Gap( 9.5);
R_Date("1194.5", 3088, 13)
{
 Outlier (0.05);
};
Gap( 0.5);
R_Date("1195", 3060, 19)
{
 Outlier (0.05);
};
Gap( 9.5);
R_Date("1204.5", 3055, 13)
{
 Outlier (0.05);
};
Gap( 10.5);
//R_Date("1214.5", 3090, 18)
//{
// Outlier (0.05);
//};
//Gap( 0.5);
R_Date("1215", 3058, 25)
{
 Outlier (0.05);
};
Gap( 49.5);
//R_Date("1224.5", 2996, 17)
//{
// Outlier (0.05);
```

```
//};
//Gap( 40);
R_Date("1264.5", 3031, 20)
{
 Outlier (0.05);
};
Gap( 10);
R_Date("1274.5", 2992, 18)
{
 Outlier (0.05);
};
Gap( 10);
R_Date("1284.5", 3037, 23)
{
 Outlier (0.05);
};
Gap( 20);
//R_Date("1294.5", 3051, 21)
//{
// Outlier (0.05);
//};
//Gap( 10);
R_Date("1304.5", 2962, 20)
{
 Outlier (0.05);
};
Gap( 10);
R_Date("1314.5", 2970, 18)
{
 Outlier (0.05);
};
Gap( 10);
R_Date("1324.5", 2920, 18)
{
 Outlier (0.05);
};
Gap( 0.5);
R_Date("1325", 2909, 28)
{
 Outlier (0.05);
};
Gap( 9.5);
R_Date("1334.5", 2956, 23)
{
 Outlier (0.05);
};
Gap( 0.5);
R_Date("1335", 2929, 20)
{
 Outlier (0.05);
};
Gap( 9.5);
R_Date("1344.5", 2937, 19)
{
 Outlier (0.05);
};
Gap( 0.5);
R_Date("1345", 2962, 20)
{
 Outlier (0.05);
};
Gap( 9.5);
R_Date("1354.5", 2970, 19)
{
 Outlier (0.05);
};
Gap( 10);
R_Date("1364.5", 2936, 20)
{
 Outlier (0.05);
};
Gap( 10);
R_Date("1374.5", 2955, 14)
{
 Outlier (0.05);
};
Gap( 0.5);
R_Date("1375", 2955, 20)
{
 Outlier (0.05);
};
Gap( 10);
R_Date("1385", 2946, 19)
{
 Outlier (0.05);
};
Gap( 9.5);
R_Date("1394.5", 2948, 15)
{
 Outlier (0.05);
};
Gap( 0.5);
R_Date("1395", 2938, 23)
{
 Outlier (0.05);
};
Gap( 9.5);
R_Date("1404.5", 2907, 17)
{
 Outlier (0.05);
};
Gap( 0.5);
R_Date("1405", 2899, 26)
{
 Outlier (0.05);
};
Gap( 9.5);
R_Date("1414.5", 2912, 17)
{
 Outlier (0.05);
};
Gap( 0.5);
```

```
R_Date("1415", 2918, 23)
{
 Outlier (0.05);
};
Gap( 9.5);
R_Date("1424.5", 2929, 15)
{
 Outlier (0.05);
};
Gap( 20);
//R_Date("1434.5", 2935, 17)
//{
// Outlier (0.05);
//};
//Gap( 10);
R_Date("1444.5", 2871, 19)
{
 Outlier (0.05);
};
Gap( 20);
//R_Date("1445", 2942, 21)
//{
// Outlier (0.05);
//};
//Gap( 9.5);
//R_Date("1454.5", 2935, 16)
//{
// Outlier (0.05);
//};
//Gap( 10);
R_Date("1464.5", 2896, 15)
{
 Outlier (0.05);
};
Gap( 20);
//R_Date("1474.5", 2934, 14)
//{
// Outlier (0.05);
// };
// Gap( 10);
R_Date("1484.5", 2880, 15)
{
 Outlier (0.05);
};
Gap( 0.5);
R_Date("1485", 2868, 20)
{
 Outlier (0.05);
};
Gap( 20);
//R_Date("1495", 2899, 14)
//{
// Outlier (0.05);
//};
//Gap( 10);
R_Date("1505", 2848, 13)
{
 Outlier (0.05);
};
Gap( 20);
//R_Date("1515", 2895, 20)
//{
// Outlier (0.05);
//};
//Gap( 10);
R_Date("1525", 2800, 15)
{
 Outlier (0.05);
};
Gap( 10);
R_Date("1535", 2816, 20)
{
 Outlier (0.05);
};
Gap( 10);
R_Date("1545", 2779, 22)
{
 Outlier (0.05);
};
Gap( 10);
R_Date("1555", 2811, 20)
{
 Outlier (0.05);
};
Gap( 30);
R_Date("1585", 2786, 20)
{
 Outlier (0.05);
};
Gap( 20);
R_Date("1605", 2728, 26)
{
 Outlier (0.05);
};
Gap( 19.5);
R_Date("1624.5", 2746, 25)
{
 Outlier (0.05);
};
Gap( 10);
//R_Date("1625", 2777, 20)
//{
// Outlier (0.05);
//};
//Gap( 9.5);
R_Date("1634.5", 2748, 18)
{
 Outlier (0.05);
};
Gap( 0.5);
R_Date("1635", 2709, 13)
{
```

```
 Outlier (0.05);
};
Gap( 9.5);
R_Date("1644.5", 2743, 18)
{
 Outlier (0.05);
};
Gap( 0.5);
R_Date("1645", 2734, 20)
{
 Outlier (0.05);
};
Gap( 10);
R_Date("1655", 2746, 20)
{
 Outlier (0.05);
};
Gap( 10);
R_Date("1665", 2760, 25)
{
 Outlier (0.05);
};
Gap( 9.5);
R_Date("1674.5", 2730, 16)
{
 Outlier (0.05);
};
Gap( 0.5);
R_Date("1675", 2720, 20)
{
 Outlier (0.05);
};
Gap( 9.5);
R_Date("1684.5", 2683, 23)
{
 Outlier (0.05);
};
Gap( 10);
//R_Date("1685", 2744, 18)
//{
// Outlier (0.05);
//};
//Gap( 9.5);
R_Date("1694.5", 2712, 18)
{
 Outlier (0.05);
};
Gap( 0.5);
R_Date("1695", 2662, 13)
{
 Outlier (0.05);
};
Gap( 10);
R_Date("1705", 2640, 16)
{
 Outlier (0.05);
};
Gap( 10);
//R_Date("1714.5", 2666, 18)
//{
// Outlier (0.05);
//};
//Gap( 0.5);
R_Date("1715", 2616, 12)
{
 Outlier (0.05);
};
Gap( 9.5);
R_Date("1724.5", 2589, 21)
{
 Outlier (0.05);
};
Gap( 0.5);
R_Date("1725a", 2589, 17)
{
 Outlier (0.05);
};
Gap( 0);
R_Date("1725b", 2549, 19)
{
 Outlier (0.05);
};
Gap( 10);
//R_Date("1734.5", 2610, 17)
//{
// Outlier (0.05);
//};
//Gap( 0.5);
//R_Date("1735a", 2608, 19)
//{
// Outlier (0.05);
//};
//Gap( 0);
R_Date("1735b", 2543, 17)
{
 Outlier (0.05);
};
Gap( 9.5);
R_Date("1744.5", 2549, 21)
{
 Outlier (0.05);
};
Gap( 0.5);
R_Date("1745a", 2555, 17)
{
 Outlier (0.05);
};
Gap( 0);
R_Date("1745b", 2502, 36)
{
 Outlier (0.05);
};
```

```
 Gap( 9.5);
 R_Date("1754.5", 2530, 25)
 {
  Outlier (0.05);
 };
 };
};
```

Notes: Samples (and lines of code) marked // are the outliers excluded from Models 1 and 2 in Model 3.
(Not part of the OxCal runfile.) The date centered RY1175 is the sole outlier in Model 2 and is then excluded in Model 3.

Table H. OxCal runfile code for Model 6a (Table 2)

```
Options()
 {
  Resolution=1;
 };
 Plot()
 {
  Outlier_Model("RScaled",T(5),U(0,4),"r");
  D_Sequence("Porsuk, OxA & VERA separate")
  {
   R_Date ("OxA-30935 2F RY694-702 = 698", 3507, 33)
   {
    Outlier(0.05);
   };
   Gap (29.5);
   R_Combine ("2A RY723-732 = 727.5")
   {
    R_Date ("OxA-27203 RY723-732", 3512, 29);
    R_Date ("OxA-28694 RY723-732", 3458, 28);
    Outlier(0.05);
   };
   Gap (10);
   R_Combine ("2A RY733-742 = 737.5")
   {
    R_Date ("OxA-27204 RY733-742", 3495, 29);
    R_Date ("OxA-28695 RY733-742", 3487, 28);
    Outlier(0.05);
   };
   Gap (10);
   R_Combine ("2A RY743-752 =747.5")
   {
    R_Date ("OxA-27202 RY743-752", 3535, 29);
    R_Date ("OxA-28693 RY743-752", 3494, 27);
    Outlier(0.05);
   };
   Gap (28.5);
   Date ("Ring 776");
   Gap (11.5);
   R_Combine("1-10 = RY783-792 = 787.5")
   {
    R_Date("OxA-24627", 3431, 30);
    R_Date("OxA-26240", 3393, 27);
    Outlier(0.05);
   };
   Gap(10);
   R_Combine("11-20 = RY793-802 = 797.5")
   {
    R_Date("OxA-24628", 3364, 30);
    R_Date("OxA-26241", 3426, 27);
    R_Date("OxA-26242", 3440, 27);
    Outlier(0.05);
   };
   Gap(10);
   R_Combine("21-30 = RY803-812 = 807.5")
   {
    R_Date("OxA-24585", 3436, 30);
    R_Date("OxA-26243", 3385, 26);
    Outlier(0.05);
   };
   Gap(10);
   R_Combine("31-40 = RY813-822 = 817.5")
   {
    R_Date("OxA-24724", 3355, 28);
    R_Date("OxA-26244", 3423, 27);
    R_Date("OxA-26245", 3369, 27);
    Outlier(0.05);
   };
   Gap(10);
   R_Combine("41-50 = RY823-832 =827.5")
   {
    R_Date("OxA-24629", 3426, 29);
    R_Date("OxA-26246", 3386, 27);
    Outlier(0.05);
   };
   Gap(10);
   R_Combine("51-60 = RY833-842 = 837.5")
   {
    R_Date("OxA-24586", 3371, 30);
    R_Date("OxA-26247", 3404, 27);
    Outlier(0.05);
   };
   Gap(10);
   R_Combine("OxA RY61-70 = RY843-852 = 847.5")
```

```
{
 R_Date("OxA-24587", 3380, 29);
 R_Date("OxA-26248", 3385, 26);
 Outlier(0.05);
};
Gap (0);
R_Combine ("VERA RY61-70 = RY843-852 = 847.5")
{
 R_Date("VERA-5750A_1", 3404, 38);
 R_Date("VERA-5750A_2", 3336, 18);
 R_Date("VERA-5750A_3", 3389, 32);
 R_Date("VERA-5750HS", 3345, 39);
 R_Date("VERA-5750B", 3386, 28);
 R_Date("VERA-5750C", 3346, 32);
 Outlier(0.05);
};
Gap(6.5);
Date ("Ring 854");
Gap (3.5);
R_Combine("OxA RY71-80 = RY853-862 = 857.5")
{
 R_Date("OxA-24588", 3355, 29);
 R_Date("OxA-26249", 3363, 27);
 Outlier(0.05);
};
R_Combine ("VERA RY71-80 = RY853-862 = 857.5")
{
 R_Date("VERA-5751A_1", 3365, 39);
 R_Date("VERA-5751A_2", 3363, 20);
 R_Date("VERA-5751A_3", 3400, 30);
 R_Date("VERA-5751HS", 3354, 48);
 R_Date("VERA-5751B", 3408, 34);
 R_Date("VERA-5751C", 3358, 29);
 Outlier(0.05);
};
Gap(10);
R_Combine("OxA RY81-90 = RY863-872 = 867.5")
{
 R_Date("OxA-24589", 3402, 28);
 R_Date("OxA-26250", 3396, 27);
 R_Date("OxA-26251", 3378, 27);
 Outlier(0.05);
};
Gap (0);
R_Combine ("VERA RY81-90 = RY863-872 = 867.5")
{
 R_Date("VERA-5752A_1", 3366, 36);
 R_Date("VERA-5752A_2", 3390, 23);
 R_Date("VERA-5752A_3", 3410, 31);
 R_Date("VERA-5752HS", 3348, 53);
 R_Date("VERA-5752B", 3410, 27);
 R_Date("VERA-5752C", 3390, 30);
 Outlier(0.05);
};
Gap(10);
R_Combine("91-100 = RY873-882 = 877.5")
{
 R_Date("OxA-24630", 3369, 29);
 R_Date("OxA-26252", 3359, 27);
 Outlier(0.05);
};
Gap (29.5);
R_Date ("OxA-30863 26B RY903-911 = 907", 3325, 32)
{
 Outlier(0.05);
};
Gap (12);
R_Date ("OxA-30864 26B RY915-923 = 919", 3302, 31)
{
 Outlier(0.05);
};
Gap (10.5);
R_Date ("OxA-30865 26B RY928-931 = 929.5", 3286,
32)
 {
 Outlier(0.05);
 };
 };
};
```

Table I. OxCal runfile code for Model 8a (Table 2)

```
Options()
 {
 Resolution=1;
 };
 Plot()
 {
 Outlier_Model("RScaled",T(5),U(0,4),"r");
 D_Sequence ("MBA = KUL + KBK + ACM")
 {
 R_Date ("OxA-29954 RY 497-505 = 501",3647,27)
 {
 Outlier ("RScaled",0.05);
 };
 Gap(10);
 R_Date ("OxA-30900 RY 507-515 = 511", 3657,31)
 {
 Outlier ("RScaled",0.05);
 };
 Gap(10);
```

```
//R_Date ("Hd-22955 RY 509-520 Mid-Point
514.5",3684,11)
//{
// Outlier ("RScaled",0.05);
//};
//Gap (6.5);
R_Date ("OxA-29955 RY 517-525 = 521",3618,28)
{
 Outlier ("RScaled",0.05);
};
Gap(4);
R_Date ("Hd-22956 RY 520-530 Mid-Point 525",3660,11)
{
 Outlier ("RScaled",0.05);
};
Gap (6);
R_Date ("OxA-30901 RY 527-535 = 531", 3641,32)
{
 Outlier ("RScaled",0.05);
};
Gap(4);
R_Date ("Hd-22957 RY 530-540 Mid-Point 535",3627,17)
{
 Outlier ("RScaled",0.05);
};
Gap (6);
R_Combine ("RY 537-545 = 541")
{
 R_Date ("OxA-29956",3570,28);
 R_Date ("OxA-29957", 3613,28);
 Outlier ("RScaled",0.05);
};
Gap(3);
R_Date ("OxA-30902 RY 543-545 = 544", 3675,30)
{
 Outlier ("RScaled",0.05);
};
Gap(1);
R_Date ("Hd-22986 RY 540-550 Mid-Point 545",3630,15)
{
 Outlier ("RScaled",0.05);
};
Gap (6);
R_Date ("OxA-30903 RY 547-555 = 551", 3596,31)
{
 Outlier ("RScaled",0.05);
};
Gap(4);
R_Date ("Hd-23043 RY 550-560 Mid-Point 555",3618,15)
{
 Outlier ("RScaled",0.05);
};
Gap (10);
//R_Date ("OxA-29958 RY 557-565 = 561", 3678,29)
//{
// Outlier ("RScaled",0.05);
//};
//Gap(4);
R_Date ("Hd-22987 RY 560-570 Mid-Point 565",3633,14)
{
 Outlier ("RScaled",0.05);
};
Gap (6);
R_Combine ("RY 567-575 = 571")
{
 R_Date ("OxA-30904 RY 567-575 = 571", 3607,30);
 R_Date ("OxA-30905 RY 567-575 = 571", 3575,33);
 Outlier ("RScaled",0.05);
};
Gap(4);
R_Date ("Hd-23045 RY 570-580 Mid-Point 575",3604,17)
{
 Outlier ("RScaled",0.05);
};
Gap (6);
R_Date ("OxA-29959 RY 577-585 = 581", 3584,29)
{
 Outlier ("RScaled",0.05);
};
Gap(4);
R_Date ("Hd-23026 RY 580-590 Mid-Point 585",3570,14)
{
 Outlier ("RScaled",0.05);
};
Gap (6);
R_Date ("OxA-30906 RY 587-595 = 591", 3572,31)
{
 Outlier ("RScaled",0.05);
};
Gap(4);
R_Date ("Hd-23027 RY 590-600 Mid-Point 595",3563,17)
{
 Outlier ("RScaled",0.05);
};
Gap (6);
R_Date ("OxA-29960 RY 597-605 = 601", 3582,28)
{
 Outlier ("RScaled",0.05);
};
Gap(4);
R_Date ("Hd-22958 RY 600-610 Mid-Point 605",3578,20)
{
 Outlier ("RScaled",0.05);
};
Gap (7);
//R_Date ("OxA-30907 RY 607-615 = 611", 3669, 29)
//{
// Outlier ("RScaled",0.05);
//};
//NB high offset δ13C AMS v MS - grounds to be
suspicious
//Gap(1);
```

```
R_Combine ("RY 609-615 = 612")
{
 R_Date ("OxA-30890 RY 609-615 = 612", 3566,32);
 R_Date ("OxA-31514 RY 609-615 = 612", 3564,32);
 Outlier ("RScaled",0.05);
};
Gap(9);
R_Date ("OxA-29961 RY 617-625 = 621", 3539,27)
{
 Outlier ("RScaled",0.05);
};
Gap(0);
R_Combine ("RY 617-625 = 621")
{
 R_Date ("OxA-30892 RY 617-625 = 621", 3582,32);
 R_Date ("OxA-31515 RY 617-625 = 621", 3613,31);
 Outlier ("RScaled",0.05);
};
Gap(10);
//R_Date ("OxA-30908 RY 627-635 = 631", 3626,31)
//{
// Outlier ("RScaled",0.05);
//};
//Gap(0);
R_Combine ("RY 627-635 = 631")
{
 R_Date ("OxA-30892 RY 627-635 = 631", 3532,33);
 R_Date ("OxA-31516 RY 627-635 = 631", 3535,31);
 Outlier ("RScaled",0.05);
};
Gap(10);
R_Date ("OxA-29962 RY 637-645 = 641", 3522,28)
{
 Outlier ("RScaled",0.05);
};
Gap(0);
R_Combine ("RY 637-645 = 641")
{
 R_Date ("OxA-30893 RY 637-645 = 641", 3559,32);
 R_Date ("OxA-31517 RY 637-645 = 641", 3473,32);
 Outlier ("RScaled",0.05);
};
Gap(10);
R_Date ("OxA-30909 RY 647-655 = 651", 3490,30)
{
 Outlier ("RScaled",0.05);
};
Gap(0);
R_Combine ("RY 647-655 = 651")
{
 R_Date ("OxA-30894 RY 647-655 = 651", 3548,32);
 R_Date ("OxA-31518 RY 647-655 = 651", 3495,31);
 Outlier ("RScaled",0.05);
};
Gap(8);
R_Date ("OxA-29963 RY 656-662 = 659", 3457,28)
{
 Outlier ("RScaled",0.05);
};
Gap(22);
//R_Combine ("RY 657-665 = 661")
//{
// R_Date ("OxA-30895 RY 657-665 = 661", 3588,32);
// R_Date ("OxA-31519 RY 657-665 = 661", 3540,31);
// Outlier ("RScaled",0.05);
//};
//Gap(10);
//R_Combine ("RY 667-675 = 671")
//{
// R_Date ("OxA-30896 RY 667-675 = 671", 3547,33);
// R_Date ("OxA-31520 RY 667-675 = 671", 3528,31);
// Outlier ("RScaled",0.05);
//};
//Gap(10);
// R_Date ("OxA-30897 RY 677-685 = 681", 3593,32) §
//{
//  Outlier ("RScaled",0.05);
// };
//Gap(0);
R_Date ("OxA-31521 RY 677-685 = 681", 3492,31)§
{
 Outlier ("RScaled",0.05);
};
Gap(10);
R_Combine ("RY 687-695 = 691")
{
 R_Date ("OxA-30898 RY 687-695 = 691", 3499,32);
 R_Date ("OxA-31522 RY 687-695 = 691", 3452,39);
 Outlier ("RScaled",0.05);
};
Gap(10);
R_Combine ("RY 677-685 = 701")
{
 R_Date ("OxA-30899 RY 697-705 = 701", 3498,32);
 R_Date ("OxA-31523 RY 697-705 = 701", 3470,31);
 Outlier ("RScaled",0.05);
};
Gap(75);
Date ("Ring 776 Estimate");
Gap (78);
Date ("Ring 854 Estimate");
};
};
```

***Notes*:** Samples (and lines of code) marked // are the outliers excluded from Model 7 in Model 8a. § (Not part of the OxCal runfile code.) The two ^{14}C dates OxA-30897 RY 677-685 = 681 and

OxA-31521 RY 677-685 = 681 are on the same age tree-rings but the ^{14}C ages are not compatible at the 95% level with being estimates of the same ^{14}C age [60] – hence we treat them as separate dates in the model (and not as a weighted average). In Model 7a OxA-30897 is identified as an outlier and hence is not included in Model 8a.

Table J. OxCal runfile cose for Model 8b (Table 2)

```
Options()
 {
  Resolution=1;
 };
 Plot()
 {
  Outlier_Model("RScaled",T(5),U(0,4),"r");
  D_Sequence ("MBA = KUL + KBK + ACM")
  {
   R_Date ("OxA-29954 RY 497-505 = 501",3647,27)
   {
    Outlier ("RScaled",0.05);
   };
   Gap(10);
   R_Date ("OxA-30900 RY 507-515 = 511", 3657,31)
   {
    Outlier ("RScaled",0.05);
   };
   Gap(10);
   //R_Date ("Hd-22955 RY 509-520 Mid-Point
514.5",3684,11)
   // {
   //  Outlier ("RScaled",0.05);
   // };
   // Gap (6.5);
   R_Date ("OxA-29955 RY 517-525 = 521",3618,28)
   {
    Outlier ("RScaled",0.05);
   };
   Gap(4);
   R_Date ("Hd-22956 RY 520-530 Mid-Point 525",3660,11)
   {
    Outlier ("RScaled",0.05);
   };
   Gap (6);
   R_Date ("OxA-30901 RY 527-535 = 531", 3641,32)
   {
    Outlier ("RScaled",0.05);
   };
   Gap(4);
   R_Date ("Hd-22957 RY 530-540 Mid-Point 535",3627,17)
   {
    Outlier ("RScaled",0.05);
   };
   Gap (6);
   R_Combine ("RY 537-545 = 541")
   {
    R_Date ("OxA-29956",3570,28);
    R_Date ("OxA-29957", 3613,28);
    Outlier ("RScaled",0.05);
   };
   Gap(3);
   R_Date ("OxA-30902 RY 543-545 = 544", 3675,30)
   {
    Outlier ("RScaled",0.05);
   };
   Gap(1);
   R_Date ("Hd-22986 RY 540-550 Mid-Point 545",3630,15)
   {
    Outlier ("RScaled",0.05);
   };
   Gap (6);
   R_Date ("OxA-30903 RY 547-555 = 551", 3596,31)
   {
    Outlier ("RScaled",0.05);
   };
   Gap(4);
   R_Date ("Hd-23043 RY 550-560 Mid-Point 555",3618,15)
   {
    Outlier ("RScaled",0.05);
   };
   Gap (10);
   //R_Date ("OxA-29958 RY 557-565 = 561", 3678,29)
   //{
   // Outlier ("RScaled",0.05);
   //};
   //Gap(4);
   R_Date ("Hd-22987 RY 560-570 Mid-Point 565",3633,14)
   {
    Outlier ("RScaled",0.05);
   };
   Gap (6);
   R_Combine ("RY 567-575 = 571")
   {
    R_Date ("OxA-30904 RY 567-575 = 571", 3607,30);
    R_Date ("OxA-30905 RY 567-575 = 571", 3575,33);
    Outlier ("RScaled",0.05);
   };
   Gap(4);
   R_Date ("Hd-23045 RY 570-580 Mid-Point 575",3604,17)
   {
    Outlier ("RScaled",0.05);
```

```
  };
  Gap (6);
  R_Date ("OxA-29959 RY 577-585 = 581", 3584,29)
  {
   Outlier ("RScaled",0.05);
  };
  Gap(4);
  R_Date ("Hd-23026 RY 580-590 Mid-Point 585",3570,14)
  {
   Outlier ("RScaled",0.05);
  };
  Gap (6);
  R_Date ("OxA-30906 RY 587-595 = 591", 3572,31)
  {
   Outlier ("RScaled",0.05);
  };
  Gap(4);
  R_Date ("Hd-23027 RY 590-600 Mid-Point 595",3563,17)
  {
   Outlier ("RScaled",0.05);
  };
  Gap (6);
  R_Date ("OxA-29960 RY 597-605 = 601", 3582,28)
  {
   Outlier ("RScaled",0.05);
  };
  Gap(4);
  R_Date ("Hd-22958 RY 600-610 Mid-Point 605",3578,20)
  {
   Outlier ("RScaled",0.05);
  };
  Gap (7);
  //R_Date ("OxA-30907 RY 607-615 = 611", 3669, 29)
  //{
  // Outlier ("RScaled",0.05);
  //};
  //NB high offset δ13C AMS v MS - grounds to be
suspicious
  //Gap(1);
  R_Combine ("RY 609-615 = 612")
  {
   R_Date ("OxA-30890 RY 609-615 = 612", 3566,32);
   R_Date ("OxA-31514 RY 609-615 = 612", 3564,32);
   Outlier ("RScaled",0.05);
  };
  Gap(9);
  R_Combine ("RY 617-625 KUL+ACM = 621")
  {
   R_Date ("OxA-29961 RY 617-625 = 621", 3539,27);
   R_Date ("OxA-30892 RY 617-625 = 621", 3582,32);
   R_Date ("OxA-31515 RY 617-625 = 621", 3613,31);
   Outlier ("RScaled",0.05);
  };
  Gap(10);
  R_Combine ("RY 627-635 KUL+ACM = 631")
  {
   R_Date ("OxA-30908 RY 627-635 = 631", 3626,31);
   R_Date ("OxA-30892 RY 627-635 = 631", 3532,33);
   R_Date ("OxA-31516 RY 627-635 = 631", 3535,31);
   Outlier ("RScaled",0.05);
  };
  Gap(10);
  R_Combine ("637-645 KUL+ACM = 641")
  {
   R_Date ("OxA-29962 RY 637-645 = 641", 3522,28);
   R_Date ("OxA-30893 RY 637-645 = 641", 3559,32);
   R_Date ("OxA-31517 RY 637-645 = 641", 3473,32);
   Outlier ("RScaled",0.05);
  };
  Gap(10);
  R_Combine ("647-655 KUL+ACM = 651")
  {
   R_Date ("OxA-30909 RY 647-655 = 651", 3490,30);
   R_Date ("OxA-30894 RY 647-655 = 651", 3548,32);
   R_Date ("OxA-31518 RY 647-655 = 651", 3495,31);
   Outlier ("RScaled",0.05);
  };
  Gap(8);
  R_Date ("OxA-29963 RY 656-672 = 659", 3457,28)
  {
   Outlier ("RScaled",0.05);
  };
  Gap(22);
  //R_Combine ("RY 657-665 = 661")
  // {
  // R_Date ("OxA-30895 RY 657-665 = 661", 3588,32);
  // R_Date ("OxA-31519 RY 657-665 = 661", 3540,31);
  // Outlier ("RScaled",0.05);
  //};
  // Gap(10);
  // R_Combine ("RY 667-675 = 671")
  // {
  //  R_Date ("OxA-30896 RY 667-675 = 671", 3547,33);
  //  R_Date ("OxA-31520 RY 667-675 = 671", 3528,31);
  //  Outlier ("RScaled",0.05);
  // };
  // Gap(10);
  // R_Date ("OxA-30897 RY 677-685 = 681", 3593,32) §
  // {
  //  Outlier ("RScaled",0.05);
  // };
  // Gap(0);
  R_Date ("OxA-31521 RY 677-685 = 681", 3492,31)§
  {
   Outlier ("RScaled",0.05);
  };
  Gap(10);
  R_Combine ("RY 687-695 = 691")
  {
   R_Date ("OxA-30898 RY 687-695 = 691", 3499,32);
   R_Date ("OxA-31522 RY 687-695 = 691", 3452,39);
   Outlier ("RScaled",0.05);
```

```
};
Gap(10);
R_Combine ("RY 677-685 = 701")
{
 R_Date ("OxA-30899 RY 697-705 = 701", 3498,32);
 R_Date ("OxA-31523 RY 697-705 = 701", 3470,31);
 Outlier ("RScaled",0.05);
```

```
  };
  Gap(75);
  Date ("Ring 776 Estimate");
  Gap (78);
  Date ("Ring 854 Estimate");
 };
};
```

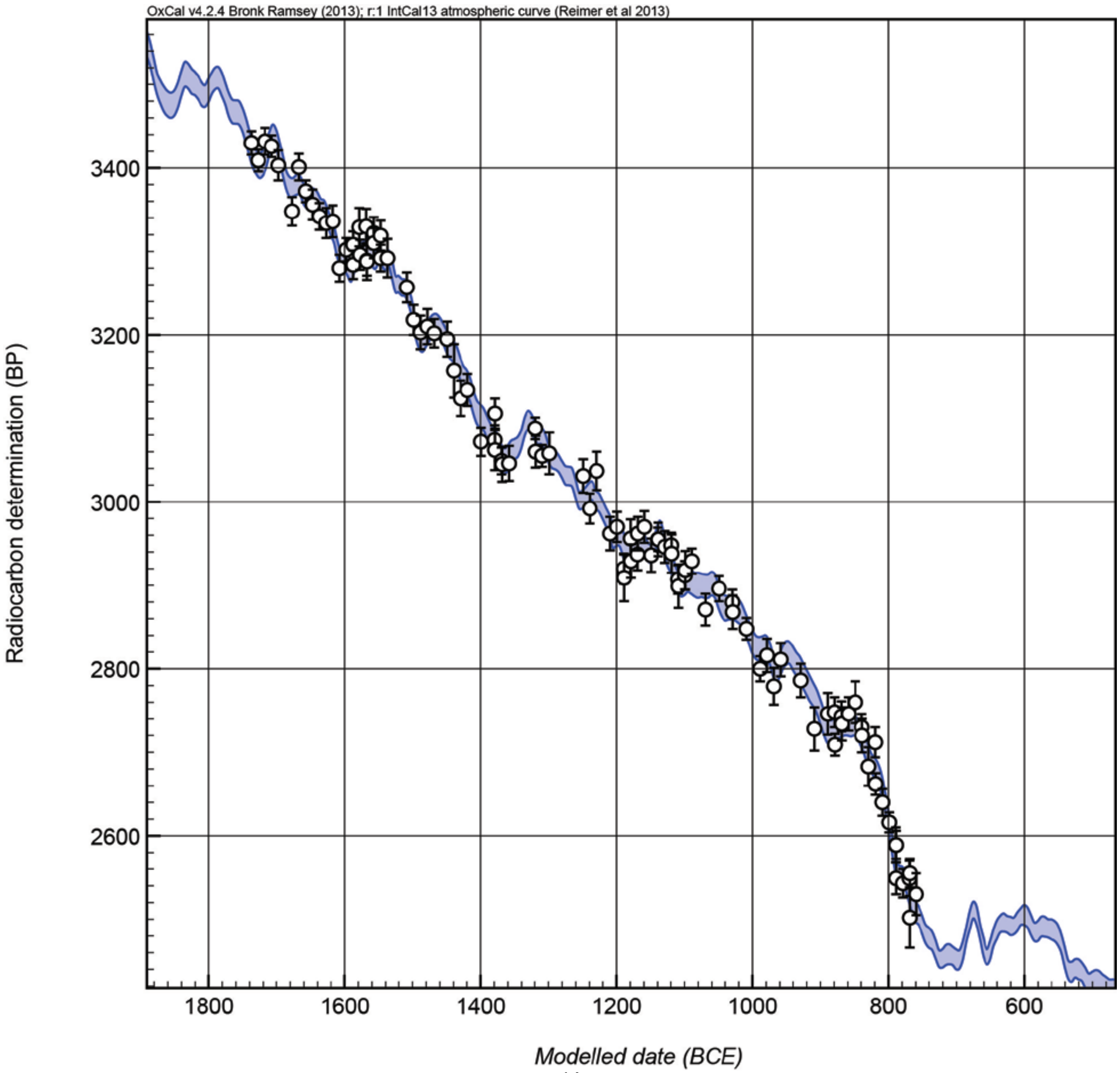

Figure J. The Gordion RY776.5-1764 dendro-^{14}C-wiggle-match minus outliers – Model 3 (Table 2) – shown in terms of the μ±σ placements for all the data from the modelled fit ranges against the IntCal13 ^{14}C calibration curve [61].

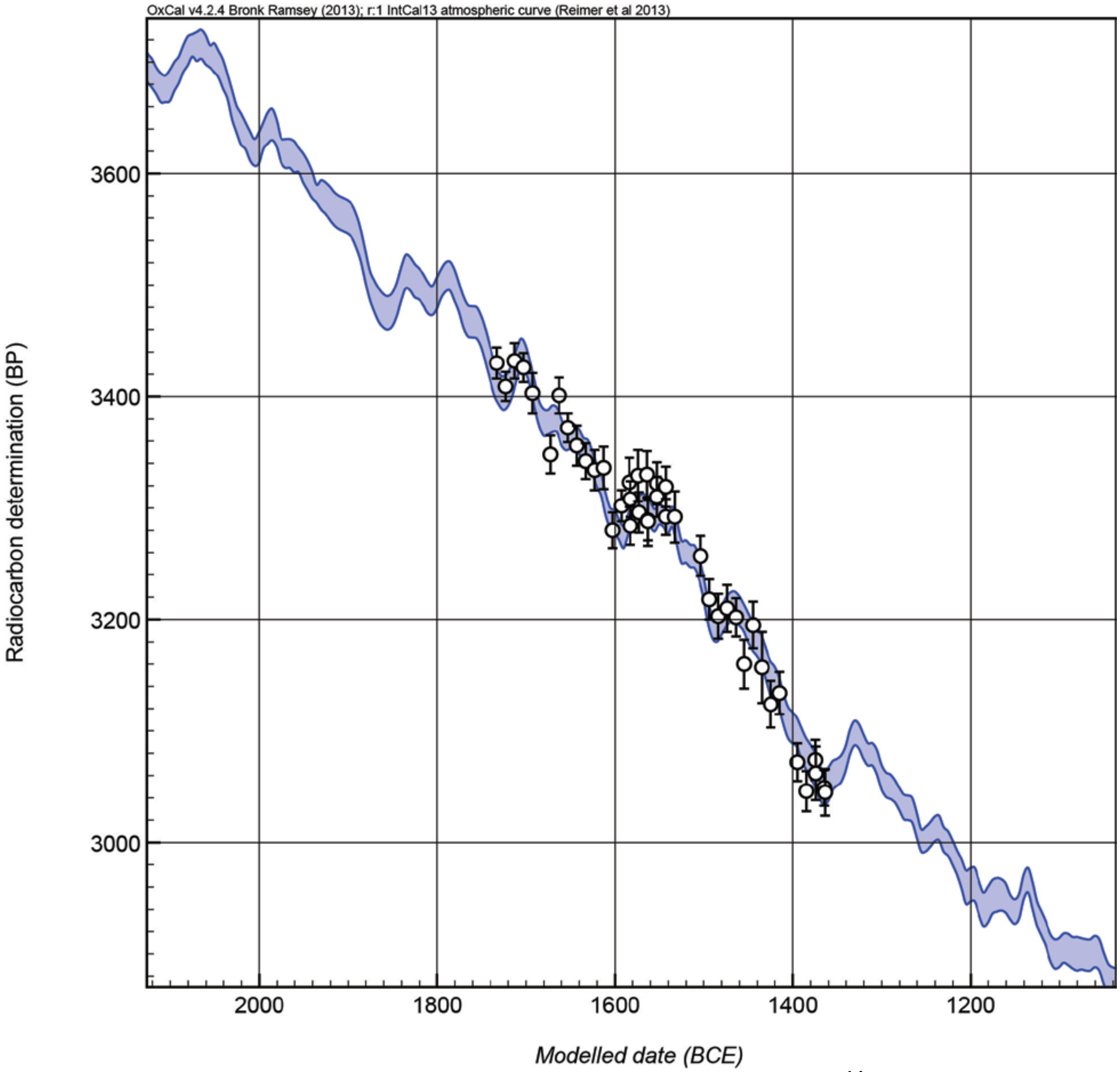

Figure K. The reduced (older only) Gordion RY776.5-1145.5 dendro-^{14}C-wiggle-match minus outliers – Model 5 (Table 2) – shown in terms of the μ±σ placements for all the data from the modelled fit ranges against the IntCal13 ^{14}C calibration curve [61].

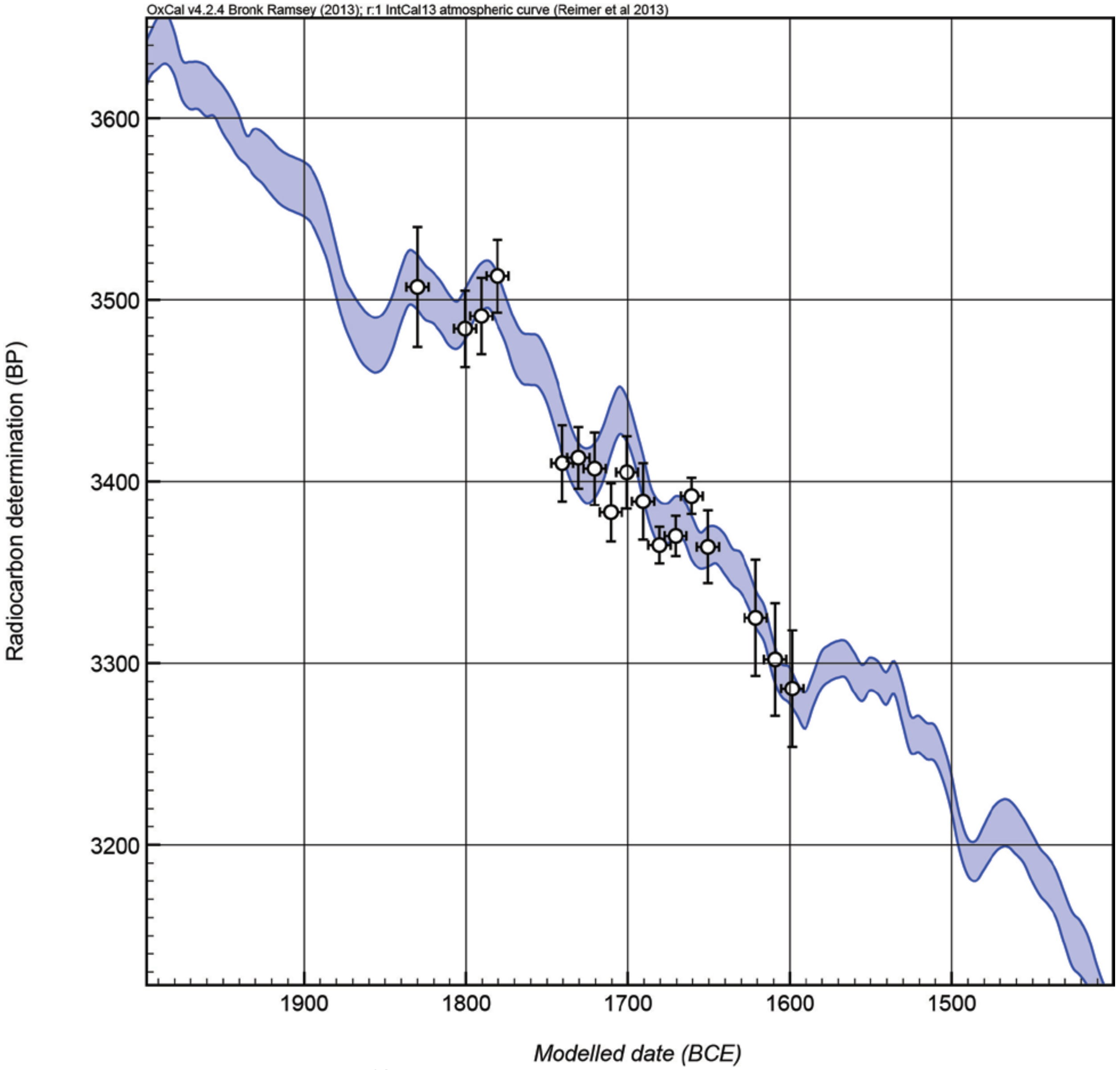

Figure L. The Porsuk dendro-^{14}C-wiggle-match minus outliers – Model 6 (Table 2) – shown in terms of the μ±σ placements for all the data from the modelled fit ranges against the IntCal13 ^{14}C calibration curve [61].

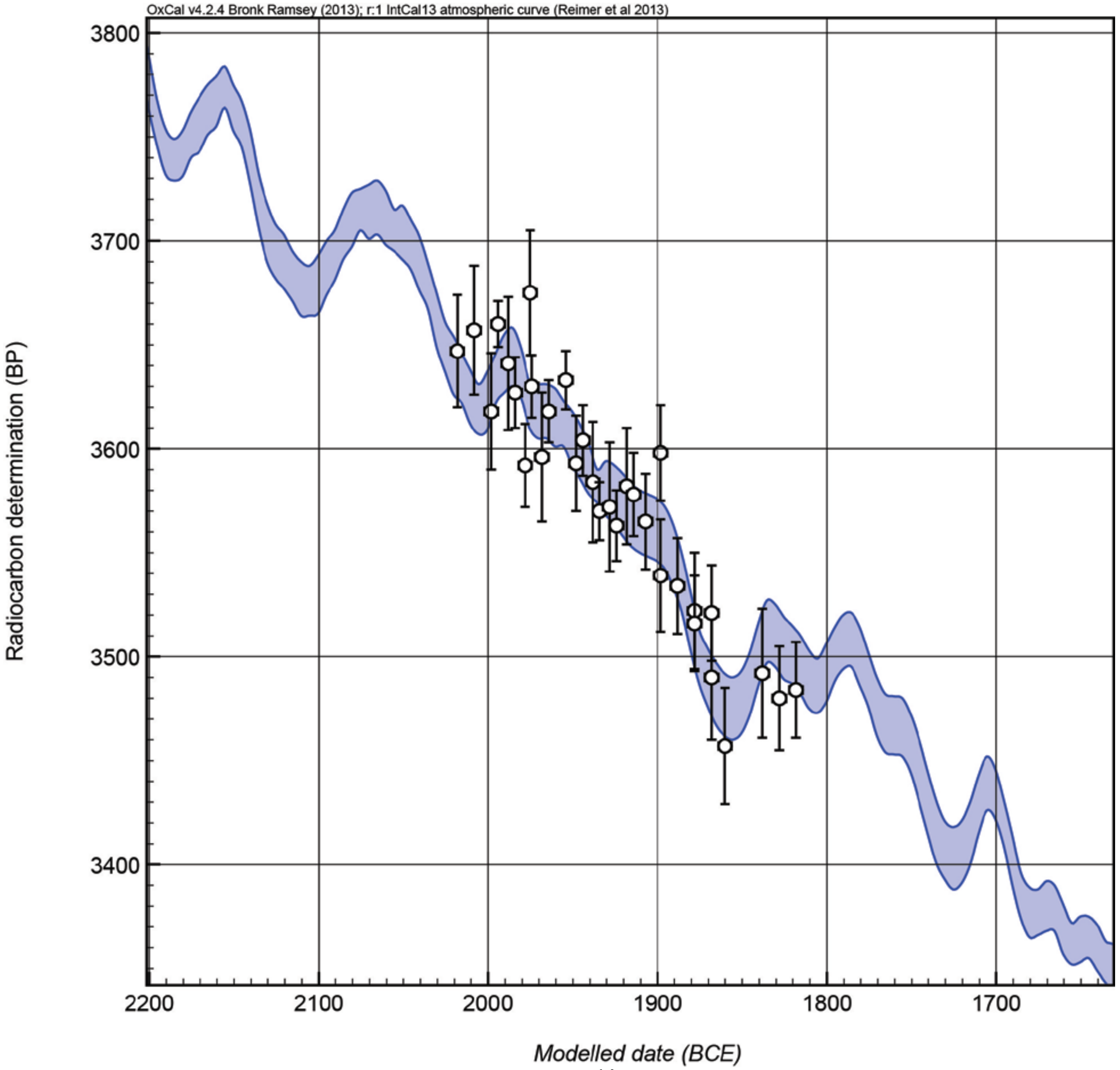

Figure M. The MBA (KUL-KBK-ACM) dendro-^{14}C-wiggle-match minus outliers – Model 8a (Table 2) – shown in terms of the μ±σ placements for all the data from the modelled fit ranges against the IntCal13 ^{14}C calibration curve [61].

One point of detail to note is that in previous work it was argued that use of only the earlier (older, in calendar terms) section of the Gordion series gave a likely better calendar age placement [22, 40]. The situation is less clear-cut with IntCal13 [61], although it remains the case that there is more noise in the later (more recent) part of the series – 21.8% of data after Relative Year (RY) 1145.5 are outliers in Model 1 (all data) versus 14% of data for the earlier section RY 776.5 to 1145.5 in Model 4 (earlier data only): Table 2. We consider both the whole and reduced earlier Gordion datasets (see Fig 5 inset), and the difference in respective calendar placement is now small: ~6 years including outliers and ~4 years once outliers are excluded (Table 2). We observe that the various fits reported all appear good, but we note that some of the apparent outliers in these analyses result from their comparison against the smoothed IntCal13 record – whereas the data nearly all fit comfortably versus the underlying non-modelled (raw) IntCal13 dataset (Fig 5). Regardless of whether we prefer the use of the entire Gordion dataset, or the reduced earlier section (the options shown in Fig 5 and quantified in Table 2), the ^{14}C placement of the Porsuk chronology leaves it as incompatible with the previously stated (and now withdrawn) dendrochronological crossdate.

Figures J-M in S1 File show the modelled best placements ($\mu\pm\sigma$ is shown) for the dendro-sequenced ^{14}C data for Models 3, 5, 6 and 8a from Table 2. In Bayesian Chronological Modelling with large or complex datasets, small variations occur between different runs (usually very minimal when employing fixed dendro-sequenced models as in this project). Table 2, Figs 3, 4, and Figures J-M in S1 File show typical values from several runs. Figure N in S1 File shows the modelled calendar probability distributions and most likely (highest probability density, hpd) 68.2% and 95.4% calendar age ranges, and $\mu\pm\sigma$ and median calendar ages for the placement of RY776 from Models 3, 5, 6 and 8a (as in Table 2, and Figures J-M in S1 File). Note: the tree-ring series do not all include an RY776 – this year is extrapolated in such cases to enable the comparison in terms of a single year.

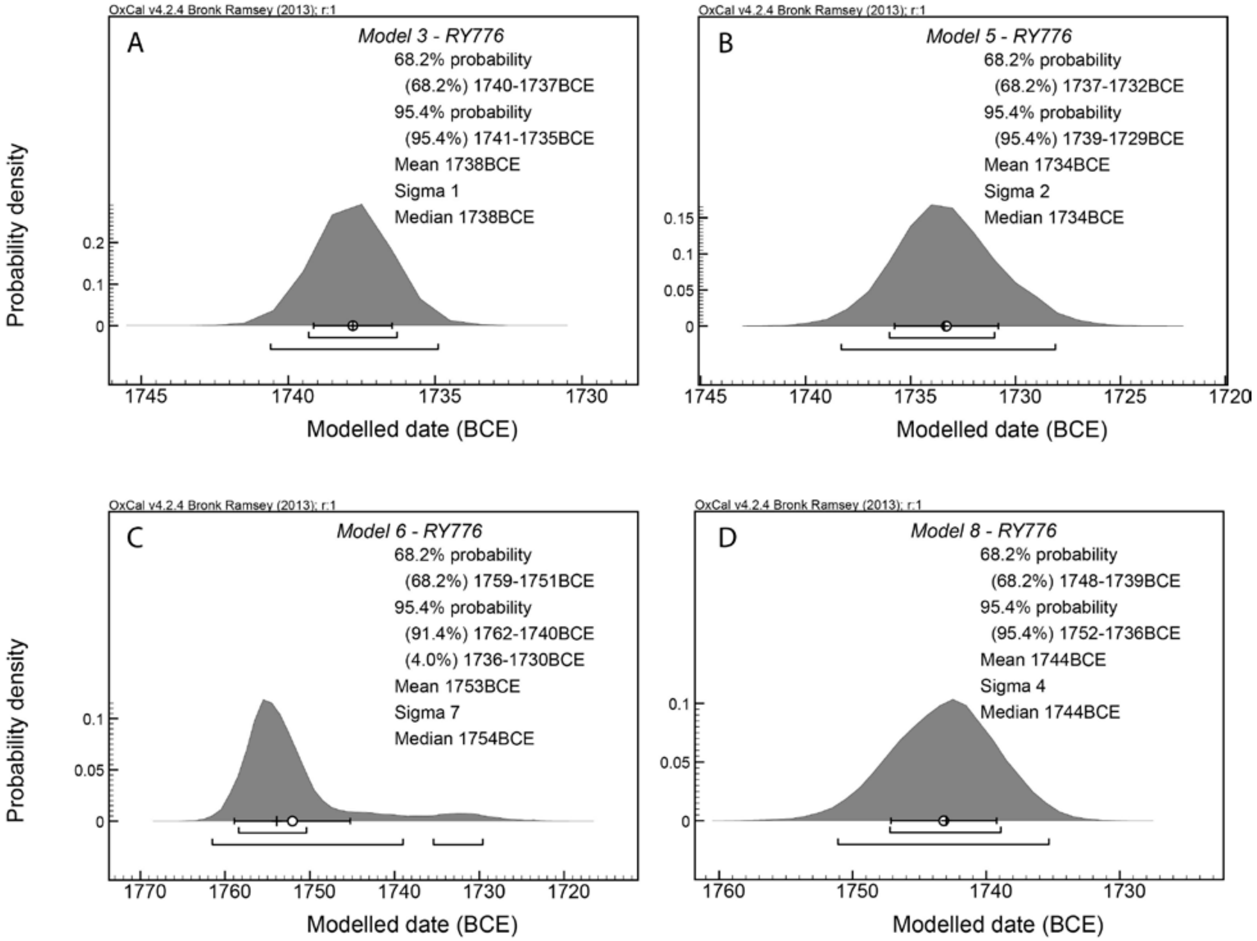

Figure N. Relative Year (RY) 776 placements. The modelled calendar age probability distributions, and most likely (hpd) 68.2% and 95.4% calendar age ranges and the μ±σ (mean ± standard deviation) and median, for the placement of RY776 from Models 3, 5, 6 and 8 (models in Figures J-M in S1 File) and as reported in Table 2. A = Model 3, B = Model 5, C = Model 6, and D = Model 8a.

As noted in the main text, we also considered, as a comparison with the OxCal Bayesian modelling approach, the best fit of the Model 7b data (Table 2) versus IntCal13 [61] via a classical least squares curve fitting approach. The two methods should yield very similar outcomes [22, 43] and the fit found in Figure O in S1 File is only 6 years different (older) than the mean of the best fit probability distribution for Model 7b (Table 2). This comparison, as in previous studies (e.g. [22, 43]), highlights that the calendar best fits calculated for the dendro-^{14}C wiggle-matches in Table 2 by OxCal are robust, and do not rely only on use of Bayesian statistical methods or any particular implementation. In addition, display of this least squares fit versus the IntCal13 modelled curve in Figure O in S1 File allows the chance to visualize the dendro-sequenced ^{14}C data from the MBA chronology data versus not only the modelled IntCal13 curve but also against the constituent data behind the IntCal13 model. We see the MBA data fitting well within the cloud of IntCal13 raw data. Three of the MBA data points are

subjectively observed as particular outliers – more than 3 standard deviations (SD) from the mean IntCal13 value (indicated by orange arrows in Figure O in S1 File). Figure P in S1 File shows the analysis re-run excluding these three data points when calculating the least squares best fit function. The best fit is very similar, but 4 years more recent and only 4 calendar years different (older) than the mean of the best fit probability distribution for Model 8a in Table 2. The data visually offers a good fit in Figure P in S1 File with only a couple of the MBA dates (data points centered at RY 661 and 671) perhaps looking like possible outliers – although here it is relevant to note that there is in fact a relative dearth of constituent IntCal13 data points in this particular area, and so, were there additional data available, the MBA data might not seem so far then from the IntCal13 trend.

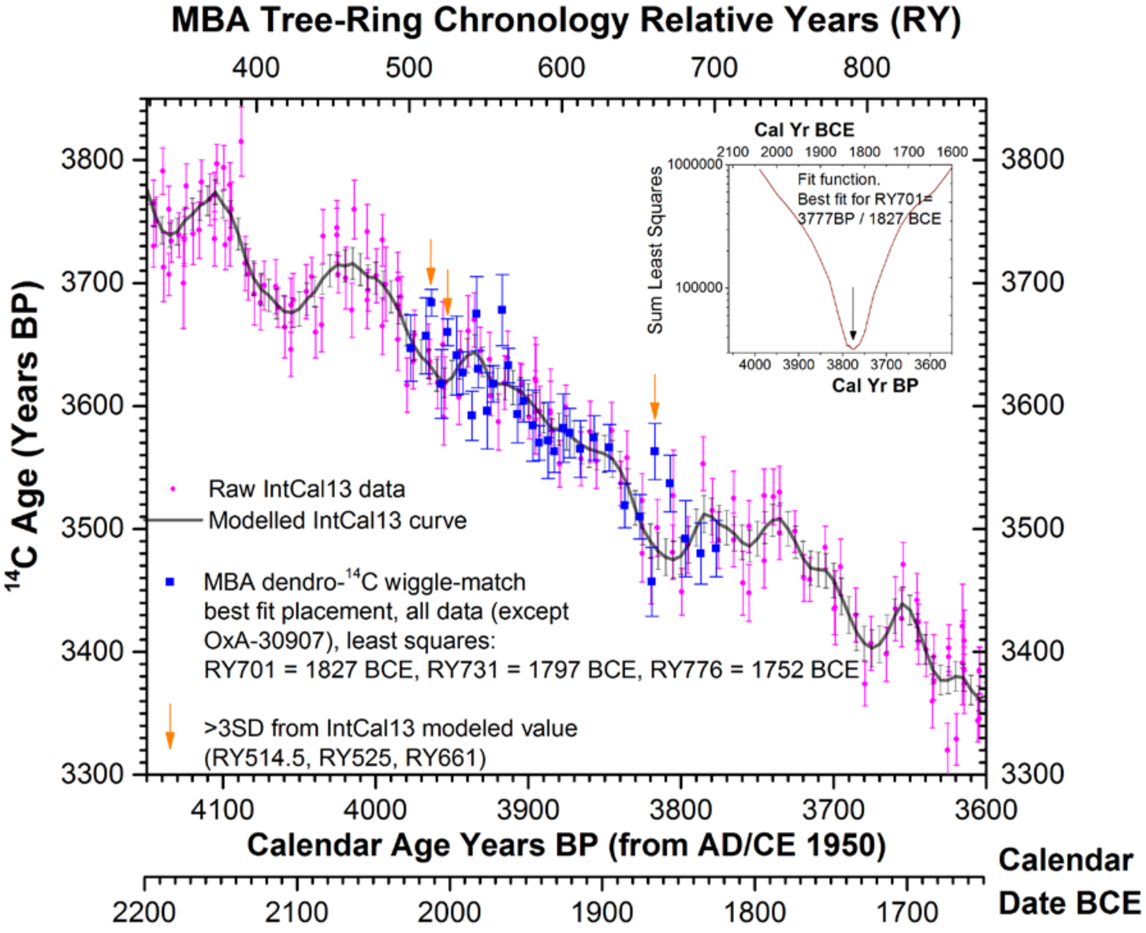

Figure O. Least squares best fit of the Model 7b dataset versus IntCal 13 [61]. All data from the MBA chronology (shown in blue) are employed (except OxA-30907 for RY607-615, which had divergent δ^{13}C values recorded by the AMS versus those independently measured on an MS, and so is considered inherently less than entirely reliable) with dates on the (exactly) contemporary tree-rings (even if from different sites) combined. Three data stand out, subjectively, as outliers and are more than 3 SD from the relevant IntCal13 modelled mean age and are indicated by the orange arrows. The constituent data employed to model the IntCal13 curve are shown by the magenta data points. All error bars are ±1SD. The inset shows the fit function of the least squares analysis with the best fit point, expressed in terms of the mid-point of the last dated sample = RY 701.

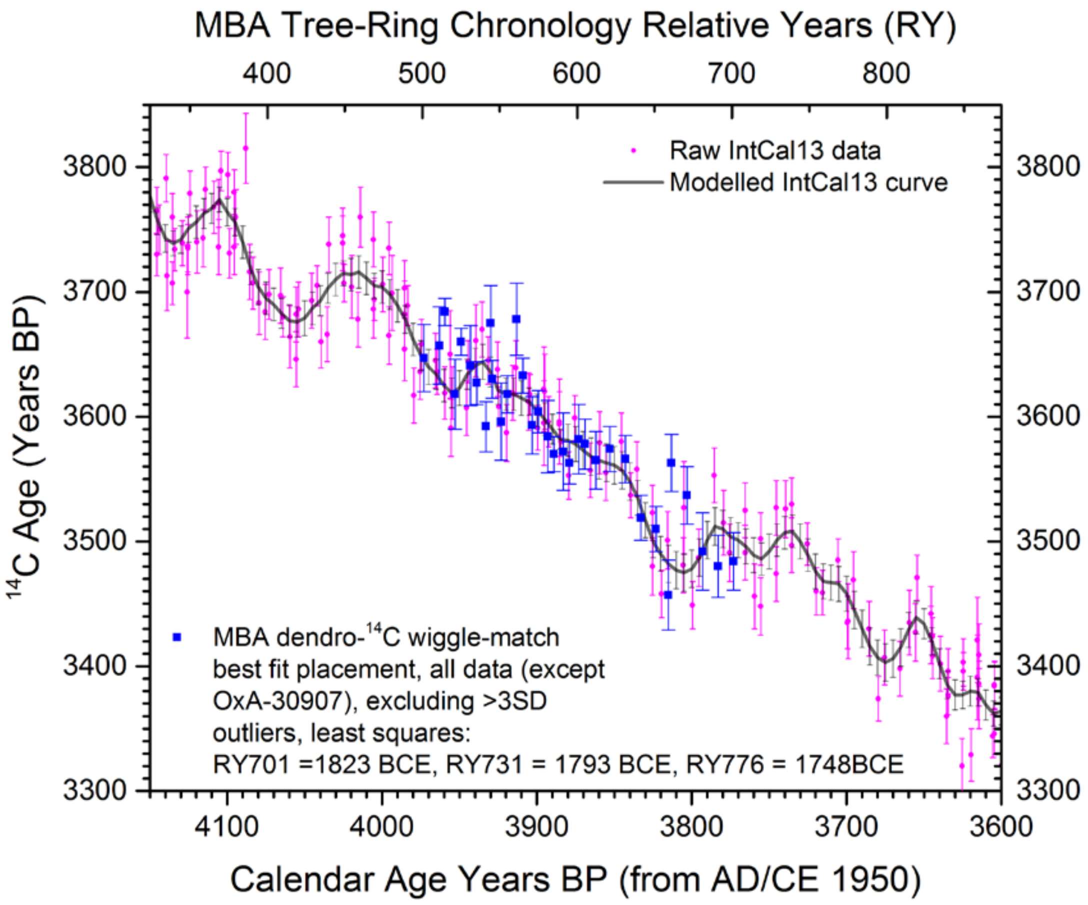

Figure P. As Figure O in S1 File main panel but with the least squares fit calculated excluding the 3 outliers indicated by orange arrows in Figure O in S1 File. All data (including the three 'outliers') are shown. The best fit is 4 years more recent than in Figure O in S1 File.

As a final robustness test, the models after outliers were removed (Models 3, 6, 8a) were run again considering a possible 0±10 ΔR offset test [62]: see Table 2 (Models 3a, 6a, 8c). This was to consider whether there might be a small regional (e.g., growing season related) offset in contemporary ^{14}C levels between the trees growing in Anatolia over this time period versus the trees employed in building the IntCal13 dataset for this time period (which are from southern Germany and Ireland). Although small regional offsets have been observed in the East Mediterranean region at certain specific periods (in particular associated with major solar minima or other climate change episodes) (e.g. [57, Reference E in S1 File]), we find, as in previous work examining this issue more widely in Anatolia and the East Mediterranean (outside of Egypt – and we might hypothesize some parts of the southern Levant) that for most periods including the second millennium BCE [40, Reference F in S1 File], no substantive offset is evident – instead there is either only a very small offset and/or an offset found that is potentially compatible with a 0-year offset. Hence the data reported in Table 2 appear to be robust.

It is important to note in regard to this issue that the hydrologic context in Egypt is very different. In pre-modern, pre-Aswan dam, Egypt the timing of the annual Nile flood led to a growing season that was markedly different (almost opposite) to the usual Northern Hemisphere growing season. This in turn means that the intra-annual differences in contemporary tropospheric ^{14}C levels (winter low to summer high) are evident when comparing Egyptian grown samples (winter growing season) versus the IntCal ^{14}C record reflecting the summer growing season. This offset factor, ΔR 19±5 ^{14}C years [50, Reference G in S1 File] needs to be allowed for when dating sample material which grew in the Nile Valley environment of pre-modern Egypt. No such offset is evident in the Anatolian wood samples from KUL, KBK, ACM, POR or GOR employed in this project for the second millennium BCE (Table 2).

Supporting Information References

A. Schweingruber FH. Anatomy of European woods: an atlas for the identification of European trees, shrubs and dwarf shrubs. Bern: Verlag Paul Haupt; 1990.

B. Eckstein D, Bauch J. Beitrag zur Rationalisierung eines dendrochronologischen Verfahrens und zur Analyse seiner Aussagesicherheir. Forstwissenschaftliches Zentralblatt 1969; 88: 230-250.

C. Buras A, Wilmking M. Correcting the calculation of Gleichläufigkeit. Dendrochronologia 2015; 34: 29-30.

D. Bronk Ramsey C, Higham TFG, Owen DC, Pike AWG, Hedge REM. Radiocarbon dates from the Oxford AMS System; Archaeometry Datelist 31. Archaeometry 2002; 44(3) Supplement 1: 1-149.

E. Manning SW, Dee MW, Wild EM, Bronk Ramsey C, Bandy K, Creasman PP, et al. High-precision dendro-^{14}C dating of two cedar wood sequences from First Intermediate Period and Middle Kingdom Egypt and a small regional climate-related ^{14}C divergence. Journal of Archaeological Science 2014; 46: 401-416.

F. Manning SW, Kromer B. Considerations of the scale of radiocarbon offsets in the east Mediterranean, and considering a case for the latest (most recent) likely date for the Santorini eruption. Radiocarbon 2012; 54:4 49-474.

G. Dee MW, Brock F, Harris SA, Bronk Ramsey C, Shortland AJ, Higham TFG, et al. Investigating the likelihood of a reservoir offset in the radiocarbon record for ancient Egypt. Journal of Archaeological Science 2010; 37: 687-693.
